# Sustained Pax6 Expression Generates Primate-like Basal Radial Glia in Developing Mouse Neocortex

**DOI:** 10.1371/journal.pbio.1002217

**Published:** 2015-08-07

**Authors:** Fong Kuan Wong, Ji-Feng Fei, Felipe Mora-Bermúdez, Elena Taverna, Christiane Haffner, Jun Fu, Konstantinos Anastassiadis, A. Francis Stewart, Wieland B. Huttner

**Affiliations:** 1 Max Planck Institute of Molecular Cell Biology and Genetics, Dresden, Germany; 2 Biotechnology Center of the Technische Universität Dresden, Dresden, Germany; CAS-MPG Partner Institute for Computational Biology, CHINA

## Abstract

The evolutionary expansion of the neocortex in mammals has been linked to enlargement of the subventricular zone (SVZ) and increased proliferative capacity of basal progenitors (BPs), notably basal radial glia (bRG). The transcription factor Pax6 is known to be highly expressed in primate, but not mouse, BPs. Here, we demonstrate that sustaining Pax6 expression selectively in BP-genic apical radial glia (aRG) and their BP progeny of embryonic mouse neocortex suffices to induce primate-like progenitor behaviour. Specifically, we conditionally expressed Pax6 by in utero electroporation using a novel, *Tis21*–CreER^T2^ mouse line. This expression altered aRG cleavage plane orientation to promote bRG generation, increased cell-cycle re-entry of BPs, and ultimately increased upper-layer neuron production. Upper-layer neuron production was also increased in double-transgenic mouse embryos with sustained Pax6 expression in the neurogenic lineage. Strikingly, increased BPs existed not only in the SVZ but also in the intermediate zone of the neocortex of these double-transgenic mouse embryos. In mutant mouse embryos lacking functional Pax6, the proportion of bRG among BPs was reduced. Our data identify specific Pax6 effects in BPs and imply that sustaining this Pax6 function in BPs could be a key aspect of SVZ enlargement and, consequently, the evolutionary expansion of the neocortex.

## Abstract 2

"Humanizing" the expression of the transcription factor Pax6 in cortical progenitors in the developing mouse brain is sufficient to endow these progenitors with a primate-like proliferative capacity.

## Author Summary

During development, neural progenitors generate all cells that make up the mammalian brain. Differences in brain size among the various mammalian species are attributed to differences in the abundance and proliferative capacity of a specific class of neural progenitors called basal progenitors. Among these, a specific progenitor type called basal radial glia is thought to have played an important role during evolution in the expansion of the neocortex, the part of the brain associated with higher cognitive functions like conscious thought and language. In the neocortex, the expression of the transcription factor Pax6 in basal progenitors is low in rodents, but high in primates, including humans. In this study, we aimed to mimic the elevated expression pattern of Pax6 seen in humans in basal progenitors of the embryonic mouse neocortex. To this end, we generated a novel, transgenic mouse line that allows sustained expression of the Pax6 gene in basal progenitors. This elevated expression resulted in an increase in the generation of basal radial glia, in the proliferative capacity of basal progenitors, and, ultimately, in the number of neurons produced. Our findings demonstrate that altering the expression of a single transcription factor from a mouse to a human-like pattern suffices to induce a primate-like proliferative behaviour in neural progenitors, which is thought to underlie the evolutionary expansion of the neocortex.

## Introduction

The evolutionary expansion of the mammalian neocortex is thought to be primarily the consequence of the increasing proliferative capacity of cortical stem and progenitor cells during development [1–9]. Recent studies have progressively focused on differences between species regarding the type, abundance, and modes of division of cortical stem and progenitor cells, which are thought to contribute to the variety of shapes and sizes of the neocortex present across mammals [1–8].

A hallmark of the developing cortical wall is its apical–basal polarity, with the apical side corresponding to the ventricular surface and the basal side contacting the basal lamina [4,10]. At the onset of neurogenesis, neuroepithelial cells, the primary cortical stem cells, transform into apical radial glia (aRG) [11,12]. aRG, together with apical intermediate progenitors, constitute apical progenitors (APs), as they repeatedly undergo mitosis at the apical surface of the cortical wall [8,10]. Apical intermediate progenitors (previously called short neural precursors) undergo self-consuming division generating two neurons [13–15]. In contrast, aRG undergo self-renewing divisions, generating neurons and, more frequently, basal progenitors (BPs) that delaminate from the apical surface, leave the ventricular zone (VZ) and move basally to the subventricular zone (SVZ) [16–24].

BPs comprise basal radial glia (bRG, also called outer radial glia) and basal intermediate progenitors (bIPs) [8,10]. BPs typically undergo mitosis in the SVZ and can undergo, in principle, neurogenic (i.e., neuron-producing) or proliferative (i.e., self-amplifying) divisions, albeit with profound differences in occurrence between species [8,16–18,20–22,25–31]. bRG can be distinguished from the process-lacking bIPs by their apically and/or basally directed processes at mitosis [8,17,18,21–28,31].

Comparison of BPs in various mammalian brains has revealed key differences in their abundance and mode of cell division [1–6,8,32–34]. Thus, such differences have been reported for bIPs, which can be classified into two principal types, neurogenic and proliferative, depending on the mode of cell division (generating two neurons and two bIPs, respectively) [8]. In the mouse and rat SVZ, neurogenic bIPs constitute the vast majority of BPs (>80%) [16–18,21,22], whereas proliferative bIPs and bRG exist in only small proportions [17,28–30,35]. Moreover, mouse bRG typically undergo asymmetric self-renewing neurogenic divisions but not symmetric proliferative divisions [28].

By contrast, in mammals exhibiting an increased abundance of BPs and an enlarged SVZ, as characterized in detail in species such as ferret, macaque, and human [1,4–6,8,23,32], bIPs are mostly of the proliferative type, and bRG constitute at least half of all BPs [23–27]. Moreover, in these species, both bRG and proliferative bIPs undergo mostly symmetric proliferative rather than neurogenic divisions [23,24,31]. These self-amplifying divisions significantly increase the number of BPs residing in the SVZ, consequently leading to the expansion of the SVZ. Moreover, the SVZ of these animals comprises not only a rodent SVZ-related layer called the inner SVZ (iSVZ) but in addition a novel layer called the outer SVZ (oSVZ) [32]. Importantly, these alterations in the mode of cell division and the resulting increase in BP abundance and formation of an oSVZ have been hypothesized to be major causes underlying the expansion of the neocortex [2–6,8,32].

A key question then is how these differences in BP abundance and mode of cell division between rodents and primates are brought about at the molecular level. A candidate regulatory mechanism is the differential expression of transcription factors. Of particular interest in this regard is Pax6 (accession number: AAH36957), a paired-box transcription factor [36–39]. Several mouse and rat mutant models have demonstrated that Pax6 is required for normal aRG abundance and mode of cell division [37,40–49]. Moreover, although Pax6 mRNA levels are generally lower in BPs than APs, this down-regulation is much greater for mouse than human [50]. Consistent with this, only a minority of mouse and rat BPs (<30%) show Pax6 immunoreactivity (which is of lower level than in APs) [3,51,52], whereas the opposite is the case for primate, notably human, BPs (>80% Pax6-positive), with essentially all bRG and the majority of bIPs containing this transcription factor [3,23–27,53,54]. Together, these findings raise the possibility that the differences in Pax6 expression between rodent and primate BPs may be responsible, at least in part, for the greater abundance and proliferative or self-renewal capacity of the latter.

We therefore sought to maintain Pax6 expression specifically in newly generated BPs in order to investigate if such expression would increase the abundance of BPs, notably of bRG, and their proliferative or self-renewal capacity. Using a novel approach of conditional Pax6 expression [16,21,55], we find that sustaining elevated Pax6 levels in BP-genic mouse aRG and the BP progeny derived therefrom increases both the proportion of bRG among the newly generated BPs and the self-renewing capacity of BPs.

## Results

### Expression of a Tamoxifen-Dependent Cre Recombinase in the Neocortex of *Tis21*–CreER^T2^ Knock-in Mouse Embryos Is Specific to Neurogenic Progenitors

**Fig 1:**
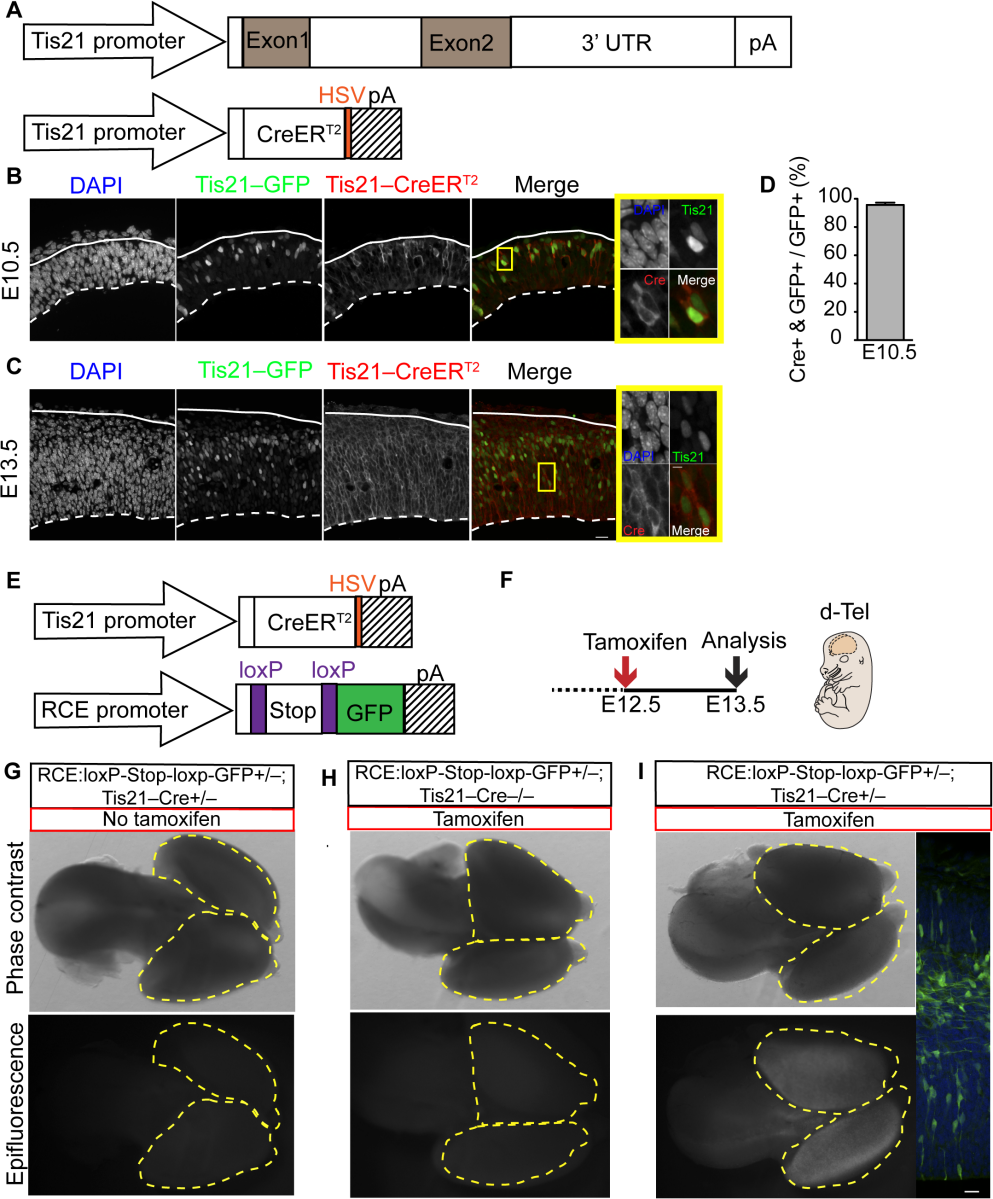
*Tis21–*CreER^T2^ knock-in mouse embryos show tamoxifen-dependent recombination specifically in Tis21-expressing progenitors. (**A**) Cartoon showing the wildtype *Tis21* allele (top) and the knock-in allele in which exon 1 of the *Tis21* gene is replaced by *CreER* ^*T2*^ (bottom). (**B–D**) Cellular distribution of *Tis21*–CreER^T2^. Double-transgenic E10.5 (**B**,**D**) and E13.5 (**C**) mouse embryos carrying the *Tis21–GFP* knock-in and the *Tis21–CreER* ^*T2*^ knock-in alleles. (**B**,**C**) Double immunofluorescence for *Tis21*–GFP (green) and *Tis21*–CreER^T2^, detected via the HSV tag (red), combined with DAPI staining (blue), on coronal 10–12 μm cryosections of dorsolateral telencephalon. Solid and dashed white lines, pial and ventricular surface, respectively; scale bar, 20 μm. Yellow boxes indicate cells coexpressing *Tis21*–GFP and *Tis21*–CreER^T2^ that are shown at higher magnification on the right; scale 5 μm. (**D**) Percentage of *Tis21*–GFP-expressing progenitors that show CreER^T2^ (HSV) immunoreactivity. (**E**–**I**) Specificity of tamoxifen-induced, *Tis21*–CreER^T2^-mediated recombination. (**E**) Cartoon showing the *Tis21*–CreER^T2^ knock-in allele (top) and the *RCE*:*loxP-Stop-loxP-GFP* knock-in allele (bottom). (**F**) Flow scheme of the experiment. (**G–I**) Transgenic E13.5 mouse embryos carrying one *RCE*:*loxP-Stop-loxP-GFP* knock-in allele and either one (+/–, **G**,**I**) or no (–/–, **H**) *Tis21*–CreER^T2^ knock-in allele, with (**H**,**I**) or without (**G**) tamoxifen treatment, as indicated. Whole-brain phase contrast (**G**–**I**) and GFP fluorescence (**I’**). GFP immunofluorescence (green) combined with DAPI staining (blue) on 50-μm vibratome sections of dorsolateral telencephalon (**I’**). Yellow dashed lines, outline of brain hemispheres; solid and dashed white lines, pial and ventricular surface, respectively. Scale bars, 20 μm.

In mouse, the aRG subpopulation that gives rise to BPs, in contrast to self-amplifying aRG, specifically expresses Tis21, a pan-neurogenic progenitor marker [16,21,55]. Thus, as a tool towards maintaining Pax6 expression in mouse BPs, we generated a *Tis21–*CreER^T2^ knock-in mouse line. In this mouse line, exon 1 of *Tis21* is replaced by CreER^T2^ containing a herpes simplex virus (HSV) tag at its C-terminus via homologous recombination (Fig 1A; for details, see S1 Fig), in order to limit Cre expression to Tis21-positive cells. To assess the cellular specificity of Cre expression, *Tis21–*CreER^T2^ knock-in mice were crossed with *Tis21–*GFP knock-in mice [16]. Immunofluorescence of the dorsolateral telencephalon of double-transgenic mice at embryonic day (E) 10.5, corresponding to the onset of *Tis21* expression, and at E13.5, corresponding to the time point at which the in utero electroporations described below were conducted, showed that Cre was expressed in essentially the same cells as GFP (Fig 1B and 1C), indicating its expression selectively in the neurogenic subpopulations of cortical progenitors. Specifically, quantitation at E10.5 revealed that 97% of the cells containing nuclear *Tis21*–GFP were also positive for cytoplasmic Cre (Fig 1D), and no Cre was detected in *Tis21–*GFP-negative cells.

We next ascertained that the *Tis21*–CreER^T2^ mouse exhibits tamoxifen-dependent recombination by crossing this mouse line with a conditionally activateable GFP reporter mouse line, *RCE*:*loxP* [56] (Fig 1E). In these double-transgenic mice, GFP should be expressed only when CreER^T2^ has been translocated from the cytoplasm into the nucleus and excised a stop cassette that prevents the transcription of the *GFP* mRNA; the estrogen analog tamoxifen induces such CreER^T2^ translocation [57]. Indeed, no GFP-positive cells were observed in the absence of tamoxifen (Fig 1G). In contrast, when treated with tamoxifen (Fig 1F), GFP fluorescence was observed throughout the double-transgenic mouse brain (Fig 1I), and GFP-positive cells were found in all layers of the embryonic neocortex (Fig 1I’). This reflected Cre recombinase activity, because no GFP expression was observed when tamoxifen was administered to *RCE*:*loxP* offspring lacking the *Tis21*–CreER^T2^ allele (Fig 1H). We conclude that *Tis21*–CreER^T2^ mouse embryos can be used to obtain tamoxifen-dependent recombination specifically in the neurogenic subpopulations of cortical progenitors.

### *Tis21*–CreER^T2^ Mice Allow Conditional Pax6 Expression Specifically in Neurogenic APs and Their Progeny

**Fig 2:**
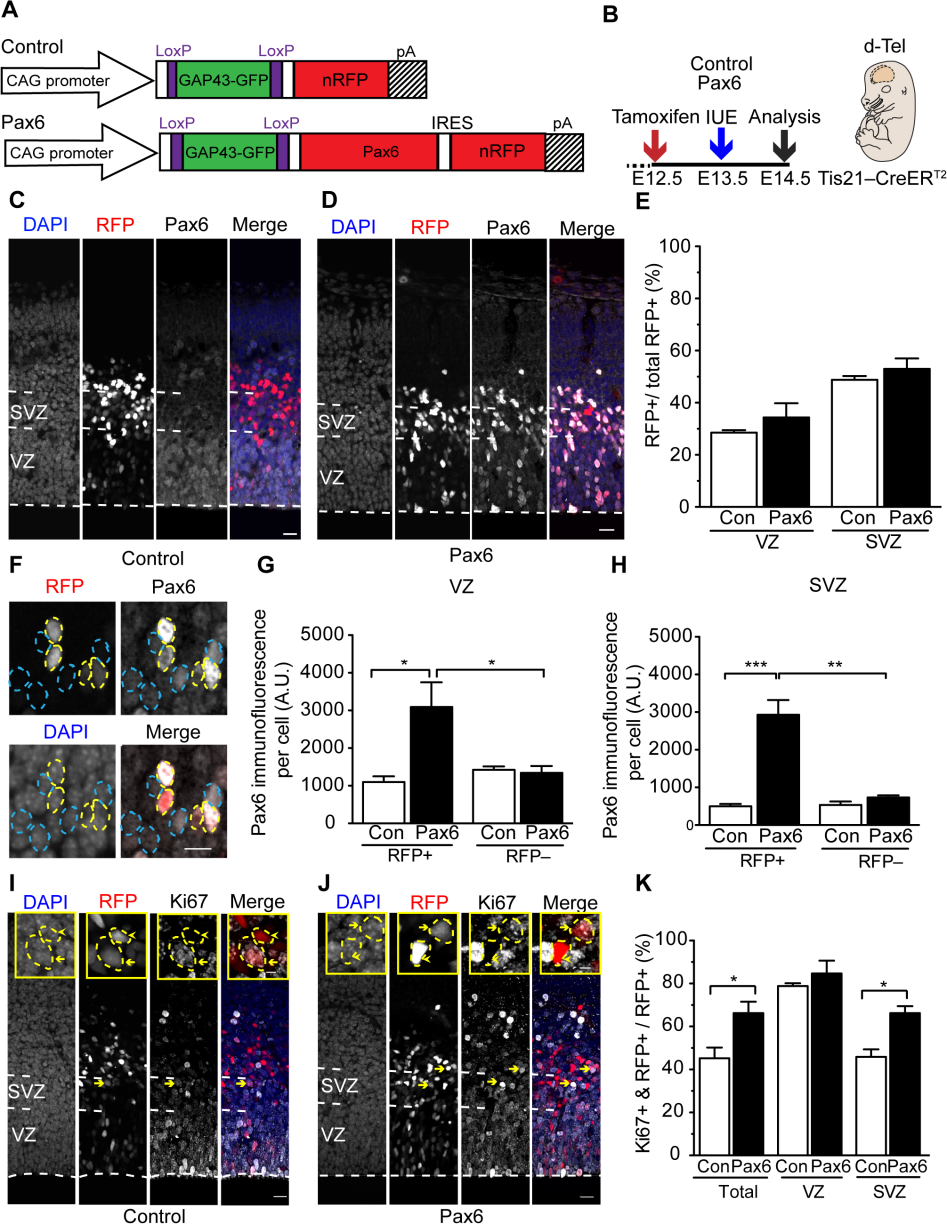
Conditional Pax6 expression specifically in Tis21-positive APs and their progeny increases cycling BPs. **(A)** Cartoon showing the control (top) and Pax6-expressing (bottom) plasmid. **(B)** Flow scheme of experiments. **(C–K)** Dorsolateral telencephalon of tamoxifen-treated E14.5 *Tis21*–CreER^T2^ heterozygous mice electroporated at E13.5 with control (**C**,**E**,**G**,**H**,**I**,**K**) or Pax6-expressing (**D**–**H**,**J**,**K**) plasmid. (**C,D,F**) Pax6 immunofluorescence (white) and RFP fluorescence (red), combined with DAPI staining (blue), on coronal 50-μm vibratome sections. Dashed white lines, ventricular surface; blue and yellow dashed lines, RFP-negative and-positive cells, respectively. Scale bars, 20 μm (**C**,**D**), 10 μm (**F**). (**E**) Quantification of RFP-positive cells in the VZ and SVZ, expressed as percentage of all RFP-positive cells in the cortical wall (200-μm wide area), upon control (Con, white) and Pax6 (black) electroporation. (**G,H**) Pax6 immunofluorescence intensity per cell (A.U., arbitrary units) in VZ (**G**) and SVZ (**H**) upon control (Con, white) and Pax6 (black) electroporation. (**I,J**) Ki67 immunofluorescence (white) and RFP fluorescence (red), combined with DAPI staining (blue), on coronal 50-μm vibratome sections. Dashed white lines, ventricular surface; scale bars, 20 μm. Insets show representative examples of RFP-positive nuclei (outlined by dashed yellow lines) that are either Ki67-positive (yellow arrows, see also main panels) or-negative (yellow arrowheads); scale bars, 5 μm. (**K**) Quantification of Ki67- and RFP-positive cells in the cortical wall (total), VZ and SVZ, expressed as percentage of all RFP-positive cells in the cortical wall (200-μm wide area), upon control (Con, white) and Pax6 (black) electroporation. (**E**,**G**,**H**,**K**) Mean of nine embryos (three independent experiments with three embryos each); error bars, standard error of the mean (SEM). * *p* <0.05, ** *p* <0.01, *** *p* <0.001.

To conditionally express Pax6 in BP-genic aRG of developing neocortex, we introduced a floxed *Pax6* plasmid at midneurogenesis into APs of tamoxifen-treated *Tis21*–CreER^T2^ mouse embryos. Specifically, we generated a plasmid (referred to as Pax6-expressing plasmid) containing a constitutive promoter (CAG) followed by a membrane (GAP43)–GFP cassette flanked by two loxP sites, mouse Pax6, an internal ribosome entry site (IRES) sequence, and nuclear RFP (nRFP) (Fig 2A). Upon Cre-mediated recombination, the membrane–GFP cassette would be excised, leading to the simultaneous expression of Pax6 and nRFP. Introduction of this plasmid into APs of tamoxifen-treated *Tis21*–CreER^T2^ mouse embryos should ensure maintenance of Pax6 expression as mouse BPs arise from aRG divisions, as well as during their subsequent migration to, and function in, the SVZ. An identical plasmid but lacking the Pax6 and IRES sequences served as control (Fig 2A).

We first validated the Pax6-expressing plasmid by transfection of HEK 293T cells, a cell line in which the endogenous *PAX6* gene is not expressed. Transfection with the Pax6-expressing plasmid alone resulted in GFP, but not nRFP, expression. Cotransfection of the Pax6-expressing plasmid and a Cre-expressing plasmid yielded both Pax6 and nRFP expression, whereas only nRFP expression was observed upon cotransfection of the control plasmid and the Cre-expressing plasmid (S2 Fig).

We then explored whether the Pax6-expressing plasmid could be used in *Tis21*–CreER^T2^ mouse embryos to obtain conditional Pax6 expression specifically in the neurogenic subpopulation of APs and their progeny. To this end, we used the in utero electroporation technique where an electric field is generated across the cortical wall in order to allow for the unidirectional delivery of the negatively charged plasmid DNA, injected into the ventricular lumen, into APs. Dorsolateral telencephalon of tamoxifen-pretreated (E12.5) *Tis21*–CreER^T2^ mice was electroporated with Pax6-expressing plasmid at E13.5 and analyzed at E14.5, the peak of BP generation from neurogenic aRG [22] (Fig 2B). For the ease of presentation, we shall refer to this approach from here onwards simply as conditional Pax6 expression. Analysis of the Pax6 expression pattern yielded the following observations.

First, analysis of the level of Pax6 immunoreactivity revealed that a subpopulation of cells had higher Pax6 immunoreactivity upon conditional Pax6 expression than in the control (Fig 2C and 2D). Upon closer inspection, all these highly Pax6-immunoreactive cells were RFP-positive, indicating that these cells constituted Pax6-expressing-plasmid–electroporated neurogenic APs and their progeny (Fig 2C,2D and 2F). The level of Pax6 immunoreactivity in these cells in the VZ was approximately 3-fold higher than that of the nonelectroporated APs or control-plasmid–electroporated neurogenic APs and their VZ progeny (Fig 2G), essentially all of which are known to express endogenous Pax6 [37,51,52]. In the SVZ, where mouse BPs normally down-regulate Pax6 expression [3,50–52], this difference was even greater (≈6-fold higher) (Fig 2H).

Second, the appearance of these highly Pax6-immunoreactive and RFP-positive cells upon Pax6-expressing plasmid electroporation was strictly dependent on tamoxifen pretreatment (S3 Fig). Together, these observations allow us to equate the RFP-positive cells with the cells containing Pax6 due to the electroporation. To distinguish these conditionally Pax6-expressing cells from the cells expressing Pax6 endogenously, we shall refer to them from here onwards as exogenous Pax6- (exoPax6-) expressing cells. In addition, considering the results shown in Fig 1, we conclude that these cells constitute specifically the neurogenic subpopulation of APs and their progeny, notably the aRG-derived BPs.

Third, we found that electroporation with Pax6-expressing plasmid did not affect, after 24 h, the distribution of the progeny (RFP+ cells) of the electroporated neurogenic APs between (Fig 2C–2E) and within (S4 Fig) the germinal layers (i.e., VZ and SVZ). This implies that conditional Pax6 expression in neurogenic APs and their progeny, even if this expression exceeds the normal endogenous level, does not cause any overt effects on cell migration within the first 24 h after electroporation. The finding that RFP-positive cells are similarly distributed in control and upon conditional Pax6 expression allows for a valid comparison between germinal layers of the effect of conditional Pax6 expression in subsequent experiments.

Conditional Pax6 expression in aRG has previously been found to induce apoptosis when pan-aRG Cre drivers based on *Emx1* and *hGFAP* promoter and regulatory sequences were used. However, this phenomenon was not observed with a Cre driver based on *Ngn2* expression [58], which, similar (but not identical) to *Tis21* expression, is characteristic of neurogenic progenitors [59]. It was therefore important to ascertain that conditional expression of Pax6 in *Tis21–*CreER^T2^ mice would not induce apoptosis. Indeed, immunofluorescence for the apoptosis marker activated caspase-3 did not reveal any significant difference in the number of caspase-3–positive cells between the progeny of control-plasmid–and Pax6-expressing-plasmid–electroporated neurogenic APs (S5 Fig). We therefore conclude that the present approach of conditional Pax6 expression is suitable to maintain high levels of Pax6 expression specifically in neurogenic APs and their progeny, notably the aRG-derived BPs, thus recapitulating the Pax6 expression pattern observed in BPs of developing primate neocortex.

### Conditional Pax6 Expression Increases Cycling BPs

In assessing the functional consequences of sustained Pax6 expression in BPs, we sought to obtain initial clues as to the identity of the progeny of the Pax6-electroporated neurogenic APs. Using the cycling cell marker Ki67, we first investigated whether the exoPax6-expressing cells exhibited the same proportion of progenitors versus neurons as control cells (Fig 2I–2K). Whereas conditional Pax6 expression did not alter the percentage of Ki67-positive cells in the VZ, it did result in a significant increase in Ki67-positive cells in the SVZ (Fig 2K). This suggested that the conditional Pax6 expression increased the population of cycling BPs derived from electroporated aRG.

We noticed in some experiments that in both control and conditional Pax6 expression, more Ki67-positive cells were observed in the basal region of the SVZ, and in particular in the intermediate zone of the electroporated area, but not in the contralateral area nor in nonelectroporated dorsolateral telencephalon. This reflected a previously described side effect of in utero electroporation, that is, the displacement of some Pax6-positive cells towards the cortical plate [60]. Importantly, this side effect does not affect the findings described in the present study for three reasons. First, all our data are comparisons between control and conditional Pax6 expression, both of which involve identical conditions of in utero electroporation. Second, all our quantifications are confined to electroporated, RFP-positive cells, and the electroporation side effect has been reported to affect mainly nonelectroporated cells [60]. Third, our quantifications of cells in the SVZ exclude cells in the intermediate zone.

### Conditional Pax6 Expression Increases the Proportion of Cortical Progenitors in S-phase

To gain further insight into a possible regulation of the cell cycle of cortical progenitors by conditional Pax6 expression, we examined specific cell cycle parameters. We first examined the effect of conditional Pax6 expression on the total cell cycle length (Tc) of neurogenic aRG by performing live imaging on E14.5 organotypic slices prepared from control or Pax6-expressing plasmid–electroporated brains. The time period between two successive aRG mitoses was taken to indicate the length of the cell cycle, Tc. In both control and conditional Pax6 expression, we observed no major difference in Tc, although there was a trend for a shorter Tc upon conditional Pax6 expression (control, 21.0 ± 3.3 h, *n* = 8 cells versus Pax6, 18.5 ± 1.2 h, *n* = 9 cells, S1 Table top).

**Fig 3:**
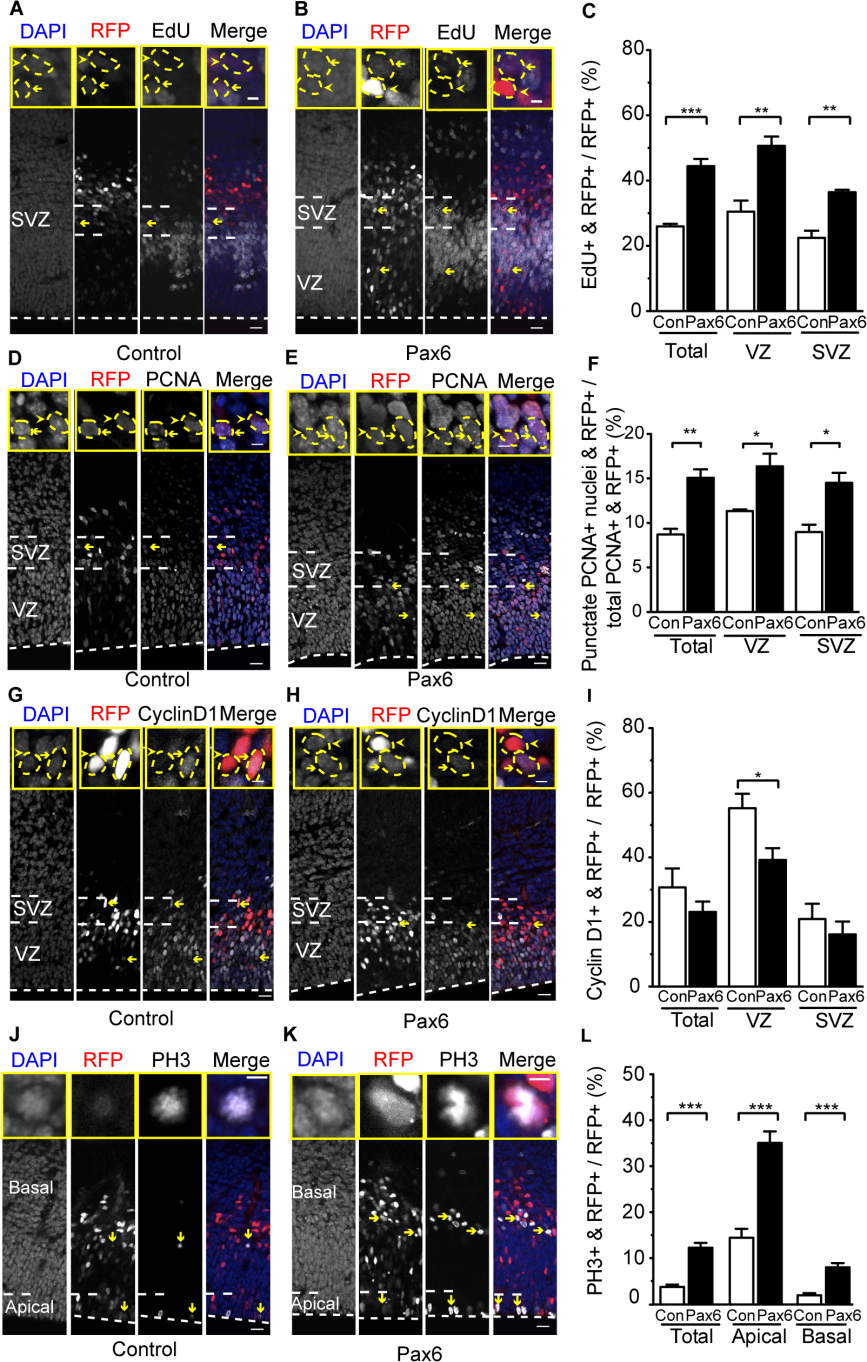
Conditional Pax6 expression in Tis21-positive APs increases S-phase in the AP and BP progeny. Dorsolateral telencephalon of tamoxifen-treated E14.5 *Tis21*–CreER^T2^ heterozygous mice electroporated at E13.5 with control (**A**,**C**,**D**,**F**,**G**,**I**,**J**,**L**) or Pax6-expressing (**B**,**C**,**E**,**F**,**H**,**I**,**K**,**L**) plasmid (see Fig 2B). (**A**–**C**) EdU was administered one hour before sacrifice. (**A**,**B**) EdU (white) and RFP (red) fluorescence, combined with DAPI staining (blue), on coronal 50-μm vibratome sections. Insets show representative examples of RFP-positive nuclei (outlined by dashed yellow lines) that are either EdU-positive (yellow arrows, see also main panels) or-negative (yellow arrowheads). (**C**) Quantification of EdU- and RFP-positive cells in the cortical wall (total), VZ and SVZ, expressed as percentage of all RFP-positive cells in the cortical wall (200-μm wide area), upon control (Con, white) and Pax6 (black) electroporation. (**D**,**E**) Proliferating cell nuclear antigen (PCNA) immunofluorescence (white) and RFP fluorescence (red), combined with DAPI staining (blue), on coronal 12-μm cryosections. Insets show representative examples of RFP-positive nuclei (outlined by dashed yellow lines) that show either a punctate immunoreactivity (yellow arrows, see also main panels), indicative of S-phase, or a diffuse immunoreactivity (yellow arrowheads), indicative of G1 or G2. (**F**) Quantification of punctate PCNA-positive nuclei and RFP-positive cells in the cortical wall (total), VZ and SVZ, expressed as percentage of all PCNA- and RFP-positive cells in the cortical wall (200-μm wide area), upon control (Con, white) and Pax6 (black) electroporation. (**G,H**) Cyclin D1 immunofluorescence (white) and RFP fluorescence (red), combined with DAPI staining (blue), on coronal 12-μm cryosections. Insets show representative examples of RFP-positive nuclei (outlined by dashed yellow lines) that are either cyclin D1-positive (yellow arrows, see also main panels) or-negative (yellow arrowheads). (**I**) Quantification of cyclin D1- and RFP-positive cells in the cortical wall (total), VZ and SVZ, expressed as percentage of all RFP-positive cells in the cortical wall (200-μm wide area), upon control (Con, white) and Pax6 (black) electroporation. (**J,K**) Phosphohistone H3 (PH3) immunofluorescence (white) and RFP fluorescence (red), combined with DAPI staining (blue), on coronal 50-μm vibratome sections. Insets show representative examples of RFP-positive mitotic figures that are PH3-positive. Yellow arrows indicate representative examples of RFP-positive mitotic APs and BPs. (**L**) Quantification of PH3- and RFP-positive cells in the cortical wall (total), at the ventricular surface (apical) and at abventricular locations in the VZ and in the SVZ (basal), expressed as percentage of all RFP-positive cells in the cortical wall (200-μm wide area), upon control (Con, white) and Pax6 (black) electroporation. (**A**,**B**,**D**,**E**,**G**,**H**,**J**,**K**) Dashed white lines, ventricular surface. Scale bars, 20 μm; inset scale bars, 5 μm. (**C**,**I**,**L**) Mean of three independent experiments, each being the average of three embryos; (**F**) mean of three embryos (control) or four embryos (Pax6). Error bars, SEM. * *p* <0.05, ** *p* <0.01, *** *p* <0.001.

To estimate the proportion of the progeny of control-plasmid–and Pax6-expressing-plasmid–electroporated neurogenic APs that were in S-phase, we performed pulse-labeling with the thymidine analog EdU one hour before analyzing the embryos at E14.5. This revealed that a significantly greater proportion of the exoPax6-expressing progeny than of the control progeny was in S-phase, in both the VZ and SVZ (Fig 3A–3C). Given that conditional Pax6 expression did not increase the population size of cycling APs (Fig 2K), nor alter much their Tc (S1 Table top), the increase in the proportion of cells in S-phase in the VZ (Fig 3C) likely reflected a greater share of S-phase in the AP cell cycle, rather than an increase in cycling APs as such.

To address this directly, we performed a dual pulse chase experiment as previously described [61] (see S6A Fig and Materials and Methods) in order to determine the length of S-phase. We observed a significant increase in the length of S-phase for the sum of the electroporated aRG and their progeny upon conditional Pax6 expression (S6 Fig).

We further corroborated this by analyzing the pattern of immunofluorescence of the cycling cell marker proliferating cell nuclear antigen (PCNA). Like other cycling cells, cortical progenitors in S-phase show a punctate nuclear PCNA pattern, whereas progenitors in G1 and G2 show diffuse nuclear PCNA immunoreactivity [23,52,62]. Based on punctate PCNA staining, we observed a proportion of neurogenic APs in S-phase upon control electroporation that was similar to previously published data on E14.5 Tis21-positive APs [52] (S1 Table middle). Conditional Pax6 expression, however, was found to significantly increase the percentage of PCNA-positive nuclei in the VZ that showed a punctate pattern (Fig 3D–3F), i.e., increased the proportion of neurogenic APs that were in S-phase. These findings, together with the Ki67 (Fig 2K) and EdU (Fig 3C) data, imply that conditional Pax6 expression increases the relative proportion of S-phase within the AP cell cycle.

As there was no significant difference in Tc but an increase in the proportion of cells in S-phase upon conditional Pax6 expression in Tis21-positive APs, we hypothesized that the G1-phase must have been shortened to compensate for the longer S-phase. Consistent with this hypothesis, a significantly smaller proportion of the exoPax6-expressing progeny in the VZ than of the control progeny of electroporated neurogenic APs was positive for cyclin D1, a cyclin that is expressed from mid- to late-G1 (Fig 3G–3I). To estimate the length of the G1-phase, we combined the data obtained from live imaging with the punctate PCNA staining data (S1 Table bottom). As none of the apical mitoses observed lasted for >1 h and no difference in G2 length was reported between neural progenitors [52], we assumed that the proportion of neurogenic aRG in G2- and M-phase remained unchanged upon conditional Pax6 expression. Similar to the data obtained for cyclin D1 (Fig 3I), we estimated a shorter G1-phase upon conditional Pax6 expression (control 15.6 h versus Pax6 12.8 h, S1 Table bottom).

As to BPs, the increase in the proportion of EdU-positive cells in the SVZ upon conditional Pax6 expression (Fig 3C) was consistent with that of Ki67-positive cells (Fig 2K), corroborating our conclusion that the population size of cycling BPs derived from electroporated aRG was increased under this condition. Further support for this population size increase was provided by immunofluorescence for phosphohistone H3, a marker of cells in late G2- and M-phase, which revealed a significant increase in mitotic BPs derived from electroporated aRG (Fig 3J–3L). Also in the case of BPs, conditional Pax6 expression significantly increased the relative proportion of S-phase within the cell cycle as revealed by the pattern of nuclear PCNA immunoreactivity (Fig 3D–3F), albeit not at the expense of decreasing the relative proportion of G1 (Fig 3I).

### Conditional Pax6 Expression Induces Tis21-Expressing aRG to Increasingly Generate BPs with Radial Glia Characteristics

Our group previously reported a difference in S-phase length between Tis21- positive and Tis21-negative APs [52]. As Tis21-negative and Tis21-positive APs differ in the type of division (symmetric versus asymmetric) and progeny produced (APs versus BPs) [16,55], we wondered whether the increase in the relative proportion of S-phase within the cell cycle of the exoPax6-expressing APs (Fig 3F) may be indicative of an alteration in their mode of division.

**Fig 4:**
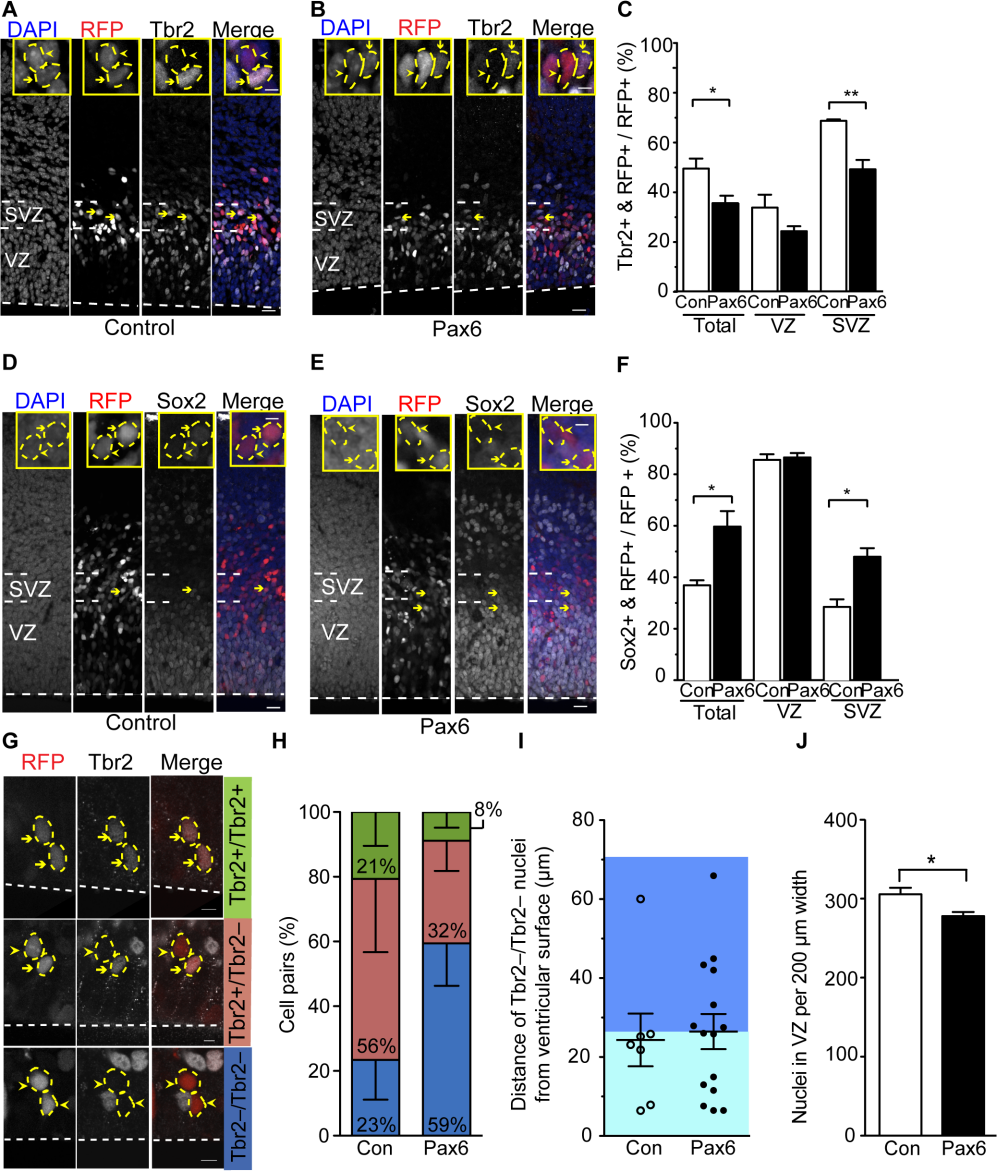
Conditional Pax6 expression in Tis21-positive APs changes the fate of the BP progeny. Dorsolateral telencephalon of tamoxifen-treated E14.5 *Tis21*–CreER^T2^ heterozygous mice electroporated at E13.5 with control (**A**,**C**,**D**,**F**,**H**–**J**) or Pax6-expressing (**B**,**C**,**E**,**F**–**J**) plasmid (see Fig 2B). (**A**,**B**,**D**,**E**) Tbr2 (**A**,**B**) or Sox2 (**D**,**E**) immunofluorescence (white) and RFP fluorescence (red), combined with DAPI staining (blue), on coronal 50-μm vibratome sections. Insets show representative examples of RFP-positive nuclei (outlined by dashed yellow lines) that are either Tbr2 (**A**,**B**) or Sox2 (**D**,**E**)-positive (yellow arrows, see also main panels) or-negative (yellow arrowheads). (**C**,**F**) Quantification of Tbr2- and RFP-positive cells (**C**) or Sox2- and RFP-positive cells (**F**) in the cortical wall (total), VZ, and SVZ, expressed as percentage of all RFP-positive cells in the cortical wall (200-μm wide area), upon control (Con, white) and Pax6 (black) electroporation. (**G**–**I**) Daughter cell pair analysis. (**G**) Examples of daughter cell pairs derived from Pax6-electroporated APs. Tbr2 immunofluorescence (white) and RFP fluorescence (red) on 12-μm cryosections. (Top) Tbr2+/Tbr2+, (middle) Tbr2+/Tbr2–, (bottom) Tbr2–/Tbr2–; yellow arrowheads, Tbr2–; yellow arrows, Tbr2+; yellow dashed lines, daughter cell pair nuclei. (**H**) Quantification of AP-derived daughter cell pairs upon control and Pax6 electroporation. Blue, Tbr2–/Tbr2–; red, Tbr2–/Tbr2+; green, Tbr2+/Tbr2+. Control, 23 pairs; Pax6, 24 pairs. (**I**) Distance of nuclei of the Tbr2–/Tbr2– daughter cell pairs from the ventricular surface upon control (Con, open circles, 7 pairs) and Pax6 (filled circles, 15 pairs) electroporation. Data indicate the position of the ventricular-most nucleus of each pair (see Materials and Methods). Light and dark blue background indicates ventricular (within <27 μm from the ventricular surface) and abventricular (≥27 μm from the ventricular surface) location, respectively. (**J**) Number of nuclei in the VZ (200-μm-wide area) upon control (Con, white) and Pax6 (black) electroporation. (**A**,**B**,**D**,**E**,**G**) Dashed white lines, ventricular surface. Scale bars, 20 μm; inset scale bars, 5 μm. (**C**,**F**,**H**,**I**,**J**) Mean of three independent experiments, each being the average of two to four embryos. Error bars, SEM. * *p* <0.05, ** *p* <0.01.

To explore this possibility, we investigated the nature of the cycling BPs that were increasingly observed upon conditional Pax6 expression (Fig 2I–2K) by examining the expression of two characteristic transcription factors, Tbr2 (Fig 4A–4C) and Sox2 (Fig 4D–4F). Tbr2 is typically expressed by the differentiating progeny of Tis21-expressing aRG fated to become bIPs [22,51,52,63], whereas Sox2 expression is characteristic of aRG and bRG [23,24,26,28,29,31,64]. Upon conditional Pax6 expression, analysis for the abundance of Tbr2-positive cells revealed a significant reduction in the exoPax6-expressing progeny as compared to control (Fig 4A–4C). This reduction was largely accounted for by the decrease in Tbr2-positive cells in the SVZ, most of which presumably were bIPs (Fig 4C). Conversely, the abundance of Sox2-positive cells was higher in the exoPax6-expressing progeny as compared to the control (Fig 4D–4F). Remarkably, this increase occurred in the SVZ rather than the VZ (Fig 4F). This suggested that conditional Pax6 expression, which increased the population of BPs (Fig 2K), induced Tis21-expressing aRG to increasingly generate BPs with a radial glia-characteristic transcription factor expression (i.e., bRG), at the expense (at least relatively) of bIP production.

To directly investigate a possible effect of conditional Pax6 expression on the mode of cell division of neurogenic APs, we performed a daughter cell pair assay [65] by analyzing areas of dorsolateral telencephalon that contained only a few RFP-positive cells in the VZ 24 h after electroporation. Tbr2 immunofluorescence allowed us to distinguish three types of RFP+ daughter cell pairs: (1) Tbr2–/Tbr2– (no bIP daughter cells), (2) Tbr2+/Tbr2– (1 bIP daughter cell) and (3) Tbr2+/Tbr2+ (2 bIP daughter cells) (Fig 4G). Importantly, virtually all Tbr2– daughter cells in the VZ are likely to be radial glia, either aRG or newborn bRG, based on the following considerations. Essentially all daughter cell nuclei in the VZ were PCNA-positive (S7 Fig). This was in line with the findings that >80% and almost 90% of the progeny in the VZ that was derived from electroporated neurogenic APs were Ki67+ (Fig 2K) and Sox2+ (Fig 4F), respectively. Hence, the Tbr2– daughter cells were radial glial progenitors rather than neurons. Consistent with this, almost all cells in the mouse E14.5 VZ are cycling [52], and very few of them are newborn neurons [52].

Quantification of daughter cell pairs in the VZ showed that in the control, the majority (77%) of these pairs derived from AP divisions that had generated bIPs. Specifically, 56% of divisions were asymmetric (and presumably self-renewing) (Tbr2+/Tbr2–, Fig 4H, red), and 21% symmetric self-consuming (Tbr2+/Tbr2+, Fig 4H, green). These findings were in line with the fact that the progeny specifically of neurogenic APs was analyzed. Of note, only 23% of divisions did not generate any bIPs and hence were presumably symmetric proliferative with regard to the radial glia nature of the daughter cells (Tbr2–/Tbr2–, Fig 4H, blue). In contrast, upon conditional Pax6 expression, the majority (59%) of the daughter cell pairs were derived from neurogenic AP divisions that did not generate bIPs but radial glia (Tbr2–/Tbr2–, Fig 4H, blue). This occurred at the expense of bIP-generating divisions, that is, asymmetric self-renewing divisions (Tbr2+/Tbr2–, reduced to 32%, Fig 4H, red), and symmetric self-consuming divisions (Tbr2+/Tbr2+, reduced to 8%, Fig 4H, green).

The observations that conditional Pax6 expression increased (i) the non-bIP generating divisions (Tbr2–/Tbr2–, Fig 4H, blue) and (ii) the Sox2-positive progeny in the SVZ (Fig 4F) suggested that the former progeny increasingly consisted of newborn bRG. As bRG are known to delaminate from the ventricular surface [24–27,31,35], we explored whether the radial glia progeny in the VZ observed upon conditional Pax6 expression increasingly showed signs of delamination. To this end, we measured the distance of the ventricular-most nucleus of each Tbr2–/Tbr2– daughter cell pair from the ventricular surface (Fig 4I). In light of the observation that the mean distance of the ventricular-most nucleus of the control and exoPax6-expressing Tbr2+/Tbr2– and Tbr2+/Tbr2+ daughter cell pairs was always >40 μm (S8 Fig), whereas that of the Tbr2–/Tbr2– pairs was <26.5 μm (Fig 4I, S8 Fig), we focused our attention on the abundance of the ventricular-most nuclei of Tbr2–/Tbr2– daughter cell pairs with a distance from the ventricular surface of ≥27 μm (corresponding to >3 nuclear diameters and referred to as abventricular location [16]). Whereas only 1 of the 7 ventricular-most nuclei (14%) of the Tbr2–/Tbr2– daughter cell pairs in the control was found in an abventricular location, 7 of the 15 nuclei (47%) analyzed upon conditional Pax6 expression were abventricular (Fig 4I). This suggested that conditional Pax6 expression promoted a substantial proportion of the radial glia progeny derived from neurogenic AP divisions to delaminate from the ventricular surface, as would be expected for newborn bRG.

In species with a high abundance of bRG in the SVZ, the radial thickness of the VZ decreases concomitant with bRG generation [8,23,25–27,64]. In light of the findings described above, we investigated a possible reduction in VZ thickness upon conditional Pax6 expression by quantifying the total number of nuclei (both RFP–and RFP+) in the VZ within a 200-μm wide, electroporated region of the dorsolateral telencephalon. Indeed, we observed a significant, approximately 10%, reduction in the number of nuclei in the VZ upon conditional Pax6 expression (Fig 4J). The magnitude of this reduction was consistent with the efficiency of electroporation and the estimated increase in the proportion of the progeny of electroporated neurogenic APs that delaminated upon conditional Pax6 expression as compared to control (Fig 4H). Taken together, the findings presented so far strongly suggest that mouse neurogenic APs and their progeny that constitutively express Pax6 increasingly generate bRG at the expense of generating bIPs.

### Conditional Pax6 Expression Increases bRG

**Fig 5:**
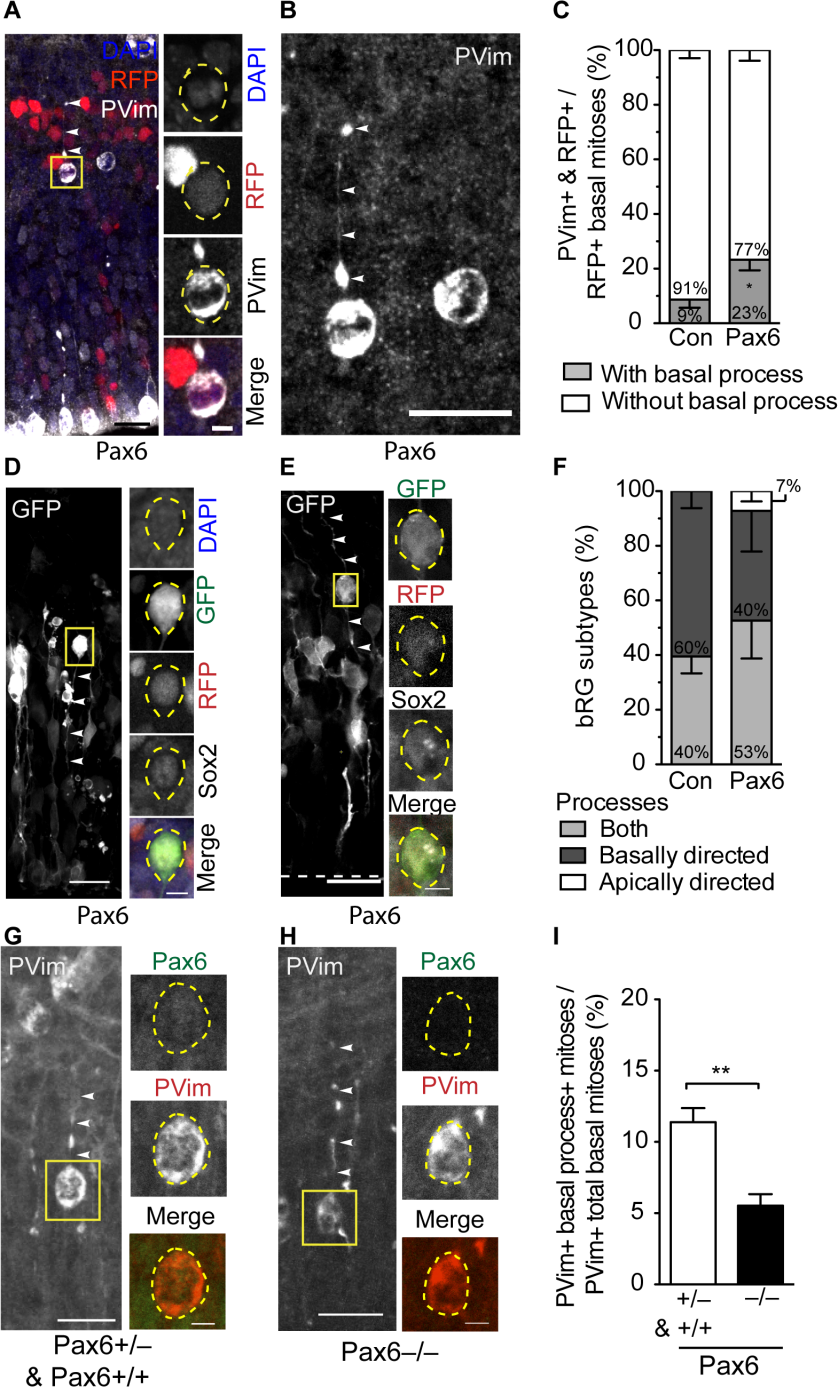
Conditional Pax6 expression in Tis21-positive APs increases bRG. (**A**–**F**) Dorsolateral telencephalon of tamoxifen-treated E14.5 *Tis21*–CreER^T2^ heterozygous mice electroporated at E13.5 with control (**C,F)** or Pax6-expressing (**A**–**F**) plasmid (see Fig 2B). (**A,B**) Phosphovimentin (PVim, white) immunofluorescene and RFP fluorescence (red), combined with DAPI staining (blue), on coronal 50-μm vibratome sections. Note the mitotic APs at the bottom of the image. PVim-positive BPs in the SVZ are shown at higher magnification in (**B**). Yellow box indicates the cell body (yellow dashed lines) that is shown as single optical sections at higher magnification in the small panels in (**A**). White arrowheads, basal process. Scale bars, 20 μm and 5 μm (small panels). (**C**) Quantification of mitotic BPs with (grey) or without (white) a basal process upon control and Pax6 electroporation. Mean of three independent experiments, each being the average of two to three embryos; control, 99 cells; Pax6, 77 cells. Error bars, SEM. * *p* < 0.05. (**D**–**F**) bRG exist in different morphologies in developing mouse neocortex. (**D,E**) Examples of bRG, derived from Pax6-electroporated cells, with an apically directed process (**D**) and both an apically and a basally directed process (**E**), as revealed by GFP fluorescence (maximum intensity projection of stacks of nine images **(D),** single optical section (**E**)). Yellow boxes indicate the cell body (yellow dashed lines) that is shown as single optical sections at higher magnification in the small panels; Sox2 immunofluorescence (white), GFP (green) and RFP (red) fluorescence, combined with DAPI staining (**D**) on coronal 25-μm cryosections. White arrowheads, apically and basally directed process; dashed white lines, ventricular surface. Scale bars; 20 μm and 5 μm (small panels). (**F**) Quantification of both process-bearing bRG (light grey), basal process-bearing bRG (dark grey), and apically directed process-bearing bRG (white), in control and upon conditional Pax6 expression. Mean of three independent experiments, each being the average of two to three embryos; control, 19 cells; Pax6, 25 cells. Error bars, SEM. (**G**–**I**) Dorsolateral telencephalon of E14.5 embryos containing no *Pax6* (–/–) or at least one copy of *Pax6* (+/+ and +/-), obtained by crossing heterozygous *Sey* mice. (**G,H**) Examples of bRG that contain at least one copy of functional *Pax6* (**G**) or none (**H**); phosphovimentin (white) immunofluorescence (maximum intensity projection of five (left) and six (right) images, respectively). Yellow boxes indicate the cell body (yellow dashed lines) that is shown as single optical sections at higher magnification in the small panels; Pax6 (green) and phosphovimentin (red) immunofluorescence, combined with DAPI staining on coronal 50-μm vibratome sections. Scale bars, 20 μm and 5 μm (small panels). White arrowheads, basal process. (**I**) Quantification of process-bearing basal mitotic cells in littermates containing at least one (+/+ and +/–, white) or no (–/–, black) copy of functional *Pax6*. Mean of five to six embryos from two different litters. Error bars, SEM. ** *p* < 0.01.

To corroborate and complement these findings, we next investigated the effect of conditional Pax6 expression on the proportion of bRG in the BP progeny of electroporated aRG. To this end, we analyzed the morphology of mitotic BPs using phosphovimentin immunofluorescence (Fig 5A–5C), which stains both the cell bodies and processes of mitotic cortical progenitors [66]. bRG characteristically extend basally and/or apically directed processes [23–29,31,35], whereas bIPs do not [17,18,21–26,28,35]. As the apically directed processes have been reported to be thinner than basal processes and may not be easily detected via phosphovimentin staining [23], we focused our analysis on basal process-bearing mitotic BPs. In the control, the vast majority (91%) of mitotic BPs were nonpolar and only a small minority (9%) extended a basal process (Fig 5C), consistent with the high abundance of bIPs and low abundance of bRG in the embryonic mouse SVZ [28,29,35]. In contrast, upon conditional Pax6 expression, we observed a more than 2-fold increase in the proportion of mitotic BPs with a basal process, i.e. of bRG within the BP population (23%, Fig 5C). These data show that, concomitant with the increase in the proportion of BPs among the aRG progeny (Fig 2K), conditional Pax6 expression more than doubled the proportion of bRG within these BPs.

As the apically-directed process of bRGs may be harder to detect via phosphovimentin immunofluorescence at mitosis [23], we next investigated the diversity of bRG morphology during interphase. To do this, we made use of the residual membrane-GFP (Fig 2A) expressed presumably due to incomplete Cre recombination (see Materials and Methods, live imaging) (Fig 5D and 5E). To distinguish bRG from migrating neurons, we stained for Sox2, which is expressed in radial glia but not in neurons. In the control, all of the bRG progeny of the electroporated neurogenic APs exhibited a basal process, and 40% of them an apically-directed process as well (Fig 5F). Upon conditional Pax6 expression, we found an increase in the proportion of bRG exhibiting both basally and apically directed processes (Fig 5E and 5F, 53%) and also observed bRG with an apically directed process only (Fig 5D and 5F, 7%). Interestingly, in the macaque, bRG with both processes and bRG with an apically directed process only have been reported to have a higher self-renewing capacity as compared to bRG with a basal process only [23]. Of note, the basal process of the bRG generated upon conditional Pax6 expression sometimes extended all the way to the pia (S9A Fig).

The bRG generated upon conditional Pax6 expression were nestin-positive (S9B Fig), could be Tbr2-negative (S9C Fig), and typically exhibited a perinuclear centrosome (S9D Fig). Furthermore, these cells underwent mitotic somal translocation, in which the cell soma moves rapidly in the basal or apical direction prior to mitosis [23,26,28,31], as revealed by live time-lapse imaging (S9E Fig).

### Lack of Pax6 Decreases bRG Abundance

The data presented so far show increased bRG generation upon elevating Pax6 levels in neurogenic aRG and sustaining it in the BPs derived therefrom. We sought to complement these findings by a converse, loss-of-function, approach. To this end, we investigated the proportion of mitotic (phosphovimentin-positive) bRG among BPs in the dorsolateral telencephalon of E14.5 homozygous *small eye* (*Sey*) mutant mice, which lack functional Pax6 because of a mutation that generates a premature translational stop codon (Fig 5G–5I). We found a significant reduction in the percentage of bRG as compared to littermates that have at least one copy of the *Pax6* gene (Fig 5I). These data indicate that although Pax6 function is not absolutely required for bRG generation, its level of expression is crucial for determining the abundance of these cells in the developing mouse neocortex.

### Conditional Pax6 Expression Increases Cell Cycle Re-entry of BPs

**Fig 6:**
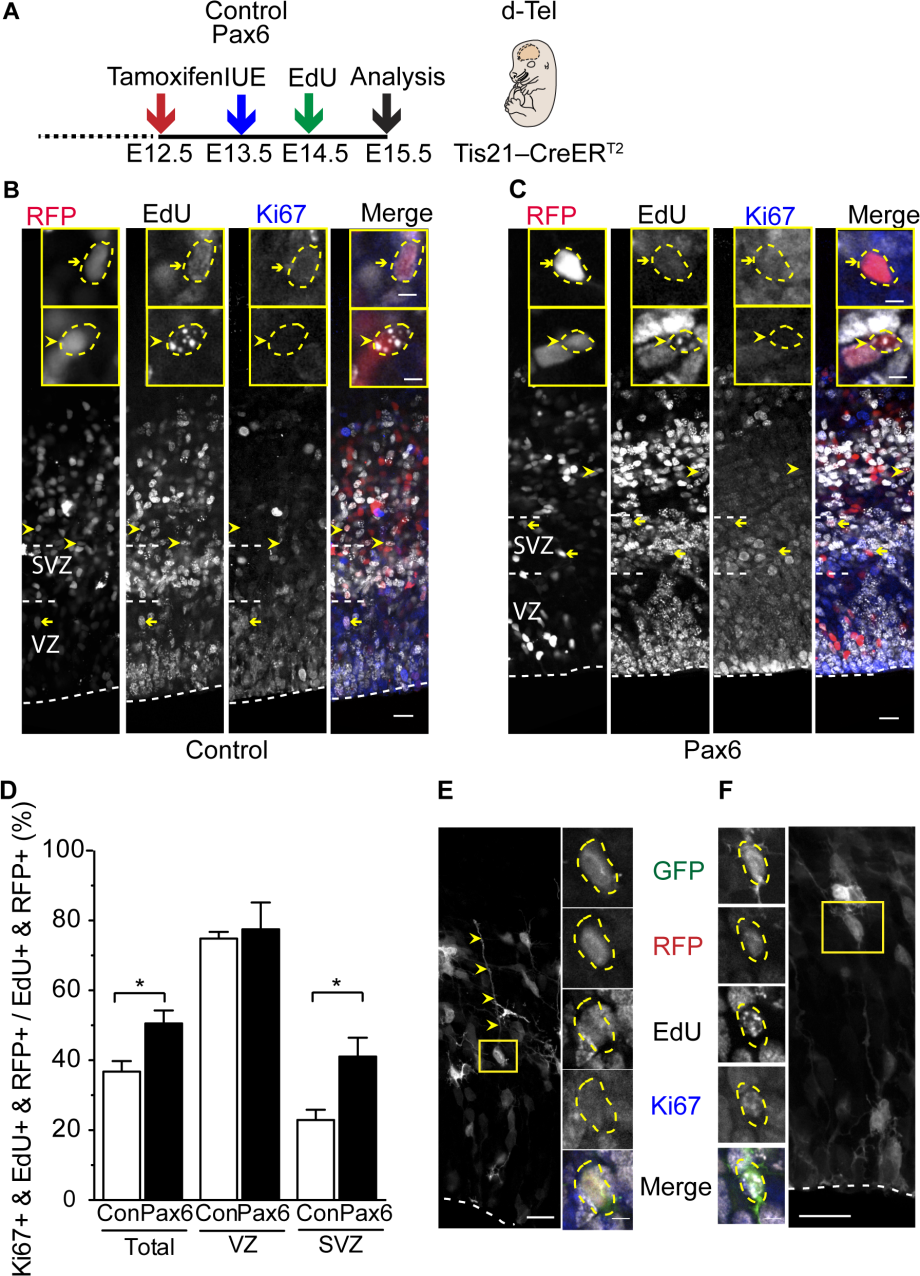
Conditional Pax6 expression in Tis21-positive APs increases BPs that re-enter the cell cycle. (**A**) Flow scheme of experiment. (**B–F**) Dorsolateral telencephalon of tamoxifen-treated E15.5 *Tis21*–CreER^T2^ heterozygous mice electroporated at E13.5 with control (**B,D**) or Pax6-expressing (**C**–**F**) plasmid and subjected to a single EdU pulse at E14.5. (**B,C**) Ki67 immunofluorescence (blue) and EdU (white) and RFP (red) fluorescence on coronal 50-μm vibratome sections. Yellow arrows, triple-positive cells (RFP+, EdU+, Ki67+); yellow arrowheads, double-positive cells (RFP+, EdU+, Ki67–). Insets show representative examples of RFP- and EdU-positive nuclei (outlined by dashed yellow lines) at higher magnification that are either Ki67-positive (yellow arrows, top) or-negative (yellow arrowheads, bottom). (**D**) Quantification of Ki67+, EdU+, and RFP+ triple-positive cells in the cortical wall (total), VZ, and SVZ, expressed as percentage of all cells that are both EdU+ and RFP+ in the cortical wall (200-μm wide area), upon control (Con, white) and Pax6 (black) electroporation. Mean of three independent experiments, each being the average of three embryos. Error bars, SEM. * *p* <0.05. (**E**,**F**) Examples of BPs, derived from Pax6-electroporated cells, with (**E**) and without (**F**) a basal process as revealed by GFP fluorescence (maximum intensity projection of stacks of 15 **(E)** and 8 **(F)** images, respectively). Yellow arrowheads, basal process; yellow boxes indicate the cell body (yellow dashed lines) that is shown as single optical sections at higher magnification in the small panels; EdU (white) and Ki67 (blue) double immunofluorescence and GFP (green) and RFP (red) fluorescence. (**B**,**C**,**E**,**F**) White dashed lines, ventricular surface; scale bars, 20 μm (**B**,**C**) and 5 μm (insets in **B**,**C** and **E**,**F**).

Ferret and primate bRG are known to undergo multiple rounds of self-renewing division [23–26,31], whereas bIPs in mouse and rat embryonic neocortex typically undergo one round of self-consuming division [16–18,20–22]. In light of the increase in cycling BPs (Fig 2K) and bRG (Fig 5C) upon conditional Pax6 expression, it was therefore of interest to investigate whether conditional Pax6 expression would subsequently lead to increased cell cycle re-entry of the BP progeny derived from electroporated aRG. To this end, a single pulse of EdU was administered at 24 h after electroporation and analyzed after an additional 24 h for the proportion of cycling, Ki67-positive cells among the EdU-labeled progeny of electroporated APs, in order to identify cells that had re-entered the cell cycle (Fig 6A).

In the control, 75% of such daughter cells present in the VZ, but only 23% of such daughter cells in the SVZ, had re-entered the cell cycle (Fig 6B and 6D). In contrast, upon conditional Pax6 expression, whereas daughter cell cycle re-entry was the same in the VZ, it nearly doubled in the SVZ (41%, Fig 6C and 6D). Again, we used the residual membrane-GFP fluorescence to determine the morphology of daughter cells that had re-entered the cell cycle. Two types of such daughter cells were observed, monopolar cells with a distinct basal process (Fig 6E), i.e., bRG, and multipolar cells with short extensions during interphase (Fig 6F), presumably bIPs.

### Live Imaging of the Fate of the Progeny Arising from Divisions of bRG

**Fig 7:**
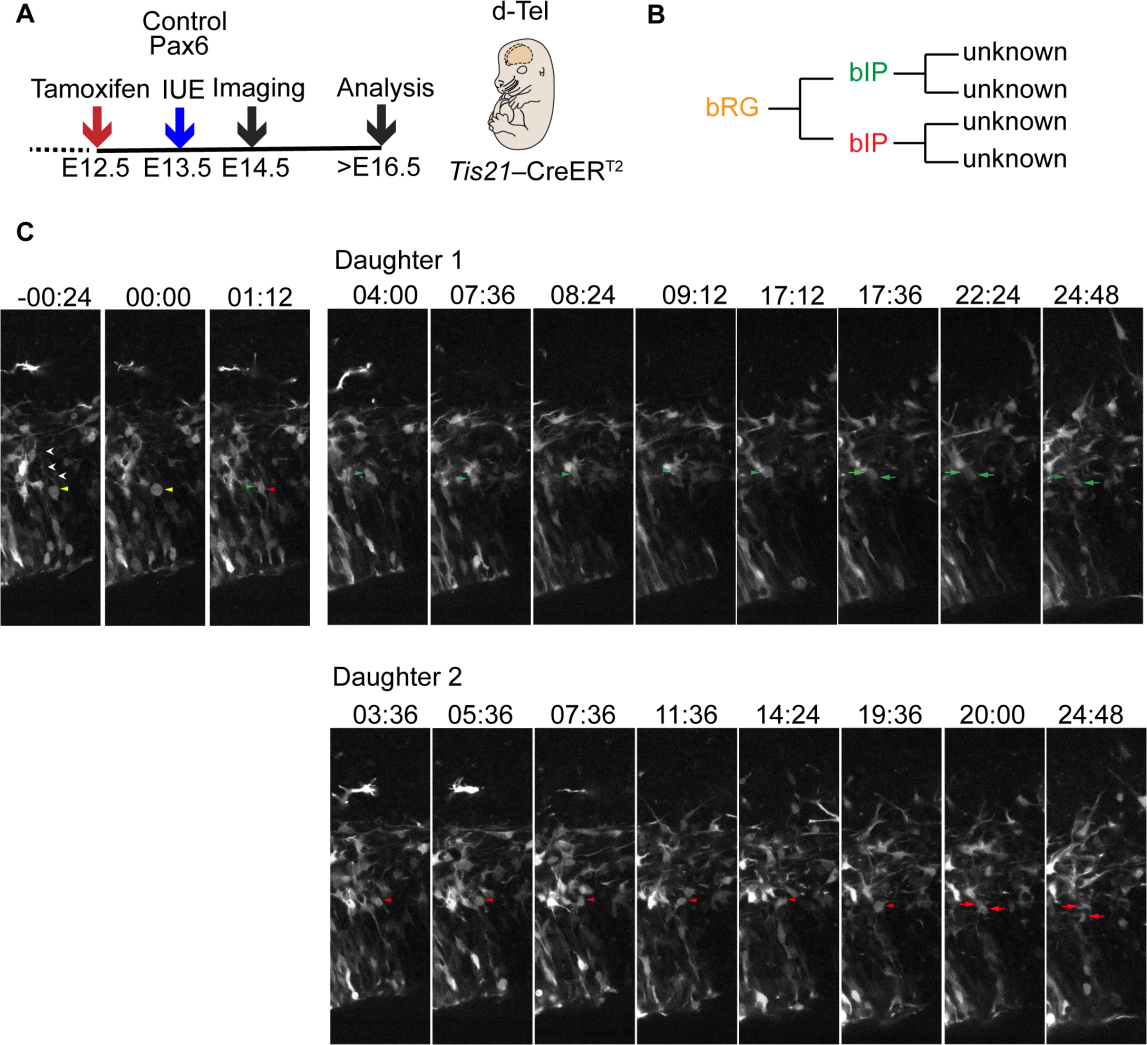
Time-lapse imaging of bRG generated upon conditional Pax6 expression and its progeny—symmetric proliferative division. (**A**) Flow scheme of experiment. (**B**) Lineage tree reconstruction of bRG division. (**C**) Live time-lapse imaging of organotypic slice of dorsolateral telencephalon of tamoxifen-treated E14.5 *Tis21*–CreER^T2^ heterozygous mice electroporated at E13.5 with Pax6-expressing plasmid. Membrane-GFP fluorescence, single optical sections. 00:00 (hh:mm) denotes the start of mitosis. Yellow arrowheads, mother bRG; white arrowheads, basal process; green and red arrowheads, bIP daughter 1 and bIP daughter 2, respectively, of mother bRG; green and red arrows, daughter cells of bIP daughter 1 and bIP daughter 2, respectively.

We extended these data by analyzing the fate of the progeny derived from divisions of bRG, using live time-lapse imaging for at least 48 h of organotypic slices prepared from control and Pax6-expressing plasmid-electroporated E14.5 neocortex (Fig 7A). Despite the rare occurrence of bRG in mouse neocortex, we were able to identify several RFP-positive bRG, to image their divisions, and to track their progeny for at least an additional 20 h (i.e., for a time period longer than the average Tc of self-renewing bRG (see S2 Table)). In the control, in two out of the seven cases analyzed, the mitotic bRG underwent an asymmetric self-renewing division, as one of the daughter cells was observed to re-enter the cell cycle (S10 Fig). In the other five cases, similar to what has been previously reported for the embryonic mouse brain [28], both daughters did not enter mitosis during the time of our observations (S10 Fig).

Upon conditional Pax6 expression, half of the mitotic exoPax6-expressing bRG (three out of six) gave rise to progeny that subsequently underwent another round of cell division (S10 Fig). In two cases, these bRG divisions were asymmetric self-renewing (S10 and S11 Figs, S1 Movie). Remarkably, we also observed a bRG undergoing a symmetric proliferative division (Fig 7, S10 Fig, S2 Movie), with both daughters undergoing another round of cell division. These live imaging data are consistent with the notion that bRG generated upon conditional Pax6 expression and their progeny are endowed with greater proliferative potential as compared to control. Moreover, together with the cell cycle re-entry analysis (Fig 6), these data suggest that BPs show an increased proliferative capacity upon conditional Pax6 expression.

### Conditional Pax6 Expression Increases Oblique Cleavage Plane Orientation of Neurogenic APs

**Fig 8:**
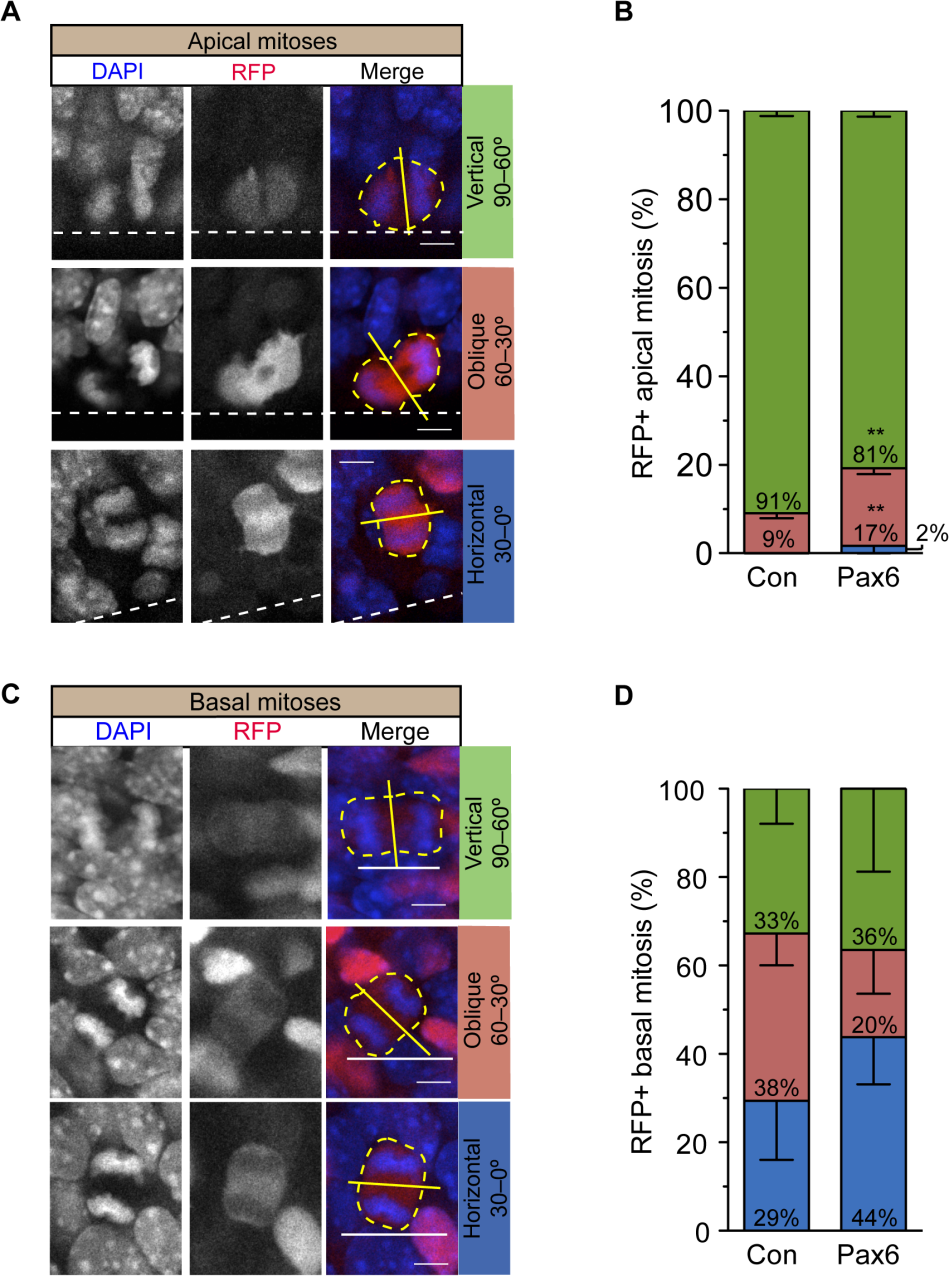
Conditional Pax6 expression in Tis21-positive cortical progenitors increases nonvertical cleavage plane orientation. Dorsolateral telencephalon of tamoxifen-treated E14.5 *Tis21*–CreER^T2^ heterozygous mice electroporated at E13.5 with control (**B,D**) or Pax6-expressing (**A–D**) plasmid (see Fig 2B). (**A**) Examples of apical mitoses with vertical (top), oblique (middle) or horizontal (bottom) cleavage plane orientation (yellow solid lines) relative to the ventricular surface (white dashed lines). RFP fluorescence (red), combined with DAPI staining (blue), on coronal 50-μm vibratome sections. (**B**) Quantification of cleavage plane orientation of apical mitoses relative to the ventricular surface upon control and Pax6 electroporation; green, cleavage plane orientation 90–60° (vertical); red, cleavage plane orientation 60–30° (oblique); blue, cleavage plane orientation 30–0° (horizontal). Control, 34 mitoses; Pax6, 45 mitoses. Error bars, SEM. ** *p* <0.01. (**C**) Examples of basal mitoses in the SVZ with vertical (top), oblique (middle), and horizontal (bottom) cleavage plane orientation (yellow solid lines) relative to the ventricular surface (not contained in the images but represented by the white solid lines). RFP fluorescence (red), combined with DAPI staining (blue), on coronal 50-μm vibratome sections. (**D**) Quantification of cleavage plane orientation of basal mitoses of progenitors derived from APs relative to the ventricular surface, upon control and Pax6 electroporation; green, cleavage plane orientation 90–60° (vertical); red, cleavage plane orientation 60–30° (oblique); blue, cleavage plane orientation 30–0° (horizontal). Control, 35 mitoses; Pax6, 34 mitoses. Error bars, SEM. (**A**,**C**) Yellow dashed lines, outline of cell body; scale bars, 5 μm.

It has been reported that a nonvertical (i.e., oblique or horizontal) cleavage plane orientation in relation to the ventricular surface of dividing APs (for examples, see Fig 8A) increases the probability that daughter cells become bRG [24,31,35,64]. We investigated whether the increased generation of bRG upon conditional Pax6 expression involved such alterations in cleavage plane orientation. In the control, the vast majority (91%) of mitotic neurogenic APs showed a vertical, and only a small minority (9%) an oblique, cleavage plane orientation (Fig 8B), consistent with previous data on Tis21-expressing APs [21,67,68]. Strikingly, conditional Pax6 expression resulted in a significant increase in nonvertical cleavage planes in mitotic neurogenic APs (19%, Fig 8B). As this doubling matched the doubling in the proportion of bRG among BPs (Fig 5C), our observations suggest that the increase in nonvertical cleavage plane orientations of neurogenic APs upon conditional Pax6 expression (Fig 8B) causally contributed to the increased generation of bRG (Fig 4F and 4H, Fig 5C).

### Conditional Pax6 Expression Increases Horizontal Cleavage Plane Orientation of BPs

The doubling in cell cycle re-entry of BPs upon conditional Pax6 expression (Fig 6D) matched the doubling of bRG (Fig 5C), which in primates are endowed with constitutive cell cycle re-entry capacity [23,24,26]. However, the morphology of the BPs that had re-entered the cell cycle (Fig 6E and 6F, Fig 7) raised the possibility that the increased cell cycle re-entry of BPs upon conditional Pax6 expression (Fig 6D) may not only be due to the increase in the proportion of bRG (Fig 5C) but may in addition reflect an increased cell cycle re-entry of bIPs (Fig 6F, Fig 7). Moreover, inducing mouse Tbr2-positive BPs to re-enter the cell cycle by forced premature expression of the transcription factor Insm1 has been shown to be associated with an alteration in their cleavage planes from the normal near-random [21,24,63] to mostly horizontal orientations [63]. Finally, not only human bRG are thought to divide preferentially with a near-horizontal cleavage plane [24] but also Tbr2-positive progenitors in the human SVZ, which, like their macaque counterpart [23] and in contrast to mouse bIPs, are endowed with proliferative capacity [26] and show a near-horizontal cleavage plane orientation in the majority of cases [25]. These considerations prompted us to investigate whether conditional Pax6 expression, concomitant with increasing the cell cycle re-entry of the mouse BPs derived from electroporated aRG, would perhaps increase the proportion of horizontal cleavages of these BPs (for examples of vertical, oblique, and horizontal BP cleavage planes in relation to the ventricular surface, see Fig 8C).

In the control, the BP progeny derived from neurogenic aRG showed a random cleavage plane orientation at mitosis (Fig 8D), consistent with previously published data [21,63]. Interestingly, conditional Pax6 expression caused an increase (albeit not statistically significant) in the proportion of the BP progeny that divided with a horizontal cleavage plane, decreasing the proportion of oblique cleavage planes (Fig 8D). Given that conditional Pax6 expression increased not only the proportion of bRG among the BP progeny derived from electroporated neurogenic aRG (Fig 5C) but also the proliferative capacity of this progeny in general (Fig 2K, Fig 6D), our cleavage plane data are consistent with the notion that a horizontal cleavage plane may be a hallmark of BPs endowed with self-renewal capacity, that is, bRG and proliferative bIPs [24,31,63].

### Conditional Pax6 Expression Increases Upper-Layer Neuron Generation at Midneurogenesis

**Fig 9:**
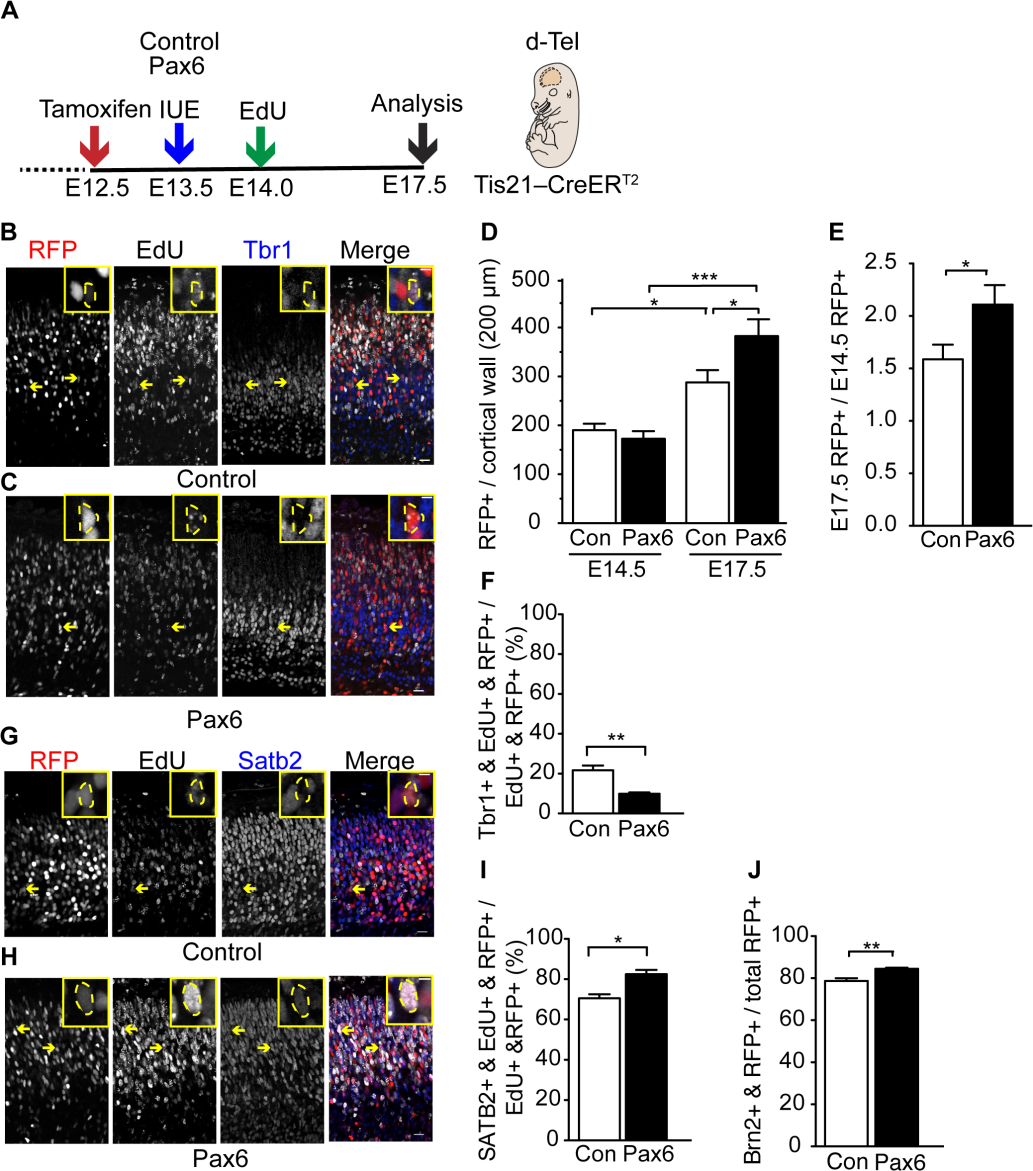
Conditional Pax6 expression in Tis21-positive cortical progenitors increases upper-layer neuron production. (**A**) Flow scheme of the experiment. (**B**–**J**) Dorsolateral telencephalon of tamoxifen-treated E14.5 (**D**,**E**) and E17.5 (**B**–**J**) *Tis21*–CreER^T2^ heterozygous mice electroporated at E13.5 with control (**B,D–F,G,I,J**) or Pax6-expressing (**C**–**F,H–J**) plasmid and subjected to a single EdU pulse at 10 h after electroporation (E14.0). (**B,C**,**G,H**) Tbr1 (**B,C**) and Satb2 (**G**,**H**) immunofluorescence (blue) and EdU (white) and RFP (red) fluorescence in the cortical plate (coronal 50-μm vibratome sections). Insets are representative examples of RFP-positive nuclei (outlined by dashed yellow lines) at higher magnification that are either Tbr1- or Satb2-positive. Scale bars, 20 μm (**B**,**C**,**G**,**H**) and 5 μm (insets in **B**,**C,G,H**). (**D,E**) Quantification of RFP+ nuclei in the entire cortical wall (200-μm wide area) (**D**) and their fold-increase at E17.5 as compared to E14.5 (**E**), upon control (Con, white) and Pax6 (black) electroporation. (**D**) E14.5, mean of three independent experiments, each being the average of two or three embryos (eight in total); E17.5, mean of eight embryos from at least two independent experiments; (**E**) mean of eight E17.5/E14.5 ratios. (**F**,**I,J**) Quantification of Tbr1+, EdU+ and RFP+ triple-positive nuclei (**F**), Satb2+, EdU+ and RFP+ triple-positive nuclei (**I**) and Brn2+ and RFP+ double-positive nuclei (**J**) in the cortical plate, expressed as percentage of all cells that are EdU+ and RFP+ (**F**,**I**) and RFP+ (**J**) in the cortical plate (200-μm wide area) upon control (Con, white) and Pax6 (black) electroporation. (**F**,**I**) Mean of three independent experiments, each being the average of two to three embryos. (**J**) Mean of four embryos from two different litters. Error bars, SEM. * *p* < 0.05, ** *p* < 0.01, *** *p* < 0.001.

As conditional Pax6 expression increased the proliferative capacity of the BP progeny of neurogenic aRG and the proportion of bRG among these BPs, we finally investigated the consequences for cortical neurogenesis. To this end, we administered EdU 10 h after electroporation, at ≈E14.0, i.e., around the start of exo-Pax6 expression, in order to label the neuronal progeny born at this midneurogenesis stage, followed by their analysis in the cortical wall at E17.5 (Fig 9A).

We first quantified the population size of the total progeny at E17.5. Compared to E14.5, this progeny population size was increased in both the control (1.6-fold) and upon conditional Pax6 expression (2.1-fold), with the latter increase being significantly greater than the former (Fig 9D and 9E). This indicated that conditional Pax6 expression increased the total cell output observed at E17.5.

Of note, in the control, the majority (68%) of the progeny had migrated to the cortical plate (S12A and S12D Fig). In contrast, in the case of conditional Pax6 expression, this was observed for only approximately one third (31%) of the progeny, the majority of which exhibited heterotopia in the intermediate zone (S12B and S12D Fig). Strikingly, most of the heterotopia cells had a much higher level of Pax6 immunoreactivity than those that had reached the cortical plate (S12C and S12E Fig). These observations are consistent with previous findings in Pax6-overexpressing mouse models, in which aggregates of Pax6-overexpressing cells in the developing cortical wall have been described [58]. Further characterization of the progeny exhibiting heterotopia showed that these cells were immature neurons (S13 Fig).

Next, we analyzed the neuronal fate of the progeny that had migrated to the cortical plate. To distinguish between deep-layer and upper-layer neurons, we made use of established markers, the transcription factor Tbr1, which labels layer V and VI neurons, and the transcriptional regulators Satb2 and Brn2, which label layer II–IV neurons [69]. Conditional Pax6 expression reduced the proportion of EdU-labeled Tbr1-positive neurons originating from the electroporated neurogenic aRG (Fig 9B, 9C and 9F). Conversely, the proportion of EdU-labeled neurons that expressed Satb2 was increased (Fig 9G–9I). Similarly, the percentage of Brn2-positive cells among the neuronal progeny was significantly increased upon conditional Pax6 expression (Fig 9J). Together with the overall increase in progeny observed at E17.5 (Fig 9D and 9E), these data show that conditional Pax6 expression at midneurogenesis increases the generation of upper-layer neurons. This likely reflected the increase in BP proliferative capacity (Fig 2K, Fig 6D, Fig 7, S10 Fig) and relative bRG abundance (Fig 4F, Fig 5C) upon conditional Pax6 expression.

### Increased Upper-Layer Neuron Production and Occurrence of Progenitors Residing in the Intermediate Zone of Neocortex of Transgenic Mouse Embryos Sustaining Pax6 Expression in the Neurogenic Lineage

**Fig 10:**
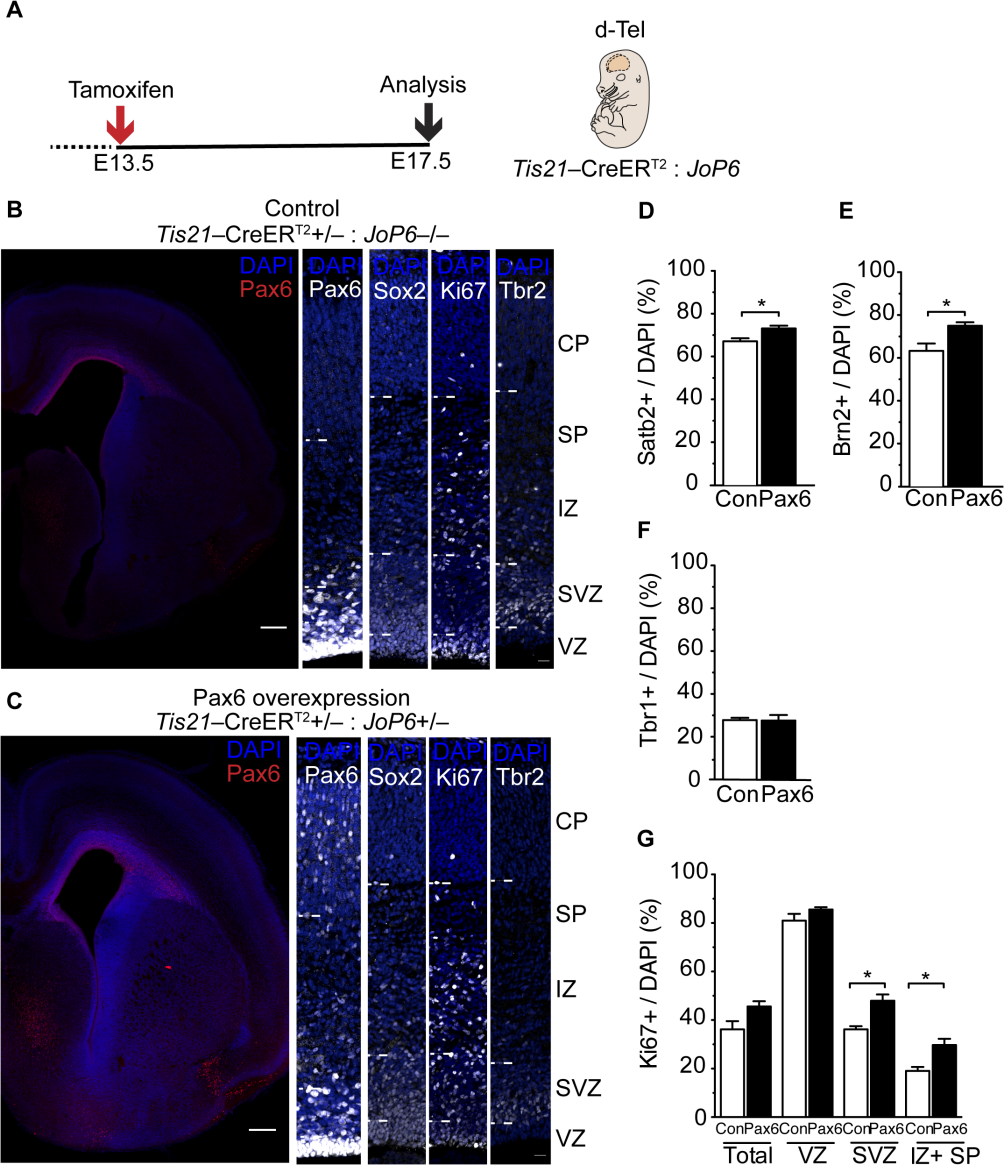
Double-transgenic mouse embryos sustaining Pax6 expression show increased BP abundance and upper-layer neuron production. (**A**) Flow scheme of experiment. (**B**–**G**) Dorsolateral telencephalon of tamoxifen-treated E17.5 *Tis21*–CreER^T2^: *JoP6* double-transgenic mice; control (*Tis21*–CreER^T2^+/–: *JoP6*–/–) (**B**) and Pax6 overexpression (*Tis21*–CreER^T2^+/–: *JoP6*+/–) (**C**) littermates. (**B**,**C**) Representative images showing Pax6 (red and white), Sox2 (white), Ki67 (white), or Tbr2 (white) immunofluorescence, combined with DAPI staining (blue), on coronal 50-μm vibratome sections. Scale bars, 20 μm. Note the lack of heterotopia formation in the Pax6 overexpression condition in the Pax6 overview image in (**C**). (**D–F**) Quantification of Satb2+ (**D**), Brn2+ (**E**) and Tbr1+ (**F**) nuclei in the cortical plate (100-μm wide area), in control (Con, white) and Pax6 overexpression (black) littermates. (**G**) Quantification of Ki67+ nuclei in the cortical wall except for the cortical plate (total), VZ, SVZ, and intermediate zone plus subplate (IZ + SP) (100-μm wide area), in control (Con, white) and Pax6 overexpression (black) littermates. (**D–G**) Mean of three to six embryos from two different litters. Error bars, SEM. * *p* < 0.05.

We complemented and extended the data obtained by the conditional Pax6 expression using in utero electroporation by taking a double-transgenic approach. Specifically, we crossed the *Tis21–*CreER^T2^ mice with *JoP6* mice [58]. Like the Pax6-expressing plasmid, *JoP6* mice contain, under a constitutive promoter, a floxed GFP-stop cassette followed by *Pax6*, an IRES sequence and a reporter [58]. Upon Cre recombination induced by tamoxifen administration at E13.5 (Fig 10A), Pax6 will be expressed at elevated levels in neurogenic aRG, and this expression sustained in their progeny throughout the embryonic neocortex.

With this approach, similar to the results obtained upon the conditional Pax6 expression via in utero electroporation of *Tis21–*CreER^T2^ mouse embryos (Fig 9), upper-layer neurons as identified by Satb2 and Brn2 expression were significantly increased when compared to control littermates (Fig 10D and 10E). By contrast, deep-layer neurons as identified by Tbr1 expression were not affected (Fig 10F). Strikingly, with this double-transgenic approach (Fig 10A), we did not observe the heterotopia seen upon conditional Pax6 expression using in utero electroporation (S12B Fig). This may reflect the more standardized way in which elevated and sustained Pax6 expression is achieved in the double-transgenic embryos.

In ferret and primate neocortex, the increase in proliferating BPs is accompanied by an expansion of the SVZ in the basal direction, that is, an increase in BPs residing in the oSVZ, the key basal-most germinal layer characterized by lesser cell density [4–6,8,32]. To explore whether the *Tis21–*CreER^T2^: *JoP6* double-transgenic approach resulted in an increase in BPs residing in cortical low-cell-density layers basal to the mouse SVZ proper, that is, the intermediate zone and subplate, we examined the distribution of the cell proliferation marker Ki67. Whereas there was no significant difference in the abundance of Ki67-positive cells between control (*Tis21–*CreER^T2^+/–: *JoP6*–/–) and Pax6-overexpressing (*Tis21–*CreER^T2^+/–: *JoP6*+/–) neocortex in the VZ, we observed a significant increase in Ki67-positive cells not only in the SVZ, but also in the intermediate zone and subplate of Pax6-overexpressing mouse neocortex (Fig 10B, 10C and 10G). It is therefore interesting to note that the increase in progenitors residing in cortical low-cell-density layers basal to the SVZ of embryonic mouse neocortex observed with the *Tis21–*CreER^T2^: *JoP6* double-transgenic approach of Pax6 overexpression is reminiscent of one of the features of the ferret and primate oSVZ.

## Discussion

### A Novel Approach to Conditionally Express Pax6 Selectively in BP-Genic aRG and Their Progeny

**Fig 11:**
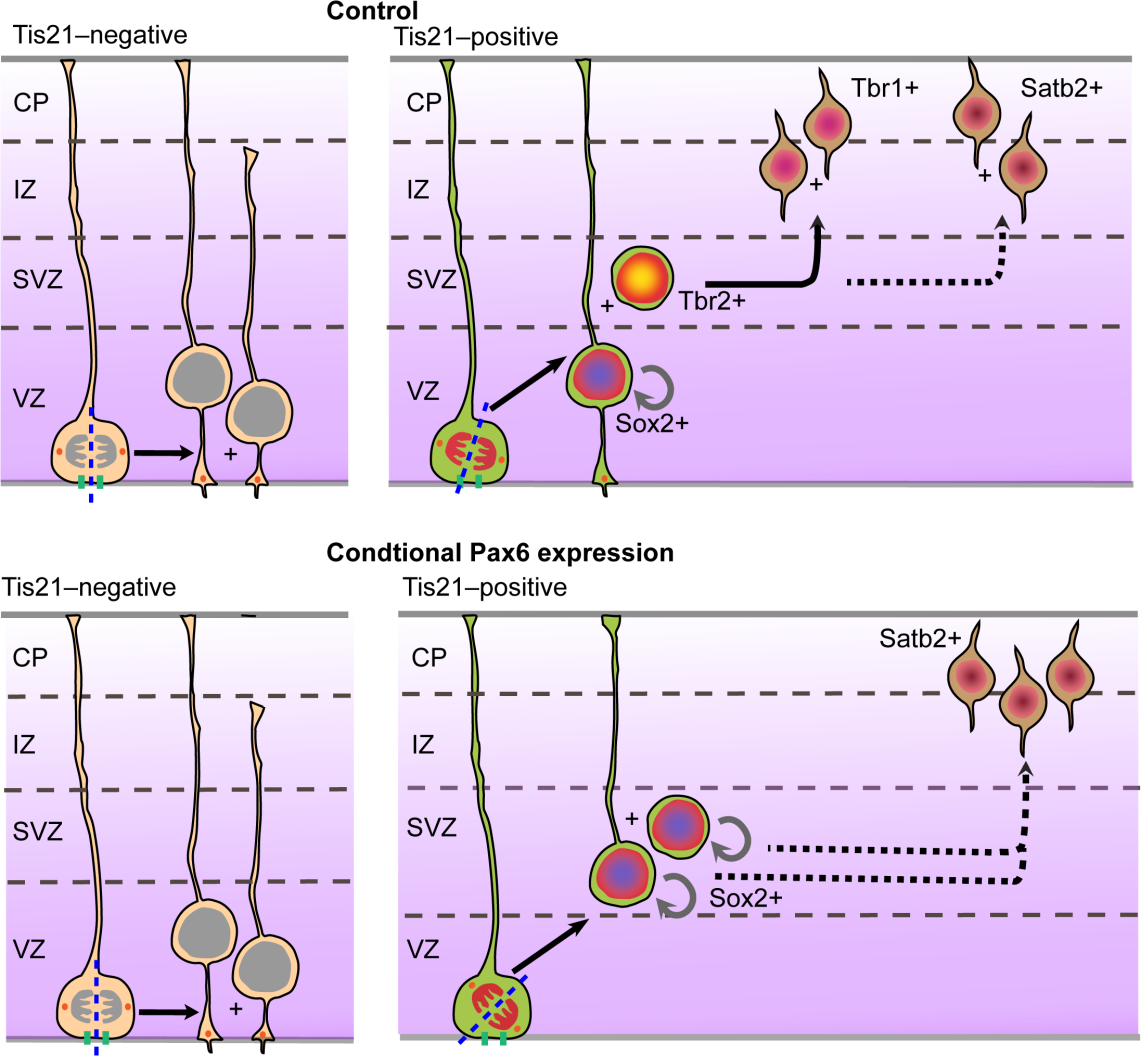
Model. Cartoon showing the effects of conditional Pax6 expression on Tis21-positive APs.

BPs endowed with proliferative capacity, notably bRG, are a hallmark of the developing primate neocortex [1–6,8,23,34,70]. Here we show that a single transcription factor, Pax6, when specifically sustained in the aRG-to-BP lineage, is sufficient to generate such BPs (Fig 11). Our study differs in key aspects from previous studies in which Pax6 expression was increased in APs of dorsolateral telencephalon, as the latter either did not observe or address effects on BPs [48,58,71], or obtained opposite results [46]. Specifically, increased Pax6 expression was previously found to increase the mRNA levels for the bIP marker Tbr2 in the VZ and SVZ [46]. In contrast, in the present study, we found a decrease of Tbr2-positive BPs in the SVZ and nascent BPs in the VZ upon conditional Pax6 expression. Our observations are consistent with the increased generation of proliferative BPs, notably bRG. These differences in results presumably reflect the fact that in the previous study, Pax6 expression was increased in all APs, whereas in the present study, conditional Pax6 expression was confined to Tis21-positive, that is, neurogenic and BP-genic, APs and their progeny.

### Increasing Pax6 Expression in aRG Is Sufficient to Alter Their Cleavage Plane Orientation Towards Promoting bRG-Generating Divisions

Our findings have three significant implications for elucidating the evolutionary expansion of the neocortex. First, they reveal a key role of Pax6 in the generation of a primate-like SVZ, that is, of proliferative BPs from aRG. We find that the effects elicited by increased Pax6 levels on aRG mitosis and daughter cell fate in embryonic mouse neocortex reproduce the normal situation in fetal human neocortex, which is characterized by higher Pax6 levels in human than mouse aRG (S14 Fig). Specifically, increasing Pax6 levels in mouse BP-genic aRG increased their oblique cleavage plane orientation at the expense of vertical cleavage plane orientation (Fig 11), consistent with previous studies reporting a greater proportion of oblique and horizontal cleavages in human [24] than mouse [19,35,67,68,72,73] aRG. This alteration in cleavage plane orientation may well have been promoted by the fact that conditional Pax6 expression was selective for BP-genic aRG, which are more susceptible to spindle orientation variability due to the reduction of apical and basal astral microtubules as compared to proliferative aRG [67]. The increased oblique cleavage plane orientation of BP-genic aRG likely caused, in line with previous findings [35], the observed increase in (i) self-consuming bRG-genic divisions of mouse aRG at the expense of self-renewing bIP-genic divisions and (ii) aRG daughter cell delamination, and consequently (iii) the decrease in mouse VZ thickness. Taken together, our findings provide a mechanistic explanation for the reduction in VZ thickness that occurs concomitant with the growth of the oSVZ during the progression of cortical neurogenesis in species with an enlarged neocortex [8,23,25–27,32,64].

As to the mechanism how increased Pax6 levels in BP-genic aRG promote oblique cleavage plane orientation, previous work has identified an intriguing Pax6 target gene—the mitotic spindle—and kinetochore-associated protein *Spag5* [47]. An increase in nonvertical cleavage plane orientation of mouse aRG has been observed both upon knock-down of *Spag5* and when Spag5 mRNA and protein levels in *Pax6* mutant mice at midneurogenesis are elevated [47], suggesting that either too low or too high Spag5 levels perturb the normal, horizontal spindle orientation that is required for aRG vertical cleavage plane orientation. This is in line with the concept that for aRG, a horizontal spindle orientation is thought to reflect the active state of the mitotic spindle orientation machinery, and nonhorizontal spindle orientations can occur upon perturbation of this machinery [19,35,67,72,73].

By contrast, for mouse BPs, a default state of the mitotic spindle orientation machinery, with random cleavage plane orientations, is thought to be the normal situation [21,24,63], and activation of this machinery is thought to promote horizontal cleavage plane orientation. Such orientation prevails in primate BPs, notably bRG, which are endowed with much greater proliferative capacity than mouse BPs [23–26] and is increasingly observed when mouse BPs are induced to proliferate [63]. In this context, it is interesting to note that (i) the relative *Spag5* mRNA levels are much higher in the human iSVZ and oSVZ than the mouse SVZ [50], and (ii) increasing the Pax6 level in mouse BPs, which likely results in increased Spag5 levels, was found here to increase their horizontal cleavage plane orientation. Taken together, the concept emerges that Pax6, via its downstream targets including *Spag5*, increases oblique, self-consuming aRG divisions generating proliferative BPs, notably bRG, and horizontal BP divisions promoting their proliferation or self-renewal.

### Sustaining Pax6 Expression in BPs Is Sufficient to Promote Their Proliferation/Self-Renewal

Second, we observed that sustaining high Pax6 expression in BPs increases their cell cycle re-entry (Fig 6) and their abundance not only in the SVZ but even in the layers basal to the SVZ, the intermediate zone and subplate (Fig 10). This finding implies that the maintenance of expression of Pax6 in primate, but not mouse and rat, BPs, notably bRG, is a key feature of the machinery underlying their greater proliferative or self-renewal capacity [23–26]. It thus appears that Pax6 has the potential to promote proliferation and self-renewal of cortical progenitors in general, that is, for both APs [38,39,74] and BPs (this study). Conversely, as we observed a marked decrease in bRG in the dorsolateral telencephalon of *Sey* mouse embryos (Fig 5G–5I), it would be interesting to explore whether a similar decrease in bRG is observed in human embryonic stem cell-derived organoids [75] upon *PAX6* knockdown after establishment of the SVZ. As a corollary, the molecular mechanisms underlying the sustained Pax6 expression in BPs, at the level of mRNA and protein generation and stability [76–83], then become the crucial issue for SVZ enlargement and neocortex expansion.

The increased cell cycle re-entry of BPs observed here upon sustained Pax6 expression is in contrast to the previously reported increase in cell cycle exit of cortical progenitors in Pax6 overexpressing (PAX77) mice [48]. Again, this discrepancy presumably reflects the difference between conditional Pax6 overexpression selectively in BP-genic APs (present study) and constitutive Pax6 overexpression in all APs [48].

As to the downstream targets of Pax6 that promote BP proliferation or self-renewal, at least two candidates exist. One is the transcription factor Sox2, a well-known stimulator of stem and progenitor cell proliferation and self-renewal [38,59,84]. Pax6 has been shown to induce Sox2 expression [85], and consistent with this, we observed that sustaining Pax6 expression in the aRG–BP lineage indeed increases the proportion of Sox2-positive BPs. The other class of candidates are extracellular matrix (ECM) constituents and their receptors, the integrins, which have been implicated in BP proliferation and self-renewal [25,50,52,62,86]. Interestingly, Pax6 induces the expression of ECM constituents such as tenascin-C [87] and integrin α_5_β_1_ [88]. Hence, the increased cell cycle re-entry of BPs upon sustained Pax6 expression may well reflect, at least in part, an altered, more human-like, microenvironment in the mouse SVZ that is now more conducive to BP proliferation and self-renewal.

### Enhancing the Proliferative Capacity of BPs by Sustained Pax6 Expression Increases Upper-Layer Neuron Generation

Third, the increased proliferative capacity of mouse BPs achieved by sustained Pax6 expression resulted in a phenotypic change in the cortical plate that is characteristic of primates—an increase in upper-layer neurons (Figs 9 and 10). Also, this aspect of the present phenotype is in contrast to previous findings which showed, concomitant with increased progenitor cell cycle exit, an increase in deep-layer neurons at the expense of upper-layer neurons in the constitutively Pax6 overexpressing PAX77 mice [48]. It should be noted that conditional Pax6 expression in neurogenic aRG resulted in an increase in Pax6 levels that was substantially greater than that in human as compared to mouse APs (compare Fig 2G and S14 Fig). Moreover, upon the present approach of conditional expression, which used a constitutive promoter, Pax6 was found to be present even in neurons (S12 Fig). It is therefore comprehensible that the present approach of conditional Pax6 expression via in utero electroporation in embryonic mouse neocortex has phenotypic consequences, some of which are not observed in fetal human neocortex and upon more controlled Pax6 expression in the double-transgenic mouse (Fig 10), such as the heterotopia which consisted mostly of highly Pax6-positive immature neurons (S12 and S13 Figs).

Hence, considering all aspects of the present phenotype together, sustaining Pax6 expression in BP-genic aRG and the BPs derived therefrom, as is characteristically the case in fetal primate neocortex [23–27,50], is sufficient to induce primate-like progenitor behaviour in embryonic mouse neocortex, that is, (i) translocation of progenitors from the VZ to the SVZ, (ii) an increased proportion of bRG among the BPs generated, (iii) sustained proliferation or self-renewal of BPs in the SVZ, and (iv) an increased upper-layer neuron production. The differential regulation of Pax6 expression in cortical progenitors during development across mammals therefore emerges as a key issue of future studies aiming to understand the evolutionary expansion of the SVZ, and consequently the neocortex.

Although sustained Pax6 expression sufficed to generate primate-like bRG in developing mouse neocortex, it was insufficient to induce cortical folding (Figs 9 and 10). This is in contrast to previous studies in which the expression of specific genes implicated in neocortex expansion led not only to the expansion of BPs but also to folding of the mouse neocortex [89,90]. In these studies, the expansion of BPs comprised an increase in both bRG and bIPs. Expansion of bIPs alone has been reported to be insufficient to induce cortical folding in the mouse neocortex [91]. Moreover, the presence of bRG is essential for tangential dispersion of neurons [27] in order for the basal surface to expand more than the apical surface, and ultimately for cortical folding [4,5,8,27]. Hence, to increase the ratio of basal to apical surface, it appears to be critical to increase the proportion of bRG among the BPs in the SVZ above a certain level. This would increase the divergence of radial fibers emanating from the SVZ, allowing for a broader dispersion of migrating neurons. Our data suggest that a mere doubling of bRG abundance in the embryonic mouse neocortex (from 10% to 20% of all BPs), as was achieved by sustaining Pax6 expression, is still insufficient to result in cortical folding.

On a more general note, human-specific aspects of neocortex expansion can be considered to be caused by (i) the presence of a relevant gene in the human as well as nonhuman genomes, but with differential regulation of expression between human and nonhuman species [50,89,92]; (ii) the presence of a relevant gene in the human as well as nonhuman genomes, but with human-specific alterations in the coding sequence [93,94]; and (iii) the presence of a relevant gene in the human, but not nonhuman, genomes [90]. The present study demonstrates that Pax6, a central player in corticogenesis, can be regarded as a key example of the first scenario.

## Materials and Methods

### Ethics

Human fetal brain tissue was obtained from the Klinik und Poliklinik für Frauenheilkunde und Geburtshilfe, Universitätsklinikum Carl Gustav Carus of the Technische Universität Dresden, following elective pregnancy termination and informed written maternal consents, and with approval of the local University Hospital Ethical Review Committees. All human fetal brain samples were anonymized. All animal experiments were performed in accordance with the German Animal Welfare legislation (“Tierschutzgesetz”). All procedures pertaining to animal experiments were approved by the Governmental IACUC ("Landesdirektion Sachsen”) and overseen by the Institutional Animal Welfare Officer(s). Mice were anaesthetised using isofluorane during the in utero electroporation procedure. Mice were killed via cervical dislocation. The license numbers concerned by the present experiments with mice are: 24–9168.11-9/2009-2 (in utero work, tamoxifen, BrdU) and 24–9168.24-9/2012-1 (tissue collection without prior in vivo experimentation).

### Mice

Mice were maintained in strict pathogen-free conditions in the animal facility of the Max Planck Institute of Molecular Cell Biology and Genetics, Dresden, Germany. To characterize the *Tis21*–CreER^T2^ mouse line described below, females were crossed with either the *Tis21*–GFP knock-in homozygous males [16] or the RCE:LoxP line [56]. To perform the in utero electroporation experiments with heterozygous *Tis21*–CreER^T2^ embryos as described below, homozogyous *Tis21*–CreER^T2^ males were crossed with wildtype C57BL/6JOlaHSD females. For the double-transgenic mice, homozogyous *Tis21*–CreER^T2^ mice were crossed with heterozygous *JoP6* mice. To study the loss of function of Pax6 on bRG generation, heterozygous *Sey* mice were crossed with one another. The day of the vaginal plug was defined as E0.5. *Tis21*–GFP [16], *hACTB*–Flpe [95], RCE:LoxP [56], and *JoP6* [58] mouse lines were genotyped as previously described. Offspring from the above crossings were genotyped for the *Tis21*–CreER^T2^ allele by PCR using standard procedure as described below.

### Generation of the *Tis21*–CreER^T2^ Mouse Line

#### Targeting construct

The bacterial artificial chromosome (BAC) bMQ-284G14 (≈130 kb, 129S7/SvEv mouse strain, Sanger institute), containing the mouse *Tis21* gene (≈0.2 kb exon 1, ≈1.2 kb intron 1, ≈2.5 kb exon 2) flanked by ≈80 kb 5' and ≈31 kb 3' [65], was used to create the targeting construct p15A-*Tis21*–CreER^T2^-neo (S1Ai Fig) for the generation of mouse embryonic stem (ES) cells in which the entire coding sequence of exon1 of one *Tis21* allele (S1Aii Fig) was replaced by a CreER^T2^ cassette followed by a neomycin cassette flanked by FRT sites (S1Aiii Fig, referred to as *Tis21*–CreER^T2^-neo). The targeting construct was cloned using the Red/ET homologous recombination technology [96]. At each of the cloning steps described below, plasmids were verified by sequencing.

First, towards obtaining the intermediate plasmid R6K-CreER^T2^-neo, we generated two PCR products using *PGK*–gb2-neo (neomycin cassette, Genebridges) and R6K-ampicillin (Genebridges) as templates, (i) Tis21-PGK-gb2-neo, which contains a neomycin selection cassette flanked by two FRT sites; and (ii) Tis21-R6K-ori, which contains the R6K replication origin. For PCR amplification of Tis21-PGK-gb2-neo, we used the primer pair neo-forward (for all primer sequences, see below), consisting of a 35 bp sequence identical to the 3’ end of the CreER^T2^ cassette, followed by a BamHI site, an FRT site, and 22 bp of the 5' flanking sequence of the neomycin cassette; and neo-reverse, consisting of 40 bp of the 5’ flanking sequence of the *Tis21* intron, followed by a HindIII and an EcoRI site, an FRT site, and 22 bp of the 3' flanking sequence of the neomycin cassette. For PCR amplification of Tis21-R6K-ori, we used the primer pair R6K-forward, consisting of an EcoRI and a HindIII site contiguous with a 40 bp sequence identical to the most 5’ sequence of the *Tis21* intron, followed by a PacI and an NdeI site, and 21 bp of the 5' flanking sequence of the R6K replication origin; and R6K-reverse, consisting of a 35 bp sequence identical to the 5’ flanking sequence of the CreER^T2^ cassette, followed by a 39 bp sequence identical to the sequence 3' to the *Tis21* translational start codon, an NdeI and a PacI site, and 21 bp of the 3' flanking sequence of the R6K replication origin.

A mixture of Tis21-PGK-gb2-neo and Tis21-R6K-ori was electroporated into *Escherichia coli* strain HS996-pir116, containing the plasmids pKS-CreER^T2^ (a gift from Dr. Gord Fishell) and pSC101-BAD-gbaA [96], for homologous recombination, followed by kanamycin selection to obtain the intermediate plasmid R6K-CreER^T2^-neo.

Second, the BAC bMQ-284G14-*Tis21*–CreER^T2^-neo was derived from the BAC bMQ-284G14 by replacing the entire coding sequence of the *Tis21* exon1 with a CreER^T2^ cassette followed by a neomycin cassette flanked by FRT sites, carrying out a second round of homologous recombination as follows. The CreER^T2^-neo fragment released from R6K-CreER^T2^-neo by NdeI and PacI digestion was electroporated into *E*. *coli* strain DH10B containing the BAC bMQ-284G14 and pSC101-BAD-gbaA for homologous recombination, followed by kanamycin selection to obtain the BAC bMQ-284G14-*Tis21*–CreER^T2^-neo.

Third, the final targeting construct, p15A-*Tis21*–CreER^T2^-neo, was generated from the BAC bMQ-284G14-*Tis21*–CreER^T2^-neo as follows. Using pACYC177 (New England Biolabs) as a template, we generated the PCR product p15A-ori-ampicillin, which consists of the p15A replication origin and the ampicillin selection cassette from pACYC177, and 40 bp each of *Tis21* homologous sequence located ≈4.8 kb 5' and ≈7.4 kb 3' to the *Tis21* translational start codon, respectively. For this purpose, we used the primer pair p15A-forward, consisting of 40 bp of the 5' *Tis21* homologous sequence, followed by an NdeI and a PacI site, and 21 bp of 5' flanking sequence of the p15A replication origin; and p15A-reverse, consisting of 40 bp of the 3' Tis21 homologous sequence, followed by a PacI and an NdeI site, and 21 bp of 3' flanking sequence of the ampicillin selection cassette. p15A-ori-ampicillin was electroporated into *E*. *coli* strain DH10B containing the BAC bMQ-284G14-*Tis21*–CreER^T2^-neo and the plasmid pSC101-BAD-gbaA for homologous recombination, followed by ampicillin selection to obtain p15A-*Tis21*–CreER^T2^-neo (S1Ai Fig), which contains ≈12.2 kb genomic sequence harbouring the *Tis21* locus with ≈4.8 kb 5’ and ≈7.4 kb 3’ to the *Tis21* translational start codon.

#### Homologous recombination in embryonic stem cells

Homologous recombination of the targeting construct p15A-*Tis21*–CreER^T2^-neo in mouse ES cells was performed essentially as described previously [65]. Southern blot analysis of individual clones was carried out as described [65], using antisense RNA probes for the 5’ and 3’ regions of *Tis21* [65] and Cre [97] (S1Aii and S1Aiii Fig).

#### *Tis21*–CreER^T2^ mouse line

ES cells carrying a *Tis21*–CreER^T2^-neo allele (S1Aiii Fig) were used to generate *Tis21*–CreER^T2^ knock-in mice (S1Aiv Fig) essentially as described previously [65].

#### Genotyping

For genotyping, mouse tail DNA was prepared using the Sigma REDExtract-N-Amp Tissue PCR Kit according to the manufacturer’s instructions. *Tis21*–CreER^T2^-neo and *Tis21*–CreER^T2^ mice were genotyped by PCR amplification using the primer pairs P1 & P2 (S1Aii Fig) and P1 & P3 (S1Aiii and S1Aiv Fig), yielding a 269-bp product for the *Tis21* wildtype allele and a 413-bp product for the *Tis21*–CreER^T2^-neo and *Tis21*–CreER^T2^ alleles, respectively. *Tis21*–CreER^T2^-neo and *Tis21*–CreER^T2^ alleles were distinguished by PCR amplification using the primer pairs P4 & P6 (S1Aiii Fig) and P5 & P6 (S1Aiv Fig), yielding a 639-bp product for the *Tis21*–CreER^T2^-neo allele and a 349-bp product for the *Tis21*–CreER^T2^ allele, respectively.

### Pax6 and Control Expression Plasmids

To obtain pCAGGS–LoxP-GAP43-GFP-LoxP-nRFP, we first generated the intermediate plasmid pCAGGS–nRFP. RFP containing 3 C-terminal tandem SV40 nuclear localization signals (nRFP) was PCR-amplified using pDSV-mRFPnls [98] as template and the primer pair nRFP-forward & nRFP-reverse. The nRFP PCR product was cloned into the pCAGGS eukaryotic expression vector [99] opened with AgeI and EcoRI, yielding pCAGGS–nRFP. Subsequently, the LoxP-GAP43-GFP-LoxP cassette was PCR-amplified using a DFRS plasmid harboring GAP43-GFP [100] as template and the primer pair LoxP-GAP43-GFP-forward & LoxP-GAP43-GFP-reverse. The LoxP-GAP43-GFP-LoxP PCR product was cloned into the pCAGGS–nRFP vector opened with AgeI and XhoI, yielding pCAGGS–LoxP-GAP43-GFP-LoxP-nRFP (Fig 2A top).

To obtain pCAGGS–LoxP-GAP43-GFP-LoxP-Pax6-IRES-nRFP (referred to as Pax6-expressing plasmid), the Pax6 and IRES sequences were amplified from DNA constructs kindly provided by Magdalena Götz [44], using the primer pair Pax6-forward & IRES-reverse. The PCR product was cloned into the control plasmid opened with XhoI, yielding the Pax6-expressing plasmid pCAGGS–LoxP-GAP43-GFP-LoxP-Pax6-IRES-nRFP (Fig 2A bottom).

### Primers

#### Primers for BAC modification

neo-forward: 5’ TTGTGGTTTGTCCAAACTCATCAATGTATCTTAAGGGATCCGAAGTTCCTATTCTCTAGAAAGTATAGGAACTTCAGTTTAAACGCGGCCGCATTCT

neo-reverse: 5’ GCCGCGCCCGAGTGGTTCCAAGACCCCGACGTGTGCTCACAAGCTTGAATTCGAAGTTCCTATACTTTCTAGAGAATAGGAACTTCTTTAAACGGCGCGCCGCACACA

R6K-forward: 5’ GAATTCAAGCTTGTGAGCACACGTCGGGGTCTTGGAACCACTCGGGCGCGGCTTAATTAACATATGGGTGCGAATAAGGGACAGTGA

R6K-reverse: 5’ TGCCCAGTGCCTCACGACCAACTTCTGCAGCTTAAACCGGTGGCTGAGGAAGTAGCTGTATTAGAACGATGATGCATATGTTAATTAATCCGCTTCCTTTAGCAGCCCT

p15A-forward: 5’ TGACGTATTTTAAACCCTAGAGAACCATTTTTGACAATGTCATATGTTAATTAAGCGCTAGCGGAGTGTATACTG

p15A-reverse: 5’ CACCCAGGGAGAGGGCATTTAAACTCTGACGGACATCAGGTTAATTAACATATGGATCCTAGAGCGCACGAATGA

#### Genotyping primers

P1: 5’ GAGTGGTATGAAAGGCGCAGC

P2: 5’ TTCCAAGACCCCGACGTGTGCTCAC

P3: 5’ CTGAACATGTCCATCAGGTTCTTGC

P4: 5’ ATGGTGGAAAATGGCCGCTTTTCTGGATTC

P5: 5’ ATTCTAGTTGTGGTTTGTCCAAACTCATC

P6: 5’ TGTTGTCAGGGTCTCAGAATGTACTCAAG

#### Primers for cloning of Pax6 and control expression plasmids

nRFP-forward: ATTACCGGTGGTACCCTCGAGATGGCCTCCTCCGAGGACGTC

nRFP-reverse: ATTGAATTCCTATACCTTTCTCTTCTTTTTTGGATCTAC

LoxP-GAP43-GFP-forward: AATACCGGTATAACTTCGTATAGCATACATTATACGAAGTTATATGCTGTGCTGTATGAGAAGAACC

LoxP-GAP43-GFP-reverse: TAACTCGAGATAACTTCGTATAATGTATGCTATACGAAGTTATTTTATTTTTAATTTTATTCTGCCCCAGCTGGTTCTTTCC

Pax6-forward:

CCGTCGACATGCAGAACAGTCACAGCGGAGTG

IRES-reverse:

CCGTCGACTGTGGCCATATTATCATCGTG

### Cell Culture

HEK293T cells were plated at 5x10^4^ cells on 24-well plates and kept in culture in DMEM supplemented with 10% fetal calf serum. At 24 h after plating, cells were transfected, using 1 μl of Lipofectamine2000 (Invitrogen), with 250 ng of pCAGGs-Cre [101] and/or 250 ng of either control or Pax6-expressing plasmids diluted with serum-free DMEM. Cells were incubated for 48 h, followed by fixation with 4% paraformaldehyde in 120 mM phosphate buffer pH 7.4 for 10 mins. The paraformaldehyde was then removed and cells were kept in PBS until further processing.

### Tamoxifen Preparation and Administration

Tamoxifen (Sigma) was dissolved in corn oil at 20 mg ml^-1^. Unless specified otherwise, tamoxifen was administered orally via gavage (0.1 ml) to pregnant dams carrying E12.5 embryos. This single dose was administered when animals were killed at E13.5. When animals were killed at E14.5, tamoxifen was administered at E12.5 and at E13.5 (see Fig 2B). When animals were killed at E15.5 or later, tamoxifen was administered at E12.5, at E13.5 and at E14.5 (see Fig 5D and Fig 7A). For the *Tis21*–CreER^T2^: *JoP6* experiments, tamoxifen was administered orally (0.2 ml) to pregnant dams carrying E13.5 embryos.

### In Utero Electroporation

In utero electroporation was carried out essentially as previously described [100,102]. Briefly, tamoxifen-treated pregnant dams carrying E13.5 embryos were anesthesized using isofluorane. Embryos were injected intraventricularly either with 0.5–3 mg ml^-1^ control or Pax6-expressing plasmid in PBS containing 0.25% Fast Green (Sigma) using a glass micropipette followed by electroporation (30 V, six 50-msec pulses with 1 sec intervals). Electroporated brains were dissected at the indicated developmental stages and fixed for 20–70 h at 4°C in 4% paraformaldehyde in phosphate buffer for further analysis.

### Thymidine Analog Labeling

Single EdU pulses were administered by injecting 0.1 ml of 1 mg ml^-1^ EdU intraperitoneally into pregnant dams carrying embryos of the indicated developmental stages.

For the cell cycle re-entry experiments, we injected such a single pulse of EdU at E14.5 and sacrificed the animals 24 h later (Fig 6A). At this developmental stage, the length of S+G2+M-phase of cortical progenitors is ≤11 h [52], and a single EdU pulse is unlikely to be effective for >5 h [103]. Hence, the 24 h period between the EdU administration and analysis should be more than sufficient for essentially all cortical progenitors that incorporated EdU and that had been derived from electroporated aRG (i.e., that were RFP+) to go through M-phase, and thus for determining by Ki67 immunofluorescence whether or not the resulting daughter cells had re-entered the cell cycle.

For the dual pulse chase experiments, 0.1 ml of 1 mg ml^-1^ of IdU and BrdU were sequentially injected intraperitoneally into pregnant dams carrying embryos of the indicated developmental stages (S6A Fig). The length of S-phase was calculated as described previously [61].

### Live Imaging

It has previously been shown that electroporation does not randomly target APs irrespective of the phase of the cell cycle, but preferentially targets APs in late S-, G2- and M-phase [104]. Conditional Pax6 expression upon electroporation would thus be confined to a synchronized cohort of progenitors, which precludes the use of cumulative labeling with a thymidine analog to determine the length of the cell cycle and its various phases. We therefore used live imaging to measure the cell cycle length of electroporated Tis21-positive aRG. In these analyses, we have exploited the fact that the RFP+ cells still contain residual membrane-GFP fluorescence (either by inheritance, or because not all plasmid copies electroporated into a given aRG underwent Cre recombination, or both).

Live time-lapse imaging of dorsolateral telencephalon tissue in organotypic slice culture was prepared and carried out as previously described [67]. Stacks of 1024 x 1024 pixels x 18–21 optical sections (xyzt sampling: 0.346 × 0.346 × 2.5 μm × 22 or 24 min) were acquired for at least 48 h, using a confocal laser-scanning microscope LSM 780 equipped with a 40× C-Apochromat 1.2 N.A. W objective (Carl Zeiss, Germany).

AP divisions were defined as those occurring at the ventricular surface. The time period between two successive mitoses of the neurogenic aRG is taken to be the length of the cell cycle, Tc.

In addition, we used live imaging to track the fate of the bRG progeny and for the reconstruction of the RFP-positive bRG lineage tree. We defined bRG divisions as those occurring away from the ventricular surface (with no apical contact) and as BPs exhibiting a basally and/or apically directed process just prior to, and often persisting through, mitosis. We included only RFP-positive bRG that had undergone division and tracked their progeny for at least an additional 20 h (i.e., for a time period longer than the average Tc of self-renewing bRG).

### Immunofluorescence

For immunofluorescence of transfected cells [65], fixed cells were permeabilised with 0.3% Triton X-100 in PBS for 30 min and then quenched with 0.1 M glycine in PBS for 30 min. Cells were sequentially incubated with primary antibodies for 3 h followed by secondary antibodies for 1 h at room temperature. Coverslips were mounted onto glass slides using Mowiol.

For vibratome sectioning [105], fixed brains were embedded in 3% low-melting agarose. Sections (50–70 μm) were cut using a vibratome (Leica 1000) and were stored in PBS (maximally for 2 wk) until further processing. For cryosectioning [105], fixed brains were equilibrated in 30% (wt/vol) sucrose in PBS overnight at 4°C. Brains were embedded with Tissue-TEK (O.C.T, Sakura Finetek) and stored at −20°C. Brains were cryosectioned at 10–12 μm. Cryosections were rehydrated with PBS before further processing. Both vibratome and cryosections were subjected to an antigen retrieval protocol as follows. Unless indicated otherwise, sections were heated in 0.01 M citrate buffer pH 6.0 at 70°C for 1 h. For comparative quantification of Pax6 and phosphohistone H3 immunofluorescence levels in mouse and human mitotic APs, cryosections of paraformaldehyde-fixed embryonic mouse and fetal human neocortex were heated in the citrate buffer using a microwave oven at 800 W for 1 min followed by 140 W for 10 min. Sections were permeabilized using 0.3% Triton X-100 in PBS for 30 min and quenched with 0.1 M glycine for 30 min. Sections were then incubated with primary antibody overnight at 4°C, followed by secondary antibody for 1 h at room temperature in a solution of 0.2% gelatin, 300 mM NaCl, and 0.3% Triton X-100 in PBS. Floating sections were mounted to Superfrost Plus microscope slides (Thermo Scientific) using Mowiol (Merck Biosciences). For BrdU and IdU detection, slices were processed after RFP immunofluorescence as follows. An additional antigen retrieval step was performed by using HCl (2 N HCL, 30 min incubation at 37°C). Slices were then blocked with 10% goat serum and incubated for 3 h at room temperature followed by 1 h of secondary antibody incubation.

The following primary antibodies were used; ßIII-tubulin (Sigma, T8578 1:500), BrdU and IdU (Becton Dickinson, 347580, 1:100), BrdU only (Abcam, ab6326, 1:100), Brn2 (Santa Cruz, SC-6029, 1:200), caspase-3 (Abcam, ab2302, 1:500), cyclinD1 (Thermo, MA1-39546, 1:200), γ-tubulin (Sigma, T5326, 1:200), GFAP (Millipore, MAB 360, 1:500), HSV tag (Abcam, ab19354, 1:200), Ki67 (Abcam, ab16667, 1:300), nestin (Abcam, AB5968, 1:200), Olig2 (Thermo, MA5-15810, 1:200), Pax6 (Covance, PRB-278P, 1:200), PCNA (Millipore, MAB424, 1:100), PH3 (Millipore, 06–570, 1:500), phosphovimentin (Abcam, ab22651, 1:500), RFP (Chemotek, 5F8, 1:500), SATB2 (Abcam, ab51502, 1:200), Sox2 (Santa Cruz, SC17320, 1:500), Tbr1 (Abcam, ab31940, 1:200), and Tbr2 (Abcam, ab23345, 1:200). Alexa Fluor 488, 594, 647 labeled secondary antibodies (Molecular Probes) were used (1:500). Nuclei were counterstained with DAPI (Sigma, 1:1,000). In case of thymidine analog-labeled samples (i.e., BrdU, EdU, and IdU), sections were postfixed with 4% paraformaldehyde for 20 min after the secondary antibody incubation. Incorporated EdU was detected using the Click-iT EdU kit with Alexa Fluor 647 (Invitrogen) as described previously [52].

### Daughter Cell Pair Analysis

Vibratome sections (50-μm) and 12-μm cryosections were used for Tbr2 and PCNA analysis, respectively. We used similar criteria as used previously [65], with some modification. In summary, we examined only sparsely electroporated areas and defined two closely located RFP-positive cells as a pair of daughter cells derived from a single electroporated AP if (i) no other RFP-positive cells were observed within the distance of one cell body around the two cells in the z-stack; (ii) both cells exhibited the same RFP fluorescence intensity; and (iii) the two RFP-positive cells were aligned in the same radial axis and were located above one another. In the case of the Tbr2–/Tbr2– daughter cell pairs, we measured the distance of the center of the nucleus of the ventricular-most daughter cell from the ventricular surface in Fiji.

### Image Acquisition

Fluorescence images were acquired using a Zeiss 700 confocal microscope using 25x and 63x objectives. Images were taken as either 2.1 μm (25x) or 0.9 μm (63x) single optical sections. All images used for scoring of parameters in control versus conditional Pax6 expression had comparable RFP fluorescence intensities. All images showing these parameters for control versus conditional Pax6 expression were acquired with the same settings during each microscope session. Images taken as tile scans were stitched together using the ZEN software (Zeiss). Quantifications were performed using Fiji. Whole-brain images were acquired with an Olympus SZX12 stereomicroscope.

### Determination of Cleavage Plane Orientation

Cleavage plane orientation of electroporated mitotic APs and BPs was measured in 2-D based on the position of the DAPI-stained sister chromatids during late anaphase and was expressed relative to the ventricular surface. A cleavage plane parallel to the ventricular surface (i.e., horizontal cleavage plane) is defined as 0°.

### Identification of Germinal Zones, Cell Counting, Quantification of Immunofluorescence Intensity, and Statistical Analysis

Germinal zones were identified based on their different histological characteristics. The VZ was identified as the ventricular-most layer of densely packed, radially aligned, elongated nuclei. The SVZ was identified as the layer basal to the VZ containing less densely packed, randomly orientated, rounded nuclei.

Unless specified otherwise, cells were counted in a rectangular area, 200-μm wide at the ventricular surface, within the electroporated region of the dorsolateral telencephalon. For quantifications using double-transgenic animals (*Tis21*–CreER^T2^: *JoP6*), cells were counted in a rectangular area, 100-μm wide at the ventricular surface. Cells were counted without using pseudocolour. All quantifications were confined to RFP-positive cells only, with the exception of (i) the determination of the total nuclei present in the VZ (Fig 4J), and (ii) the analyses of the neocortex of the double-transgenic animals, (*Tis21*–CreER^T2^: *JoP6*); in both cases, all DAPI-stained nuclei were quantified.

For quantification of immunofluorescence intensity levels, the area of the nucleus of interphase cells in VZ and SVZ was selected using the DAPI staining as a guide, and the area of the cell body of mitotic APs was selected using the phosphohistone H3 immunofluorescence as a guide. Selected areas were quantified using Fiji [106].

Data was further processed using the Prism software (GraphPad software). Student's *t* test was used to determine statistical significance.

## Supporting Information

S1 Data Data for all figures and tables. (XLSX)

S1 Fig Generation of *Tis21*–CreER^T2^ mice. (**A**) Generation of the *Tis21*-CreER^T2^ allele by homologous recombination. (i–iii) Steps carried out with ES cells. Using the targeting construct shown (i), the coding sequence in Exon1 of *Tis21* (shaded box, ii) was replaced by a CreER^T2^ cassette (yellow box, i) followed by a neomycin cassette (neo, red box, i) flanked by FRT sites (green triangles, i), yielding the *Tis21*-CreER^T2^-neo allele (iii). (iii–iv) Steps carried out with mice. Removal of the neomycin cassette by crossing mice carrying the *Tis21*-CreER^T2^-neo allele (iii) with transgenic *hACTB*-FLPe mice expressing FLPe recombinase, yielding mice carrying the *Tis21*-CreER^T2^ allele (iv). (i–iv) Shaded boxes, *Tis21* ORF; blue and red triangles, 5’ and 3’ ends, respectively, of homology between the targeting construct (i) and the *Tis21* wildtype allele (ii); P1–P6, primers used for genotyping PCR; blue, yellow, and red bars, location of probes used for Southern blot analyses. For details, see Experimental Procedures. (**B**–**D**) Southern blot analysis of genomic DNA from wildtype (Wt) and transgenic *Tis21*-CreER^T2^-neo heterozygous (Tg) embryonic stem cells. DNA was digested with KpnI (**B**), HindIII (**C**) or EcoRI (**D**), and hybridized with either the 5’ probe, the 3’ probe, or the Cre probe, respectively, as indicated by the blue, red, or yellow bars in (**A**, ii, and iii). Solid arrowheads in (**B**), 17.4 kb fragment of KpnI-digested wt allele (**A**, ii); in (**C**), 13.1 kb fragment of HindIII-digested wt allele (**A**, ii); open arrowheads in (**B**), 11.9 kb fragment of KpnI-digested *Tis21*-CreER^T2^-neo allele (**A**, iii); in (**C**), 10.6 kb fragment of HindIII digested *Tis21*-CreER^T2^-neo allele (**A**, iii); in (**D**), 10.0 kb fragment of EcoRI digested *Tis21*-CreER^T2^-neo allele (**A**, iii).
(TIF)

S2 Fig Validation of the Pax6-expressing plasmid in HEK293T cells. HEK293T cells were transfected with Pax6-expressing plasmid (**A**), control plasmid plus pCAGGs-Cre (**B**), or Pax6-expressing plasmid plus pCAGGs-Cre (**C**), followed 48 h later by Pax6 immunofluorescence (white) and GFP (green) and RFP (red) fluorescence, combined with DAPI staining (blue). Scale bars, 20 μm.
(TIF)

S3 Fig Cre recombination in *Tis21*–CreER^T2^ mice is tamoxifen-dependent. Dorsolateral telencephalon of E14.5 *Tis21*–CreER^T2^ heterozygous mice electroporated at E13.5 with Pax6-expressing plasmid without (**top**) or with (**bottom**) tamoxifen pretreatment (see Fig 2B). GFP (green) and RFP (red) fluorescence, combined with DAPI staining (blue), on coronal 50-μm vibratome sections. Scale bars, 20 μm.
(TIF)

S4 Fig Conditional Pax6 expression in Tis21-positive APs does not significantly alter progeny location after 24 h. Dorsolateral telencephalon of tamoxifen-treated E14.5 *Tis21*–CreER^T2^ heterozygous mice electroporated at E13.5 with Pax6-expressing (**A**,**B**) or control (**B**) plasmid (see Fig 2B). (**A**) Representative example of the distribution of RFP-positive cells (red) in the cortical wall, divided into ten equally sized bins, with the bin containing the ventricular surface being defined as bin 1. Blue, DAPI staining; coronal 50-μm vibratome sections. (**B**) Quantification of the distribution of RFP-positive cells across the ten bins (see **A**), expressed as percentage of all RFP-positive cells in the cortical wall (200-μm wide area), upon control (Con, white) and Pax6 (black) electroporation. Mean of three independent experiments, each being the average of two to four embryos. Error bars, SEM.
(TIF)

S5 Fig Conditional Pax6 expression in Tis21-positive APs does not induce apoptosis. Dorsolateral telencephalon of tamoxifen-treated E14.5 *Tis21*–CreER^T2^ heterozygous mice electroporated at E13.5 with control (**A**,**C**) or Pax6-expressing (**B**,**C**) plasmid (see Fig 2B). (**A**,**B**) Caspase-3 immunofluorescence (white) and RFP fluorescence (red), combined with DAPI staining (blue), on coronal 50-μm vibratome sections. Dashed white lines, ventricular surface. Scale bars, 20 μm. (**C**) Quantification of caspase-3- and RFP-positive cells in the cortical wall (200-μm wide area), upon control (Con, white) and Pax6 (black) electroporation. Mean of three independent experiments, each being the average of two to four embryos. Error bars, SEM.
(TIF)

S6 Fig Longer S-phase in the progeny of Tis21-positive APs upon conditional Pax6 expression. (**A**) Flow scheme of the experiment. (**B–E**) Dorsolateral telencephalon of tamoxifen-treated E14.5 *Tis21*–CreER^T2^ heterozygous mice electroporated at E13.5 with control (**B,D,E**) or Pax6-expressing (**C–E**) plasmid. IdU and BrdU were injected at 2 h and 0.5 h, respectively, before sacrifice. (**B**,**C**) RFP (white), IdU & BrdU (red), and BrdU only (green) immunofluorescence, on coronal 20-μm cryosections. Scale bars, 20 μm and 5 μm (insets). Yellow arrows, triple-positive cells (RFP+, IdU+, BrdU+); yellow arrowheads, double-positive cells (RFP+, IdU+, BrdU–); dashed white lines, ventricular surface. Insets show representative examples of RFP+ & IdU+ nuclei (outlined by dashed yellow lines) at higher magnification that are either BrdU+ (yellow arrows) or BrdU–(yellow arrowheads). (**D**) Quantification of RFP+, IdU+, & BrdU+ triple-positive cells (white) and RFP+, IdU+, and BrdU–double-positive cells (grey) in the cortical wall expressed as percentage of all cells that are both IdU+ & RFP+ in the cortical wall (200-μm wide area), upon control (Con) and Pax6 electroporation. (**E**) Quantification of S-phase length upon control (Con, circles) and Pax6 (squares) electroporation. Mean of three embryos from two independent experiments. Error bars, SEM. ** *p* < 0.01.
(TIF)

S7 Fig Daughter cell pairs derived from electroporated Tis21-positive APs are cycling. Dorsolateral telencephalon of tamoxifen-treated E14.5 *Tis21*–CreER^T2^ heterozygous mice electroporated at E13.5 with control (left) or Pax6-expressing (right) plasmid (see Fig 2B), showing representative examples of RFP+ (red) and PCNA+ (white) double-positive daughter cell pairs (dashed yellow lines) derived from Tis21-positive electroporated APs (12-μm cryosections). Note that all daughter cell pairs analyzed (10 pairs each for control and Pax6) were PCNA+, irrespective of the absence or presence of Tbr2 immunoreactivity. Dashed white lines, ventricular surface. Scale bars, 10 μm.
(TIF)

S8 Fig Distance of daughter cell pairs derived from electroporated Tis21-positive APs from ventricular surface. Distance of nuclei of the Tbr2–/Tbr2–, Tbr2+/Tbr2–, and Tbr2+/Tbr2+ daughter cell pairs from the ventricular surface upon control (Con, white) and Pax6 (black) electroporation. Data indicate the position of the ventricular-most nucleus of each pair (see Materials and Methods). Light and dark blue background indicates the areas within <27 μm and ≥27 μm from the ventricular surface, respectively. Mean of 2–15 cell pairs; error bars, SEM; yellow dots, individual values.
(TIF)

S9 Fig bRG generated upon conditional Pax6 expression in Tis21-positive APs exhibit typical bRG characteristics. Dorsolateral telencephalon of tamoxifen-treated E14.5 *Tis21*–CreER^T2^ heterozygous mice electroporated at E13.5 with control (**E**) or Pax6-expressing (**A**–**E**) plasmid (see Fig 2B). (**A–D**) bRG identified by residual membrane-GFP fluorescence (maximum intensity projections of stacks of 5 (**A**), 9 (**B**), and 11 (**C**) images, single optical sections (**D**)). Yellow arrowheads, basal process; white arrow, centrosome location; yellow boxes indicate the cell body (yellow dashed lines) that is shown as single optical sections at higher magnification in the small panels; Sox2 (white, **A**), nestin (white, **B**), Tbr2 (white, **C**), and γ-tubulin (white, **D**) immunofluorescence, together with GFP (green) and RFP (red) fluorescence, combined with DAPI staining (**A**,**B**,**D**), on coronal 50-μm vibratome sections. Scale bars, 20 μm and 5 μm (small panels). (**E**) Quantification of basal mitotic somal translocation of bRG in control (Con, circles) and conditional Pax6 expression (Pax6, squares). Mean ± SEM. Control, 12 cells; Pax6, 20 cells.
(TIF)

S10 Fig Reconstruction of bRG lineage trees as observed by live time-lapse imaging. Summary of the 13 bRG-derived lineage trees observed upon live time-lapse imaging of E14.5 organotypic slices prepared from dorsolateral telencephalon of tamoxifen-treated *Tis21*–CreER^T2^ heterozygous mice electroporated with control or Pax6-expressing plasmid (see Fig 7). Control, 7 bRG divisions; Pax6, 6 bRG divisions. Tc, total cell cycle length; red circles, bRG; yellow circles, bIPs; grey circles, no further mitosis detected for progeny until 20 h; white circles, unknown cell type.
(TIF)

S11 Fig Time-lapse imaging of bRG generated upon conditional Pax6 expression and its progeny—asymmetric neurogenic division. (**A**) Flow scheme of experiment. (**B**) Lineage tree reconstruction of bRG division. (**C**) Live time-lapse imaging of organotypic slice of dorsolateral telencephalon of tamoxifen-treated E14.5 *Tis21*–CreER^T2^ heterozygous mice electroporated at E13.5 with Pax6-expressing plasmid. Membrane-GFP fluorescence, single optical sections. 00:00 (hh:mm) denotes the start of mitosis. Yellow arrowheads, mother bRG; white arrowheads, basal process; green and red arrowheads, bRG daughter and neuron daughter, respectively, of mother bRG; green arrows, daughter cells of bRG daughter.
(TIF)

S12 Fig Most progeny of conditionally Pax6 expressing Tis21-positive APs exhibits heterotopia after 4 d. Dorsolateral telencephalon of tamoxifen-treated E17.5 *Tis21*–CreER^T2^ heterozygous embryos electroporated at E13.5 with control (**A**,**D,E**) or Pax6-expressing (**B–E**) plasmid subjected to a single EdU pulse (not illustrated) 10 h after electroporation (E14.0). (**A–C**) Pax6 immunofluorescence (white) and RFP fluorescence (red), combined with DAPI staining (blue), on coronal 50-μm vibratome sections. Images in (**C**) show representative examples at a higher magnification of RFP+ and Pax6+ double-positive progeny in the cortical plate (left) and exhibiting heterotopia in the intermediate zone (right); note the higher RFP and Pax6 (immuno)fluorescence level in the progeny exhibiting heterotopia. Scale bars, 20 μm. (**D**) Quantification of RFP+ nuclei in the cortical plate (left) and in the remainder of the cortical wall (right), expressed as percentage of all RFP-positive cells in the cortical wall (200-μm wide area), upon control (Con, white) and Pax6 (black) electroporation. Mean of eight embryos from at least two independent experiments. (**E**) Pax6 immunofluorescence intensity per cell (A.U., arbitrary units) in RFP-positive (RFP+) and-negative (RFP–) cells in cortical plate (CP, striped) and heterotopia (Ht, black) upon Pax6 electroporation, and in the cortical wall upon control (Con, white) electroporation. Mean of three independent experiments, each being the average of three embryos. (**D**,**E**) Error bars, SEM. ** *p* < 0.01, *** *p* < 0.001.
(TIF)

S13 Fig Progeny exhibiting heterotopia 4 days after conditional Pax6 expression in Tis21-positive APs are immature neurons. Dorsolateral telencephalon of tamoxifen-treated E17.5 *Tis21*–CreER^T2^ heterozygous embryos electroporated at E13.5 with Pax6 expressing plasmid and subjected to a single EdU pulse (not illustrated) 10 h after electroporation (E14.0), showing progeny exhibiting heterotopia. Olig2 (**A**), GFAP (**B**), NeuN (**C**), and Tuj1 (**D**) immunofluorescence (white), together with RFP (red) and GFP (green) fluorescence and DAPI staining (blue), on coronal 50-μm vibratome sections. The area indicated by the yellow box in (**D**) is shown at higher magnification in (**E**); note the colocalization of Tuj1 immunofluorescence and GFP fluorescence. Scale bars, 10 μm.
(TIF)

S14 Fig Endogenous Pax6 levels in mitotic APs are higher in fetal human than embryonic mouse neocortex. VZ of the rostral neocortex of wildtype E14.5 mouse (top) and gestational week (GW) 12 human was analyzed by double immunofluorescence for Pax6 and phosphohistone H3 (PH3). (**A**) Representative images showing Pax6 (green) and PH3 (red) immunofluorescence, combined with DAPI staining (blue), on coronal 12-μm cryosections. Dashed white lines, ventricular surface; yellow arrowheads, mitotic APs. Scale bars, 20 μm. (**B**) Quantification of Pax6 (left) and PH3 (right) immunofluorescence intensity per mitotic AP (A.U., arbitrary units). Note the higher Pax6 level in fetal human APs as compared to embryonic mouse APs, and the equal PH3 immunoreactivity level. Mean of 34 (mouse, white) and 32 (human, black) mitotic APs; error bars, SEM. *** *p* <0.001.
(TIF)

S1 Movie Time-lapse imaging of bRG generated upon conditional Pax6 expression and its progeny—asymmetric neurogenic division. Time-lapse interval, 21 min; total time elapsed, 22.8 h.
(AVI)

S2 Movie Time-lapse imaging of bRG generated upon conditional Pax6 expression and its progeny—symmetric proliferative division. Time-lapse interval, 21 min; total time elapsed, 24.9 h.
(AVI)

S1 Table Cell cycle parameters of Tis21+ aRG upon control and Pax6-expressing plasmid electroporation. (DOCX)

S2 Table Cell cycle length of self-renewing Tis21+ bRG upon control and Pax6-expressing plasmid electroporation. (DOCX)

## Abbreviations:

AP: apical progenitor
aRG: apical radial glia
bIP: basal intermediate progenitor
BP: basal progenitor
bRG: basal radial glia
ECM: extracellular matrix
exoPax6: exogenous Pax6
HSV: herpes simplex virus
IRES: internal ribosome entry site
iSVZ,: inner subventricular zone
nRFP: nuclear RFP
oSVZ: outer subventricular zone
PCNA: proliferating cell nuclear antigen
SEM: standard error of the mean
SVZ: subventricular zone
Tc: total cell cycle length
VZ: ventricular zone

## References

[1] Rakic P. Evolution of the neocortex: a perspective from developmental biology. Nat Rev Neurosci. 2009;10(10):724–735. 10.1038/nrn2719 19763105 PMC2913577

[2] Kriegstein A, Noctor S, Martinez-Cerdeno V. Patterns of neural stem and progenitor cell division may underlie evolutionary cortical expansion. Nat Rev Neurosci. 2006;7(11):883–890. 17033683 10.1038/nrn2008

[3] Fish JL, Kennedy H, Dehay C, Huttner WB. Making bigger brains—the evolution of neural-progenitor-cell division. J Cell Sci. 2008;121:2783–2793. 10.1242/jcs.023465 18716282

[4] Fietz SA, Huttner WB. Cortical progenitor expansion, self-renewal and neurogenesis–a polarized perspective. Curr Opin Neurobiol. 2011;21(1):23–35. 10.1016/j.conb.2010.10.002 21036598

[5] Lui JH, Hansen DV, Kriegstein AR. Development and evolution of the human neocortex. Cell. 2011;146(1):18–36. 10.1016/j.cell.2011.06.030 21729779 PMC3610574

[6] Borrell V, Reillo I. Emerging roles of neural stem cells in cerebral cortex development and evolution. Dev Neurobiol. 2012;72(7):955–971. 10.1002/dneu.22013 22684946

[7] Franco SJ, Muller U. Shaping our minds: stem and progenitor cell diversity in the mammalian neocortex. Neuron. 2013;77(1):19–34. 10.1016/j.neuron.2012.12.022 23312513 PMC3557841

[8] Florio M, Huttner WB. Neural progenitors, neurogenesis and the evolution of the neocortex. Development. 2014;141(11):2182–2194. 10.1242/dev.090571 24866113

[9] Lewitus E, Kelava I, Kalinka AT, Tomancak P, Huttner WB. An adaptive threshold in mammalian neocortical evolution. PLoS Biol. 2014;12(11):e1002000 10.1371/journal.pbio.1002000 25405475 PMC4236020

[10] Taverna E, Götz M, Huttner WB. The cell biology of neurogenesis: toward an understanding of the development and evolution of the neocortex. Annu Rev Cell Dev Biol. 2014;30:465–502. 10.1146/annurev-cellbio-101011-155801 25000993

[11] Kriegstein AR, Götz M. Radial glia diversity: a matter of cell fate. Glia. 2003;43(1):37–43. 12761864 10.1002/glia.10250

[12] Götz M, Huttner WB. The cell biology of neurogenesis. Nat Rev Mol Cell Biol. 2005;6(10):777–788. 16314867 10.1038/nrm1739

[13] Gal JS, Morozov YM, Ayoub AE, Chatterjee M, Rakic P, Haydar TF. Molecular and morphological heterogeneity of neural precursors in the mouse neocortical proliferative zones. J Neurosci. 2006;26(3):1045–1056. 16421324 10.1523/JNEUROSCI.4499-05.2006 PMC3249619

[14] Stancik EK, Navarro-Quiroga I, Sellke R, Haydar TF. Heterogeneity in ventricular zone neural precursors contributes to neuronal fate diversity in the postnatal neocortex. J Neurosci. 2010;30(20):7028–7036. 10.1523/JNEUROSCI.6131-09.2010 20484645 PMC2909740

[15] Tyler WA, Haydar TF. Multiplex genetic fate mapping reveals a novel route of neocortical neurogenesis, which is altered in the Ts65Dn mouse model of Down syndrome. J Neurosci. 2013;33(12):5106–5019. 10.1523/JNEUROSCI.5380-12.2013 23516277 PMC3785112

[16] Haubensak W, Attardo A, Denk W, Huttner WB. Neurons arise in the basal neuroepithelium of the early mammalian telencephalon: A major site of neurogenesis. Proc Natl Acad Sci USA. 2004;101:3196–3201. 14963232 10.1073/pnas.0308600100 PMC365766

[17] Noctor SC, Martinez-Cerdeno V, Ivic L, Kriegstein AR. Cortical neurons arise in symmetric and asymmetric division zones and migrate through specific phases. Nat Neurosci. 2004;7(2):136–144. 14703572 10.1038/nn1172

[18] Miyata T, Kawaguchi A, Saito K, Kawano M, Muto T, Ogawa M. Asymmetric production of surface-dividing and non-surface-dividing cortical progenitor cells. Development. 2004;131(13):3133–3145. 15175243 10.1242/dev.01173

[19] Konno D, Shioi G, Shitamukai A, Mori A, Kiyonari H, Miyata T, et al Neuroepithelial progenitors undergo LGN-dependent planar divisions to maintain self-renewability during mammalian neurogenesis. Nat Cell Biol. 2008;10(1):93–101. 18084280 10.1038/ncb1673

[20] Noctor SC, Martinez-Cerdeno V, Kriegstein AR. Distinct behaviors of neural stem and progenitor cells underlie cortical neurogenesis. J Comp Neurol. 2008;508(1):28–44. 10.1002/cne.21669 18288691 PMC2635107

[21] Attardo A, Calegari F, Haubensak W, Wilsch-Bräuninger M, Huttner WB. Live imaging at the onset of cortical neurogenesis reveals differential appearance of the neuronal phenotype in apical versus basal progenitor progeny. PLoS ONE. 2008;3(6):e2388 10.1371/journal.pone.0002388 18545663 PMC2398773

[22] Kowalczyk T, Pontious A, Englund C, Daza RA, Bedogni F, Hodge R, et al Intermediate neuronal progenitors (basal progenitors) produce pyramidal-projection neurons for all layers of cerebral cortex. Cereb Cortex. 2009;19(10):2439–2450. 10.1093/cercor/bhn260 19168665 PMC2742596

[23] Betizeau M, Cortay V, Patti D, Pfister S, Gautier E, Bellemin-Ménard A, et al Precursor diversity and complexity of lineage relationships in the outer subventricular zone of the primate. Neuron. 2013;80:442–457. 10.1016/j.neuron.2013.09.032 24139044

[24] LaMonica BE, Lui JH, Hansen DV, Kriegstein AR. Mitotic spindle orientation predicts outer radial glial cell generation in human neocortex. Nat Commun 2013;4:1665 10.1038/ncomms2647 23575669 PMC3625970

[25] Fietz SA, Kelava I, Vogt J, Wilsch-Brauninger M, Stenzel D, Fish JL, et al OSVZ progenitors of human and ferret neocortex are epithelial-like and expand by integrin signaling. Nat Neurosci. 2010;13(6):690–699. 10.1038/nn.2553 20436478

[26] Hansen DV, Lui JH, Parker PR, Kriegstein AR. Neurogenic radial glia in the outer subventricular zone of human neocortex. Nature. 2010;464(7288):554–561. 10.1038/nature08845 20154730

[27] Reillo I, de Juan Romero C, Garcia-Cabezas MA, Borrell V. A role for intermediate radial glia in the tangential expansion of the mammalian cerebral cortex. Cereb Cortex. 2011;21(7):1674–1694. 10.1093/cercor/bhq238 21127018

[28] Wang X, Tsai JW, Lamonica B, Kriegstein AR. A new subtype of progenitor cell in the mouse embryonic neocortex. Nat Neurosci. 2011;14(5):555–561. 10.1038/nn.2807 21478886 PMC3083489

[29] Kelava I, Reillo I, Murayama AY, Kalinka AT, Stenzel D, Tomancak P, et al Abundant occurrence of basal radial glia in the subventricular zone of embryonic neocortex of a lissencephalic primate, the common marmoset Callithrix jacchus. Cereb Cortex. 2012;22(2):469–481. 10.1093/cercor/bhr301 22114084 PMC3256412

[30] Martinez-Cerdeno V, Cunningham CL, Camacho J, Antczak JL, Prakash AN, Cziep ME, et al Comparative analysis of the subventricular zone in rat, ferret and macaque: evidence for an outer subventricular zone in rodents. PLoS ONE. 2012;7(1):e30178 10.1371/journal.pone.0030178 22272298 PMC3260244

[31] Gertz CC, Lui JH, Lamonica BE, Wang X, Kriegstein AR. Diverse behaviors of outer radial glia in developing ferret and human cortex. J Neurosci. 2014;34(7):2559–2570. 10.1523/JNEUROSCI.2645-13.2014 24523546 PMC3921426

[32] Smart IH, Dehay C, Giroud P, Berland M, Kennedy H. Unique morphological features of the proliferative zones and postmitotic compartments of the neural epithelium giving rise to striate and extrastriate cortex in the monkey. Cereb Cortex. 2002;12(1):37–53. 11734531 10.1093/cercor/12.1.37 PMC1931430

[33] Molnar Z. Evolution of cerebral cortical development. Brain Behav Evol. 2011;78(1):94–107. 10.1159/000327325 21691047

[34] Borrell V, Götz M. Role of radial glial cells in cerebral cortex folding. Curr Opin Neurobiol. 2014;27:39–46. 10.1016/j.conb.2014.02.007 24632307

[35] Shitamukai A, Konno D, Matsuzaki F. Oblique radial glial divisions in the developing mouse neocortex induce self-renewing progenitors outside the germinal zone that resemble primate outer subventricular zone progenitors. J Neurosci. 2011;31(10):3683–3695. 10.1523/JNEUROSCI.4773-10.2011 21389223 PMC6622781

[36] Walther C, Gruss P. Pax-6, a murine paired box gene, is expressed in the developing CNS. Development. 1991;113(4):1435–1449. 1687460 10.1242/dev.113.4.1435

[37] Götz M, Stoykova A, Gruss P. Pax6 controls radial glia differentiation in the cerebral cortex. Neuron. 1998;21(5):1031–1044. 9856459 10.1016/s0896-6273(00)80621-2

[38] Osumi N, Shinohara H, Numayama-Tsuruta K, Maekawa M. Concise review: Pax6 transcription factor contributes to both embryonic and adult neurogenesis as a multifunctional regulator. Stem Cells. 2008;26(7):1663–1672. 10.1634/stemcells.2007-0884 18467663

[39] Georgala PA, Carr CB, Price DJ. The role of Pax6 in forebrain development. Dev Neurobiol. 2011;71(8):690–709. 10.1002/dneu.20895 21538923

[40] Warren N, Caric D, Pratt T, Clausen JA, Asavaritikrai P, Mason JO, et al The transcription factor, Pax6, is required for cell proliferation and differentiation in the developing cerebral cortex. Cereb Cortex. 1999;9(6):627–635. 10498281 10.1093/cercor/9.6.627

[41] Estivill-Torrus G, Pearson H, van Heyningen V, Price DJ, Rashbass P. Pax6 is required to regulate the cell cycle and the rate of progression from symmetrical to asymmetrical division in mammalian cortical progenitors. Development. 2002;129(2):455–466. 11807037 10.1242/dev.129.2.455

[42] Heins N, Malatesta P, Cecconi F, Nakafuku M, Tucker KL, Hack MA, et al Glial cells generate neurons: the role of the transcription factor Pax6. Nat Neurosci. 2002;5(4):308–315. 11896398 10.1038/nn828

[43] Haubst N, Georges-Labouesse E, De Arcangelis A, Mayer U, Gotz M. Basement membrane attachment is dispensable for radial glial cell fate and for proliferation, but affects positioning of neuronal subtypes. Development. 2006;133(16):3245–3254. 16873583 10.1242/dev.02486

[44] Holm PC, Mader MT, Haubst N, Wizenmann A, Sigvardsson M, Gotz M. Loss- and gain-of-function analyses reveal targets of Pax6 in the developing mouse telencephalon. Mol Cell Neurosci. 2007;34(1):99–119. 17158062 10.1016/j.mcn.2006.10.008

[45] Quinn JC, Molinek M, Martynoga BS, Zaki PA, Faedo A, Bulfone A, et al Pax6 controls cerebral cortical cell number by regulating exit from the cell cycle and specifies cortical cell identity by a cell autonomous mechanism. Dev Biol. 2007;302(1):50–65. 16979618 10.1016/j.ydbio.2006.08.035 PMC2384163

[46] Sansom SN, Griffiths DS, Faedo A, Kleinjan DJ, Ruan Y, Smith J, et al The level of the transcription factor Pax6 is essential for controlling the balance between neural stem cell self-renewal and neurogenesis. PLoS Genet. 2009;5(6):e1000511 10.1371/journal.pgen.1000511 19521500 PMC2686252

[47] Asami M, Pilz GA, Ninkovic J, Godinho L, Schroeder T, Huttner WB, et al The role of Pax6 in regulating the orientation and mode of cell division of progenitors in the mouse cerebral cortex. Development. 2011;138(23):5067–5078. 10.1242/dev.074591 22031545

[48] Georgala PA, Manuel M, Price DJ. The generation of superficial cortical layers is regulated by levels of the transcription factor Pax6. Cereb Cortex. 2011;21(1):81–94. 10.1093/cercor/bhq061 20413449 PMC3000564

[49] Mi D, Carr CB, Georgala PA, Huang YT, Manuel MN, Jeanes E, et al Pax6 exerts regional control of cortical progenitor proliferation via direct repression of Cdk6 and hypophosphorylation of pRb. Neuron. 2013;78(2):269–284. 10.1016/j.neuron.2013.02.012 23622063 PMC3898967

[50] Fietz SA, Lachmann R, Brandl H, Kircher M, Samusik N, Schroder R, et al Transcriptomes of germinal zones of human and mouse fetal neocortex suggest a role of extracellular matrix in progenitor self-renewal. Proc Natl Acad Sci USA. 2012;109(29):11836–11841. 10.1073/pnas.1209647109 22753484 PMC3406833

[51] Englund C, Fink A, Lau C, Pham D, Daza RA, Bulfone A, et al Pax6, Tbr2, and Tbr1 are expressed sequentially by radial glia, intermediate progenitor cells, and postmitotic neurons in developing neocortex. J Neurosci. 2005;25(1):247–251. 15634788 10.1523/JNEUROSCI.2899-04.2005 PMC6725189

[52] Arai Y, Pulvers JN, Haffner C, Schilling B, Nusslein I, Calegari F, et al Neural stem and progenitor cells shorten S-phase on commitment to neuron production. Nat Commun. 2011;2:154 21224845 10.1038/ncomms1155 PMC3105305

[53] Bayatti N, Moss JA, Sun L, Ambrose P, Ward JF, Lindsay S, et al A molecular neuroanatomical study of the developing human neocortex from 8 to 17 postconceptional weeks revealing the early differentiation of the subplate and subventricular zone. Cereb Cortex. 2007;18(7):1536–1548. 17965125 10.1093/cercor/bhm184 PMC2430151

[54] Mo Z, Zecevic N. Is Pax6 critical for neurogenesis in the human fetal brain? Cereb Cortex. 2007;18(6):1455–1465. 17947347 10.1093/cercor/bhm181 PMC2670483

[55] Iacopetti P, Michelini M, Stuckmann I, Oback B, Aaku-Saraste E, Huttner WB. Expression of the antiproliferative gene TIS21 at the onset of neurogenesis identifies single neuroepithelial cells that switch from proliferative to neuron-generating division. Proc Natl Acad Sci USA. 1999;96(8):4639–4644. 10200315 10.1073/pnas.96.8.4639 PMC16385

[56] Sousa VH, Miyoshi G, Hjerling-Leffler J, Karayannis T, Fishell G. Characterization of Nkx6-2-derived neocortical interneuron lineages. Cereb Cortex. 2009;19 Suppl 1:i1–10. 10.1093/cercor/bhp038 19363146 PMC2693535

[57] Anastassiadis K, Glaser S, Kranz A, Berhardt K, Stewart AF. A practical summary of site-specific recombination, conditional mutagenesis, and tamoxifen induction of CreERT2. Methods Enzymol. 2010;477:109–123. 10.1016/S0076-6879(10)77007-5 20699139

[58] Berger J, Berger S, Tuoc TC, D'Amelio M, Cecconi F, Gorski JA, et al Conditional activation of Pax6 in the developing cortex of transgenic mice causes progenitor apoptosis. Development. 2007;134(7):1311–1322. 17329367 10.1242/dev.02809

[59] Martynoga B, Drechsel D, Guillemot F. Molecular control of neurogenesis: a view from the mammalian cerebral cortex. Cold Spring Harb Perspect Biol. 2012;4(10). 10.1101/cshperspect.a008359 PMC3475166 23028117

[60] Maiorano NA, Mallamaci A. Promotion of embryonic cortico-cerebral neuronogenesis by miR-124. Neural Dev. 2009;4:40 10.1186/1749-8104-4-40 19883498 PMC2777883

[61] Martynoga B, Morrison H, Price DJ, Mason JO. Foxg1 is required for specification of ventral telencephalon and region-specific regulation of dorsal telencephalic precursor proliferation and apoptosis. Dev Biol. 2005;283(1):113–127. 15893304 10.1016/j.ydbio.2005.04.005

[62] Stenzel D, Wilsch-Brauninger M, Wong FK, Heuer H, Huttner WB. Integrin alphavbeta3 and thyroid hormones promote expansion of progenitors in embryonic neocortex. Development. 2014;141(4):795–806. 10.1242/dev.101907 24496617

[63] Farkas LM, Haffner C, Giger T, Khaitovich P, Nowick K, Birchmeier C, et al Insulinoma-associated 1 has a panneurogenic role and promotes the generation and expansion of basal progenitors in the developing mouse neocortex. Neuron. 2008;60(1):40–55. 10.1016/j.neuron.2008.09.020 18940587

[64] Reillo I, Borrell V. Germinal zones in the developing cerebral cortex of ferret: ontogeny, cell cycle kinetics, and diversity of progenitors. Cereb Cortex. 2012;22(9):2039–2054. 10.1093/cercor/bhr284 21988826

[65] Fei JF, Haffner C, Huttner WB. 3' UTR-dependent, miR-92-mediated restriction of Tis21 expression maintains asymmetric neural stem cell division to ensure proper neocortex size. Cell Rep. 2014;7(2):398–411. 10.1016/j.celrep.2014.03.033 24726360

[66] Kamei Y, Inagaki N, Nishizawa M, Tsutsumi O, Taketani Y, Inagaki M. Visualization of mitotic radial glial lineage cells in the developing rat brain by Cdc2 kinase-phosphorylated vimentin. Glia. 1998;23(3):191–199. 9633804 10.1002/(sici)1098-1136(199807)23:3<191::aid-glia2>3.0.co;2-8

[67] Mora-Bermudez F, Matsuzaki F, Huttner WB. Specific polar subpopulations of astral microtubules control spindle orientation and symmetric neural stem cell division. Elife. 2014;3. 10.7554/eLife.02875 PMC4112548 24996848

[68] Kosodo Y, Röper K, Haubensak W, Marzesco A-M, Corbeil D, Huttner WB. Asymmetric distribution of the apical plasma membrane during neurogenic divisions of mammalian neuroepithelial cells. EMBO J. 2004;23:2314–2324. 15141162 10.1038/sj.emboj.7600223 PMC419905

[69] Molyneaux BJ, Arlotta P, Menezes JR, Macklis JD. Neuronal subtype specification in the cerebral cortex. Nat Rev Neurosci. 2007;8(6):427–437. 17514196 10.1038/nrn2151

[70] Molnar Z, Clowry G. Cerebral cortical development in rodents and primates. Prog Brain Res. 2012;195:45–70. 10.1016/B978-0-444-53860-4.00003-9 22230622

[71] Manuel M, Georgala PA, Carr CB, Chanas S, Kleinjan DA, Martynoga B, et al Controlled overexpression of Pax6 in vivo negatively autoregulates the Pax6 locus, causing cell-autonomous defects of late cortical progenitor proliferation with little effect on cortical arealization. Development. 2007;134(3):545–555. 17202185 10.1242/dev.02764 PMC2386558

[72] Fish JL, Kosodo Y, Enard W, Paabo S, Huttner WB. Aspm specifically maintains symmetric proliferative divisions of neuroepithelial cells. Proc Natl Acad Sci USA. 2006;103(27):10438–10443. 16798874 10.1073/pnas.0604066103 PMC1502476

[73] Postiglione MP, Juschke C, Xie Y, Haas GA, Charalambous C, Knoblich JA. Mouse inscuteable induces apical-basal spindle orientation to facilitate intermediate progenitor generation in the developing neocortex. Neuron. 2011;72(2):269–284. 10.1016/j.neuron.2011.09.022 22017987 PMC3199734

[74] Walcher T, Xie Q, Sun J, Irmler M, Beckers J, Ozturk T, et al Functional dissection of the paired domain of Pax6 reveals molecular mechanisms of coordinating neurogenesis and proliferation. Development. 2013;140(5):1123–1136. 10.1242/dev.082875 23404109 PMC3583046

[75] Lancaster MA, Renner M, Martin CA, Wenzel D, Bicknell LS, Hurles ME, et al Cerebral organoids model human brain development and microcephaly. Nature. 2013;501(7467):373–379. 10.1038/nature12517 23995685 PMC3817409

[76] Xu ZP, Saunders GF. Transcriptional regulation of the human PAX6 gene promoter. J Biol Chem. 1997;272(6):3430–3436. 9013587 10.1074/jbc.272.6.3430

[77] Xu PX, Zhang X, Heaney S, Yoon A, Michelson AM, Maas RL. Regulation of Pax6 expression is conserved between mice and flies. Development. 1999;126(2):383–395. 9847251 10.1242/dev.126.2.383

[78] Morgan R. Conservation of sequence and function in the Pax6 regulatory elements. Trends Genet. 2004;20(7):283–287. 15219391 10.1016/j.tig.2004.04.009

[79] Kleinjan DA, Seawright A, Mella S, Carr CB, Tyas DA, Simpson TI, et al Long-range downstream enhancers are essential for Pax6 expression. Dev Biol. 2006;299(2):563–581. 17014839 10.1016/j.ydbio.2006.08.060 PMC2386664

[80] Tyas DA, Simpson TI, Carr CB, Kleinjan DA, van Heyningen V, Mason JO, et al Functional conservation of Pax6 regulatory elements in humans and mice demonstrated with a novel transgenic reporter mouse. BMC Dev Biol. 2006;6:21 16674807 10.1186/1471-213X-6-21 PMC1464123

[81] Elso C, Lu X, Weisner PA, Thompson HL, Skinner A, Carver E, et al A reciprocal translocation dissects roles of Pax6 alternative promoters and upstream regulatory elements in the development of pancreas, brain, and eye. Genesis. 2013;51(9):630–646. 10.1002/dvg.22409 23798316

[82] Bhatia S, Bengani H, Fish M, Brown A, Divizia MT, de Marco R, et al Disruption of autoregulatory feedback by a mutation in a remote, ultraconserved PAX6 enhancer causes aniridia. Am J Hum Genet. 2013;93(6):1126–1134. 10.1016/j.ajhg.2013.10.028 24290376 PMC3852925

[83] Needhamsen M, White RB, Giles KM, Dunlop SA, Thomas MG. Regulation of human PAX6 expression by miR-7. Evol Bioinform Online. 2014;10:107–113. 10.4137/EBO.S13739 25089088 PMC4116382

[84] Pevny LH, Nicolis SK. Sox2 roles in neural stem cells. Int J Biochem Cell Biol. 2010;42(3):421–424. 10.1016/j.biocel.2009.08.018 19733254

[85] Wen J, Hu Q, Li M, Wang S, Zhang L, Chen Y, et al Pax6 directly modulate Sox2 expression in the neural progenitor cells. Neuroreport. 2008;19(4):413–417. 10.1097/WNR.0b013e3282f64377 18287938

[86] Marthiens V, Kazanis I, Moss L, Long K, ffrench-Constant C. Adhesion molecules in the stem cell niche—more than just staying in shape? J Cell Sci. 2010;123(Pt 10):1613–1622. 20445012 10.1242/jcs.054312 PMC2864709

[87] von Holst A, Egbers U, Prochiantz A, Faissner A. Neural stem/progenitor cells express 20 Tenascin C isoforms that are differentially regulated by Pax6. J Biol Chem. 2007;282(12):9172–9181. 17264084 10.1074/jbc.M608067200

[88] Duncan MK, Kozmik Z, Cveklova K, Piatigorsky J, Cvekl A. Overexpression of PAX6(5a) in lens fiber cells results in cataract and upregulation of (alpha)5(beta)1 integrin expression. J Cell Sci. 2000;113 (Pt 18):3173–3185. 10954416 10.1242/jcs.113.18.3173

[89] Stahl R, Walcher T, De Juan Romero C, Pilz GA, Cappello S, Irmler M, et al Trnp1 regulates expansion and folding of the Mammalian cerebral cortex by control of radial glial fate. Cell. 2013;153(3):535–549. 10.1016/j.cell.2013.03.027 23622239

[90] Florio M, Albert M, Taverna E, Namba T, Brandl H, Lewitus E, et al Human-specific gene ARHGAP11B promotes basal progenitor amplification and neocortex expansion. Science. 2015;347(6229):1465–1470. 10.1126/science.aaa1975 25721503

[91] Nonaka-Kinoshita M, Reillo I, Artegiani B, Martinez-Martinez MA, Nelson M, Borrell V, et al Regulation of cerebral cortex size and folding by expansion of basal progenitors. EMBO J. 2013;32(13):1817–1828. 10.1038/emboj.2013.96 23624932 PMC3926188

[92] Lui JH, Nowakowski TJ, Pollen AA, Javaherian A, Kriegstein AR, Oldham MC. Radial glia require PDGFD-PDGFRbeta signalling in human but not mouse neocortex. Nature. 2014;515(7526):264–268. 10.1038/nature13973 25391964 PMC4231536

[93] Enard W, Przeworski M, Fisher SE, Lai CS, Wiebe V, Kitano T, et al Molecular evolution of FOXP2, a gene involved in speech and language. Nature. 2002;418(6900):869–872. 12192408 10.1038/nature01025

[94] Prüfer K, Racimo F, Patterson N, Jay F, Sankararaman S, Sawyer S, et al The complete genome sequence of a Neanderthal from the Altai Mountains. Nature. 2014;505(7481):43–49. 10.1038/nature12886 24352235 PMC4031459

[95] Rodriguez CI, Buchholz F, Galloway J, Sequerra R, Kasper J, Ayala R, et al High-efficiency deleter mice show that FLPe is an alternative to Cre-loxP. Nat Genet. 2000;25(2):139–140. 10835623 10.1038/75973

[96] Fu J, Teucher M, Anastassiadis K, Skarnes W, Stewart AF. A recombineering pipeline to make conditional targeting constructs. Methods Enzymol. 2010;477:125–144. 10.1016/S0076-6879(10)77008-7 20699140

[97] Saito K, Dubreuil V, Arai Y, Wilsch-Brauninger M, Schwudke D, Saher G, et al Ablation of cholesterol biosynthesis in neural stem cells increases their VEGF expression and angiogenesis but causes neuron apoptosis. Proc Natl Acad Sci USA. 2009;106(20):8350–8355. 10.1073/pnas.0903541106 19416849 PMC2688855

[98] Lange C, Huttner WB, Calegari F. Cdk4/cyclinD1 overexpression in neural stem cells shortens G1, delays neurogenesis, and promotes the generation and expansion of basal progenitors. Cell Stem Cell. 2009;5(3):320–331. 10.1016/j.stem.2009.05.026 19733543

[99] Niwa H, Yamamura K, Miyazaki J. Efficient selection for high-expression transfectants with a novel eukaryotic vector. Gene. 1991;108(2):193–199. 1660837 10.1016/0378-1119(91)90434-d

[100] De Pietri Tonelli D, Calegari F, Fei JF, Nomura T, Osumi N, Heisenberg CP, et al Single-cell detection of microRNAs in developing vertebrate embryos after acute administration of a dual-fluorescence reporter/sensor plasmid. BioTechniques. 2006;41(6):727–732. 17191618 10.2144/000112296

[101] Kranz A, Fu J, Duerschke K, Weidlich S, Naumann R, Stewart AF, et al An improved Flp deleter mouse in C57Bl/6 based on Flpo recombinase. Genesis. 2010;48(8):512–520. 10.1002/dvg.20641 20506501

[102] Takahashi M, Sato K, Nomura T, Osumi N. Manipulating gene expressions by electroporation in the developing brain of mammalian embryos. Differentiation. 2002;70(4–5):155–162. 12147135 10.1046/j.1432-0436.2002.700405.x

[103] Packard DS Jr., Menzies RA, Skalko RG. Incorportaiton of thymidine and its analogue, bromodeoxyuridine, into embryos and maternal tissues of the mouse. Differentiation. 1973;1(6):397–404. 4802502 10.1111/j.1432-0436.1973.tb00137.x

[104] Pilaz LJ, Patti D, Marcy G, Ollier E, Pfister S, Douglas RJ, et al Forced G1-phase reduction alters mode of division, neuron number, and laminar phenotype in the cerebral cortex. Proc Natl Acad Sci USA. 2009;106(51):21924–21929. 10.1073/pnas.0909894106 19959663 PMC2788480

[105] Pulvers JN, Huttner WB. Brca1 is required for embryonic development of the mouse cerebral cortex to normal size by preventing apoptosis of early neural progenitors. Development. 2009;136(11):1859–1868. 10.1242/dev.033498 19403657

[106] Burgess A, Vigneron S, Brioudes E, Labbe JC, Lorca T, Castro A. Loss of human Greatwall results in G2 arrest and multiple mitotic defects due to deregulation of the cyclin B-Cdc2/PP2A balance. Proc Natl Acad Sci USA. 2010;107(28):12564–12569. 10.1073/pnas.0914191107 20538976 PMC2906566

