# Supplementary figures and images for: Sustained Pax6 Expression Generates Primate-like Basal Radial Glia in Developing Mouse Neocortex
Source: PLoS Biol. 2015 Aug 7;13(8):e1002217. doi: 10.1371/journal.pbio.1002217 (PMC4529158; doi:10.1371/journal.pbio.1002217)

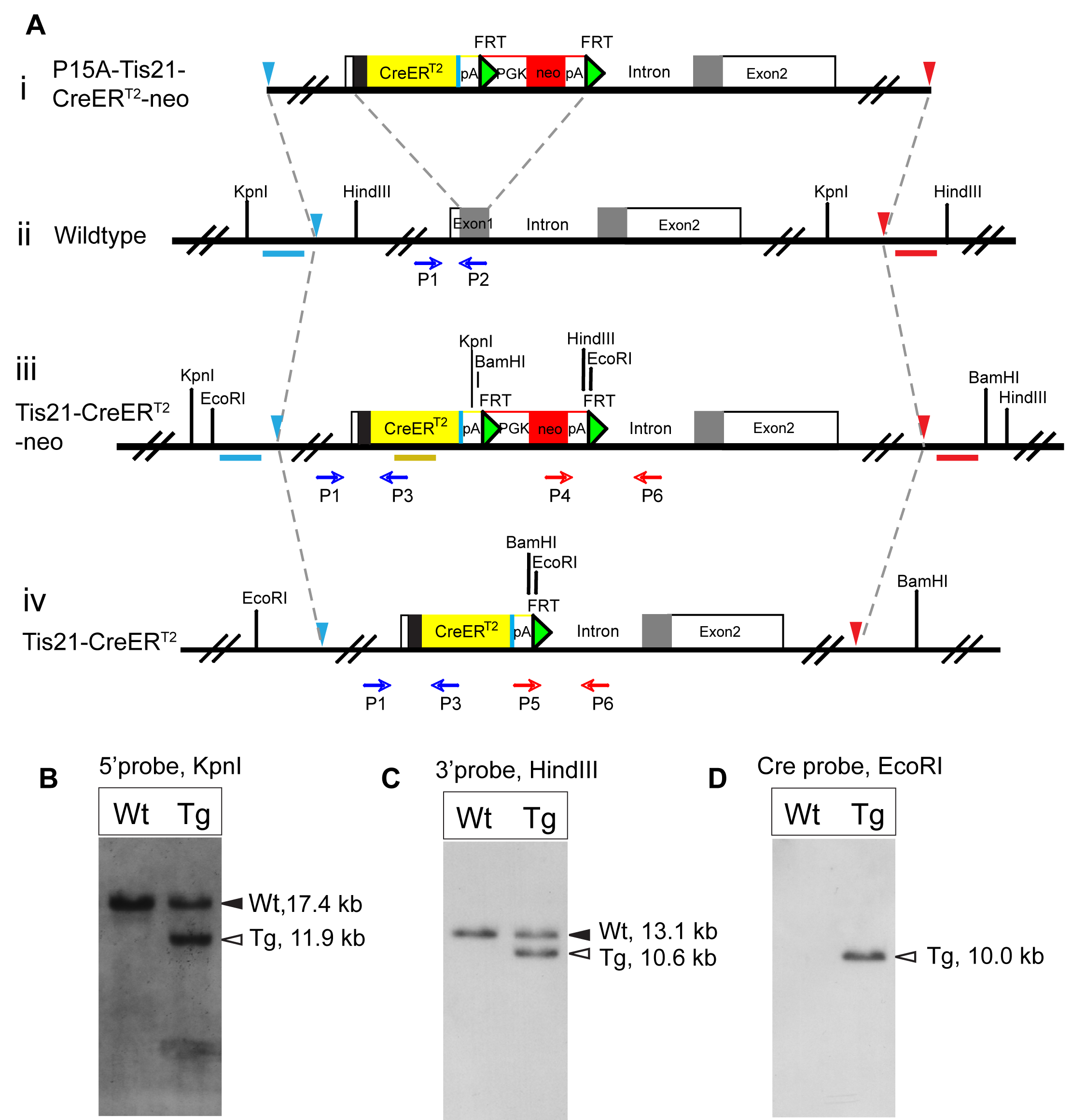

Supplement: S1 Fig — (A) Generation of the Tis21-CreERT2 allele by homologous recombination. (i–iii) Steps carried out with ES cells. Using the targeting construct shown (i), the coding sequence in Exon1 of Tis21 (shaded box, ii) was replaced by a CreERT2 cassette (yellow box, i) followed by a neomycin cassette (neo, red box, i) flanked by FRT sites (green triangles, i), yielding the Tis21-CreERT2-neo allele (iii). (iii–iv) Steps carried out with mice. Removal of the neomycin cassette by crossing mice carrying the Tis21-CreERT2-neo allele (iii) with transgenic hACTB-FLPe mice expressing FLPe recombinase, yielding mice carrying the Tis21-CreERT2 allele (iv). (i–iv) Shaded boxes, Tis21 ORF; blue and red triangles, 5’ and 3’ ends, respectively, of homology between the targeting construct (i) and the Tis21 wildtype allele (ii); P1–P6, primers used for genotyping PCR; blue, yellow, and red bars, location of probes used for Southern blot analyses. For details, see Experimental Procedures. (B–D) Southern blot analysis of genomic DNA from wildtype (Wt) and transgenic Tis21-CreERT2-neo heterozygous (Tg) embryonic stem cells. DNA was digested with KpnI (B), HindIII (C) or EcoRI (D), and hybridized with either the 5’ probe, the 3’ probe, or the Cre probe, respectively, as indicated by the blue, red, or yellow bars in (A, ii, and iii). Solid arrowheads in (B), 17.4 kb fragment of KpnI-digested wt allele (A, ii); in (C), 13.1 kb fragment of HindIII-digested wt allele (A, ii); open arrowheads in (B), 11.9 kb fragment of KpnI-digested Tis21-CreERT2-neo allele (A, iii); in (C), 10.6 kb fragment of HindIII digested Tis21-CreERT2-neo allele (A, iii); in (D), 10.0 kb fragment of EcoRI digested Tis21-CreERT2-neo allele (A, iii). (TIF) [file pbio.1002217.s002.tif]

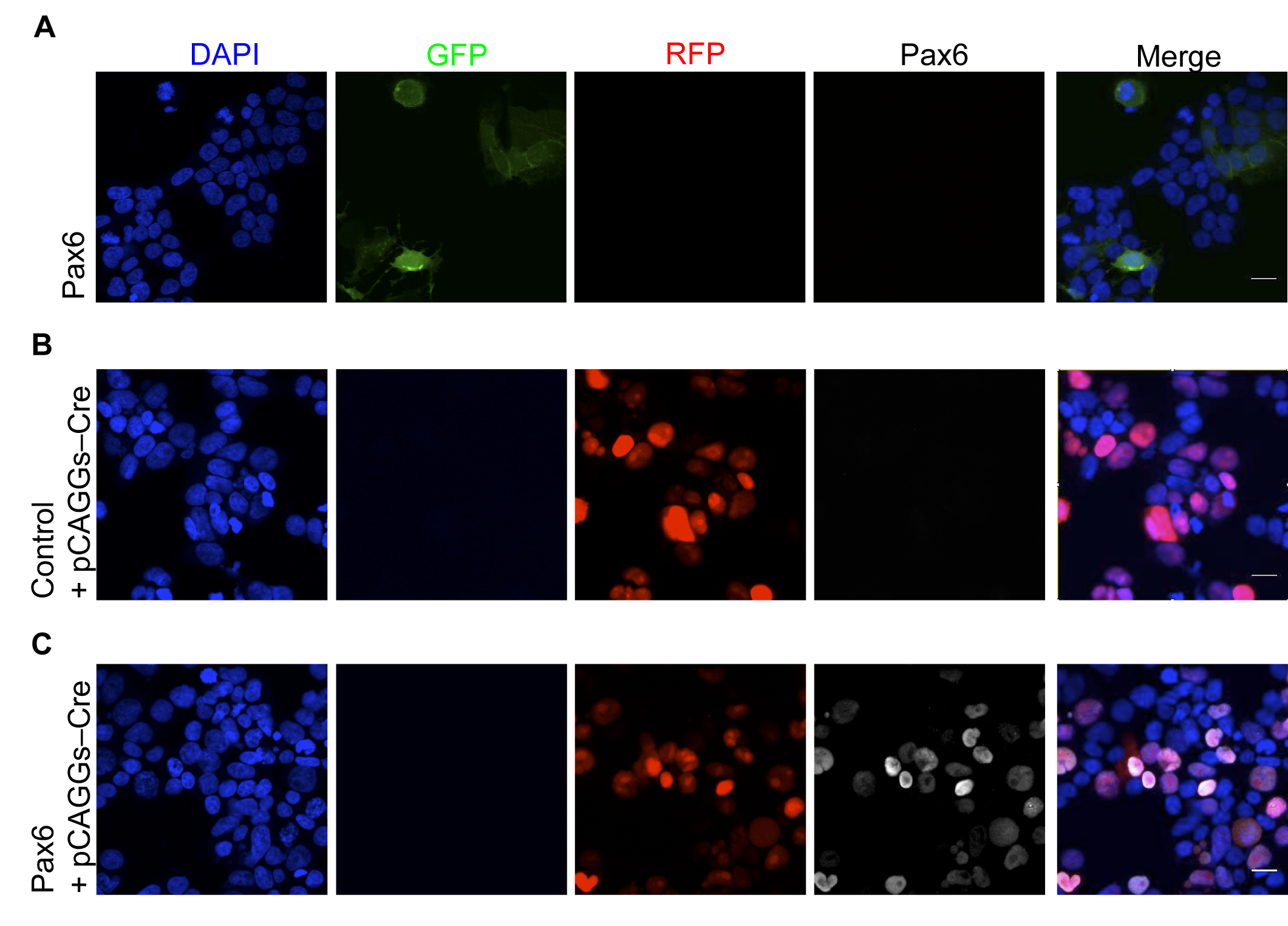

Supplement: S2 Fig — HEK293T cells were transfected with Pax6-expressing plasmid (A), control plasmid plus pCAGGs-Cre (B), or Pax6-expressing plasmid plus pCAGGs-Cre (C), followed 48 h later by Pax6 immunofluorescence (white) and GFP (green) and RFP (red) fluorescence, combined with DAPI staining (blue). Scale bars, 20 μm. (TIF) [file pbio.1002217.s003.tif]

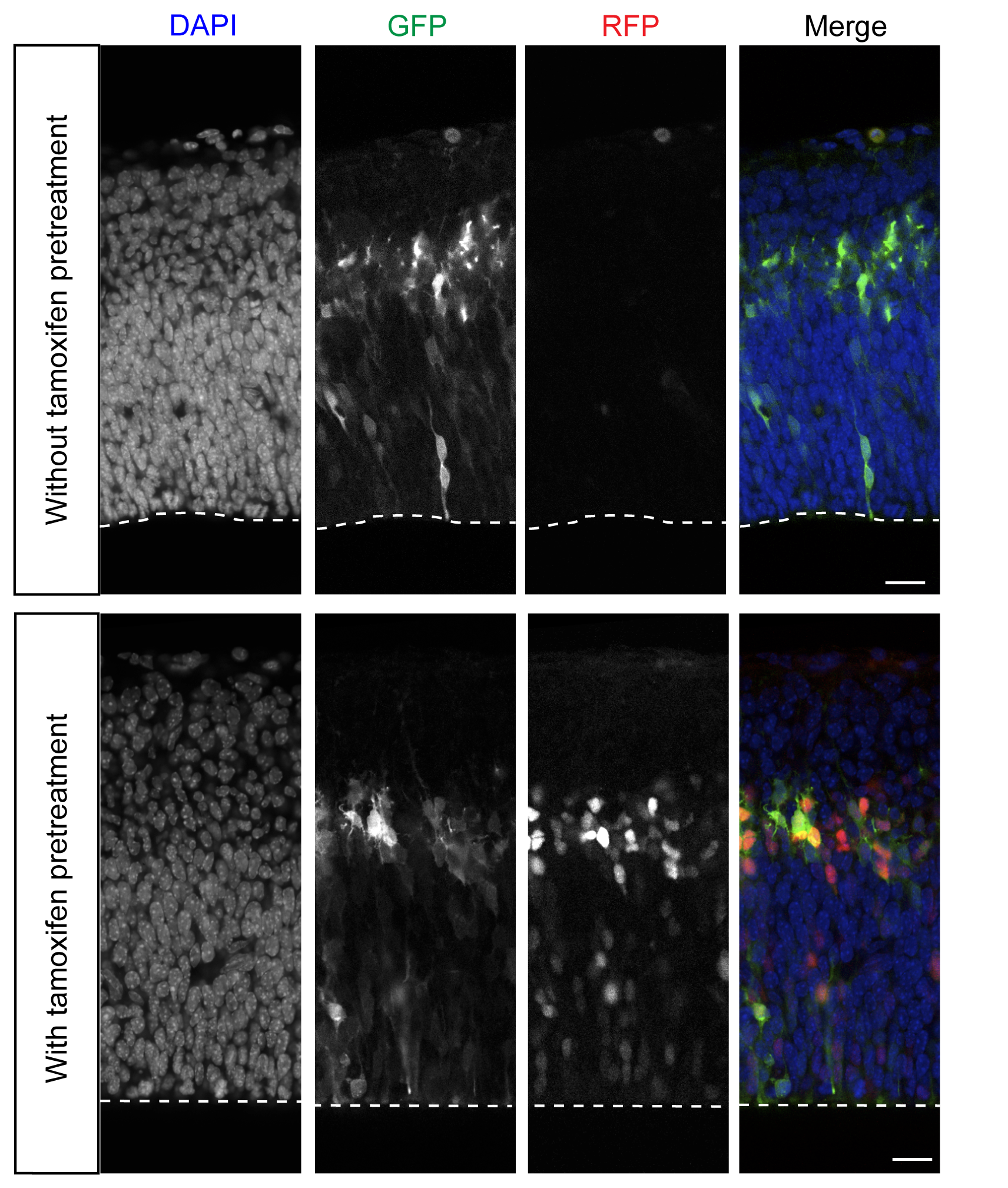

Supplement: S3 Fig — Dorsolateral telencephalon of E14.5 Tis21–CreERT2 heterozygous mice electroporated at E13.5 with Pax6-expressing plasmid without (top) or with (bottom) tamoxifen pretreatment (see Fig 2B). GFP (green) and RFP (red) fluorescence, combined with DAPI staining (blue), on coronal 50-μm vibratome sections. Scale bars, 20 μm. (TIF) [file pbio.1002217.s004.tif]

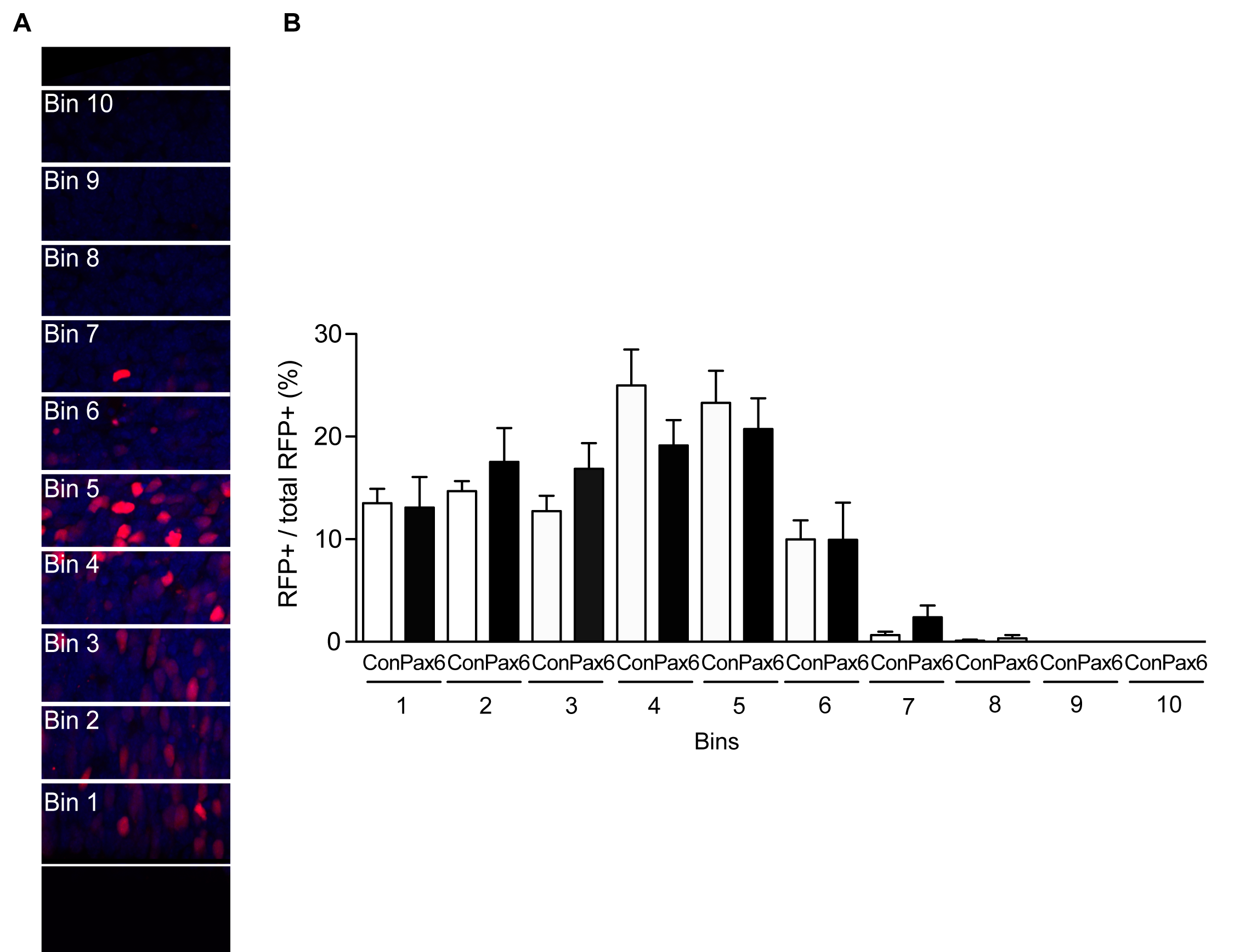

Supplement: S4 Fig — Dorsolateral telencephalon of tamoxifen-treated E14.5 Tis21–CreERT2 heterozygous mice electroporated at E13.5 with Pax6-expressing (A,B) or control (B) plasmid (see Fig 2B). (A) Representative example of the distribution of RFP-positive cells (red) in the cortical wall, divided into ten equally sized bins, with the bin containing the ventricular surface being defined as bin 1. Blue, DAPI staining; coronal 50-μm vibratome sections. (B) Quantification of the distribution of RFP-positive cells across the ten bins (see A), expressed as percentage of all RFP-positive cells in the cortical wall (200-μm wide area), upon control (Con, white) and Pax6 (black) electroporation. Mean of three independent experiments, each being the average of two to four embryos. Error bars, SEM. (TIF) [file pbio.1002217.s005.tif]

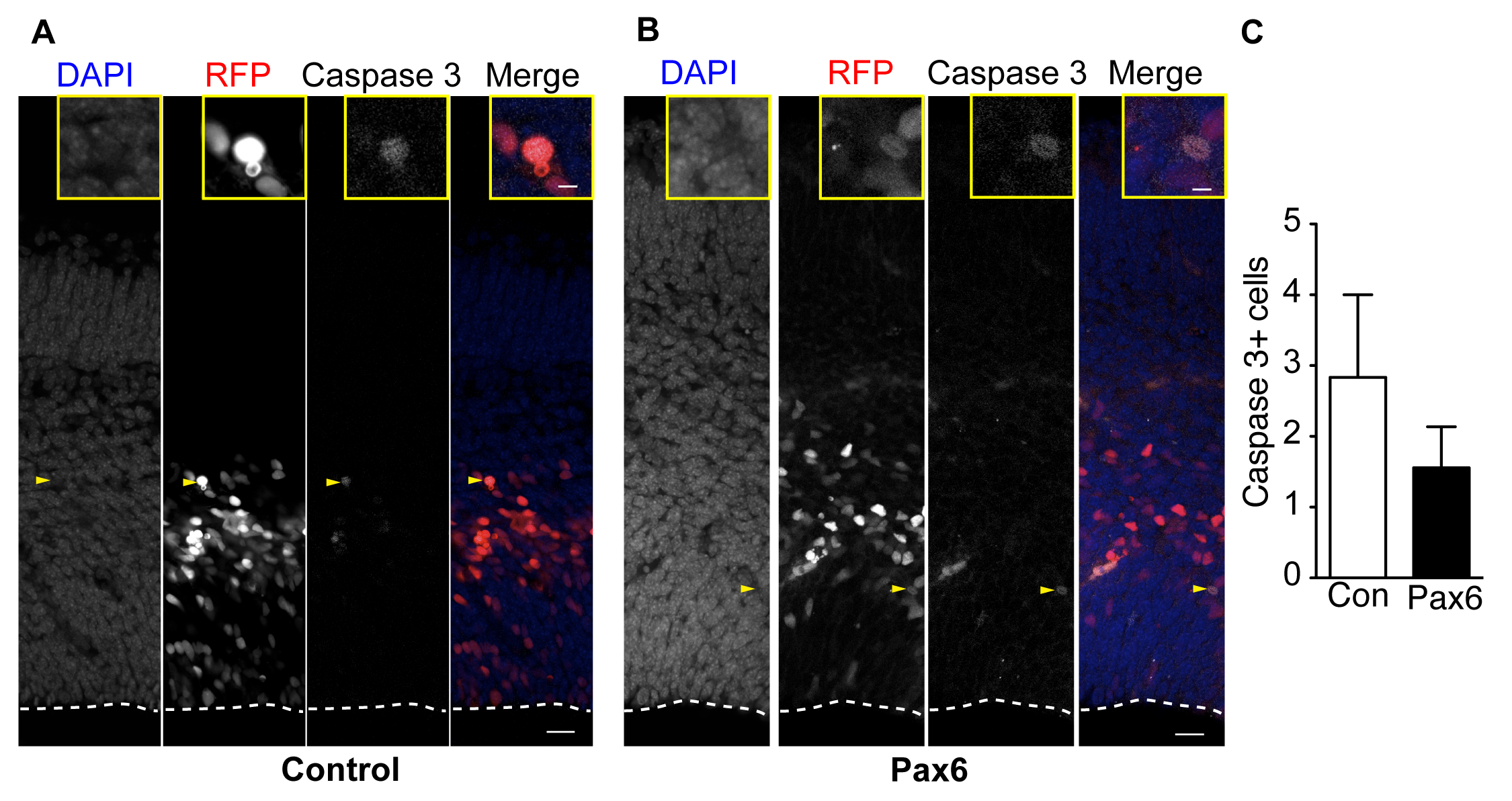

Supplement: S5 Fig — Dorsolateral telencephalon of tamoxifen-treated E14.5 Tis21–CreERT2 heterozygous mice electroporated at E13.5 with control (A,C) or Pax6-expressing (B,C) plasmid (see Fig 2B). (A,B) Caspase-3 immunofluorescence (white) and RFP fluorescence (red), combined with DAPI staining (blue), on coronal 50-μm vibratome sections. Dashed white lines, ventricular surface. Scale bars, 20 μm. (C) Quantification of caspase-3- and RFP-positive cells in the cortical wall (200-μm wide area), upon control (Con, white) and Pax6 (black) electroporation. Mean of three independent experiments, each being the average of two to four embryos. Error bars, SEM. (TIF) [file pbio.1002217.s006.tif]

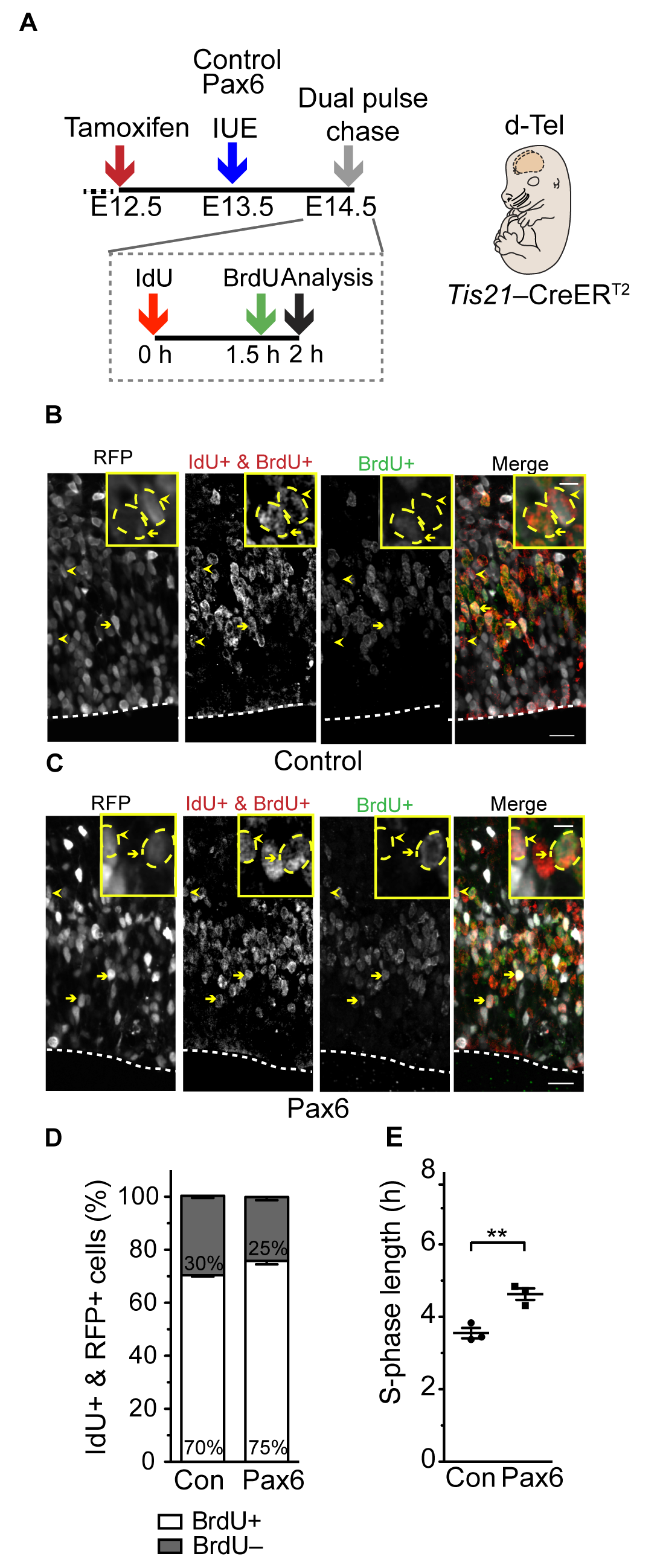

Supplement: S6 Fig — (A) Flow scheme of the experiment. (B–E) Dorsolateral telencephalon of tamoxifen-treated E14.5 Tis21–CreERT2 heterozygous mice electroporated at E13.5 with control (B,D,E) or Pax6-expressing (C–E) plasmid. IdU and BrdU were injected at 2 h and 0.5 h, respectively, before sacrifice. (B,C) RFP (white), IdU & BrdU (red), and BrdU only (green) immunofluorescence, on coronal 20-μm cryosections. Scale bars, 20 μm and 5 μm (insets). Yellow arrows, triple-positive cells (RFP+, IdU+, BrdU+); yellow arrowheads, double-positive cells (RFP+, IdU+, BrdU–); dashed white lines, ventricular surface. Insets show representative examples of RFP+ & IdU+ nuclei (outlined by dashed yellow lines) at higher magnification that are either BrdU+ (yellow arrows) or BrdU–(yellow arrowheads). (D) Quantification of RFP+, IdU+, & BrdU+ triple-positive cells (white) and RFP+, IdU+, and BrdU–double-positive cells (grey) in the cortical wall expressed as percentage of all cells that are both IdU+ & RFP+ in the cortical wall (200-μm wide area), upon control (Con) and Pax6 electroporation. (E) Quantification of S-phase length upon control (Con, circles) and Pax6 (squares) electroporation. Mean of three embryos from two independent experiments. Error bars, SEM. ** p < 0.01. (TIF) [file pbio.1002217.s007.tif]

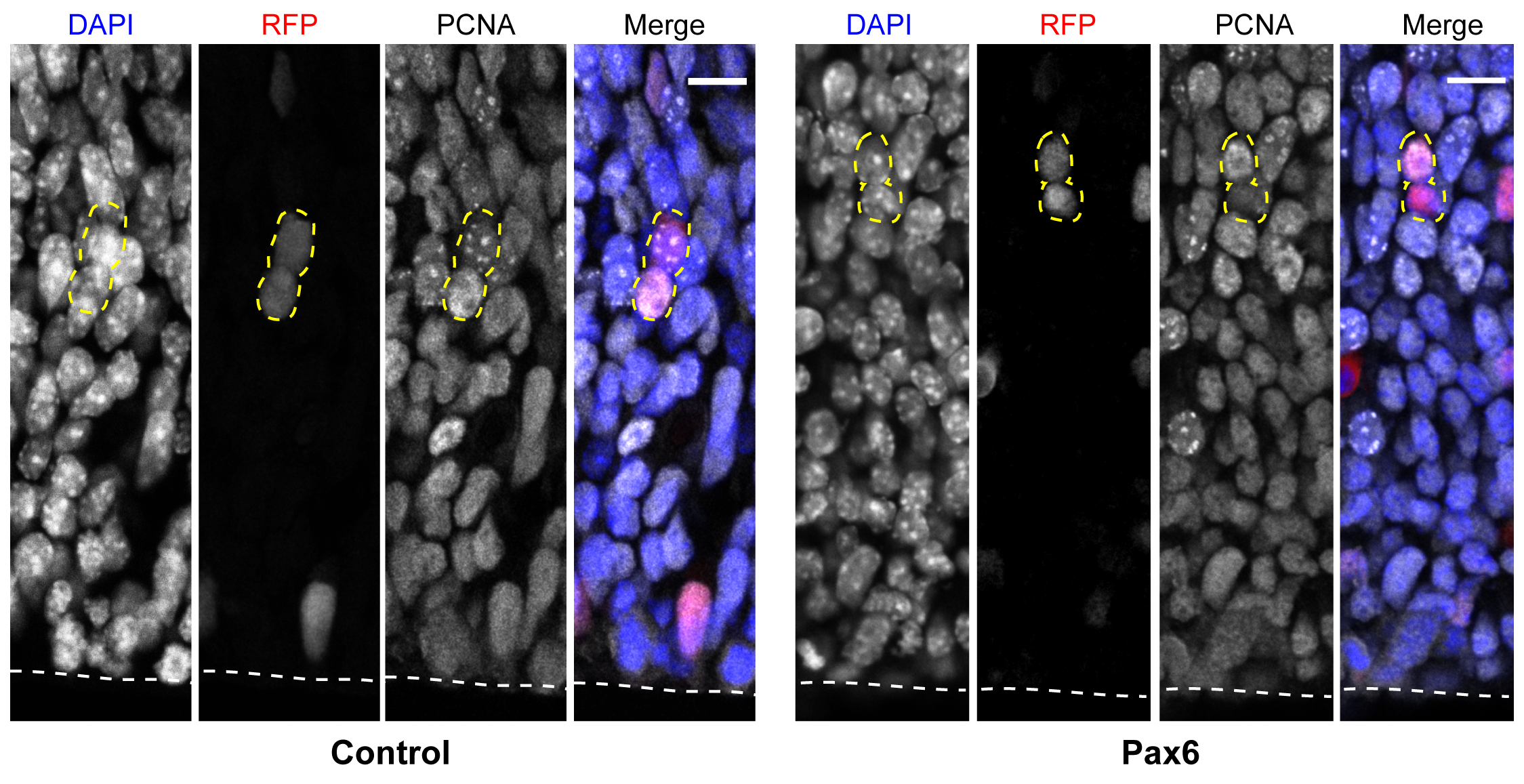

Supplement: S7 Fig — Dorsolateral telencephalon of tamoxifen-treated E14.5 Tis21–CreERT2 heterozygous mice electroporated at E13.5 with control (left) or Pax6-expressing (right) plasmid (see Fig 2B), showing representative examples of RFP+ (red) and PCNA+ (white) double-positive daughter cell pairs (dashed yellow lines) derived from Tis21-positive electroporated APs (12-μm cryosections). Note that all daughter cell pairs analyzed (10 pairs each for control and Pax6) were PCNA+, irrespective of the absence or presence of Tbr2 immunoreactivity. Dashed white lines, ventricular surface. Scale bars, 10 μm. (TIF) [file pbio.1002217.s008.tif]

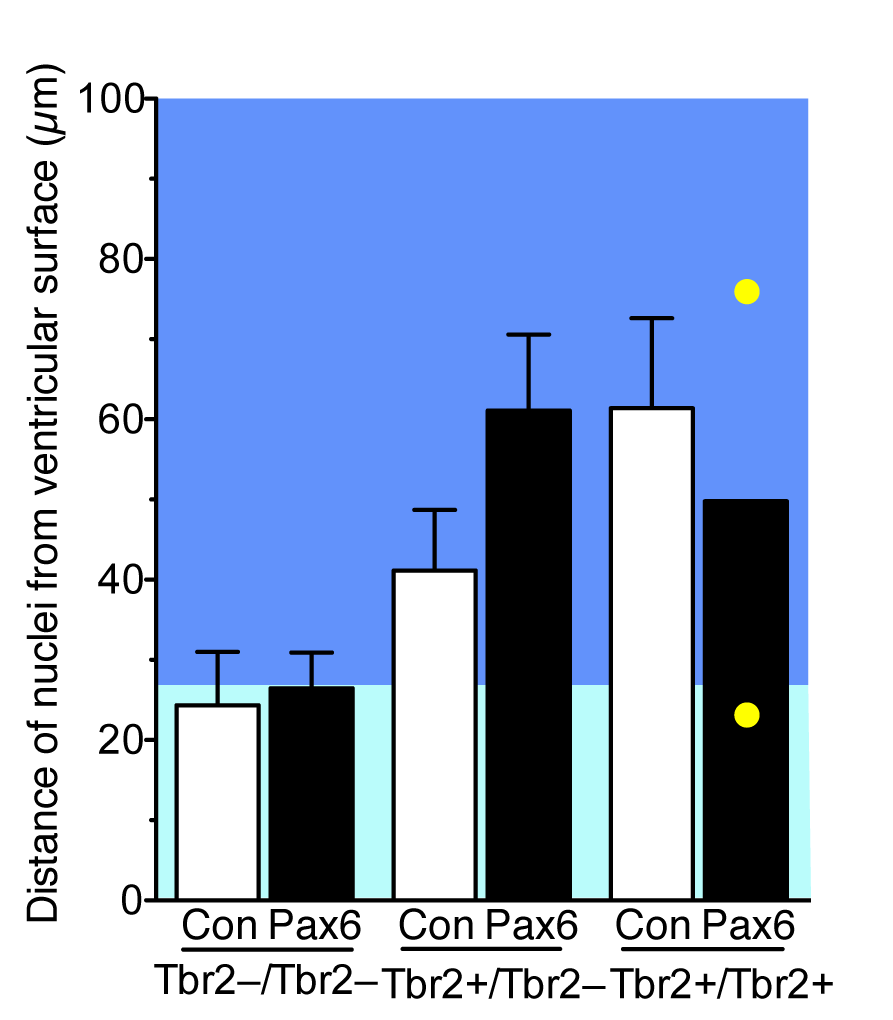

Supplement: S8 Fig — Distance of nuclei of the Tbr2–/Tbr2–, Tbr2+/Tbr2–, and Tbr2+/Tbr2+ daughter cell pairs from the ventricular surface upon control (Con, white) and Pax6 (black) electroporation. Data indicate the position of the ventricular-most nucleus of each pair (see Materials and Methods). Light and dark blue background indicates the areas within <27 μm and ≥27 μm from the ventricular surface, respectively. Mean of 2–15 cell pairs; error bars, SEM; yellow dots, individual values. (TIF) [file pbio.1002217.s009.tif]

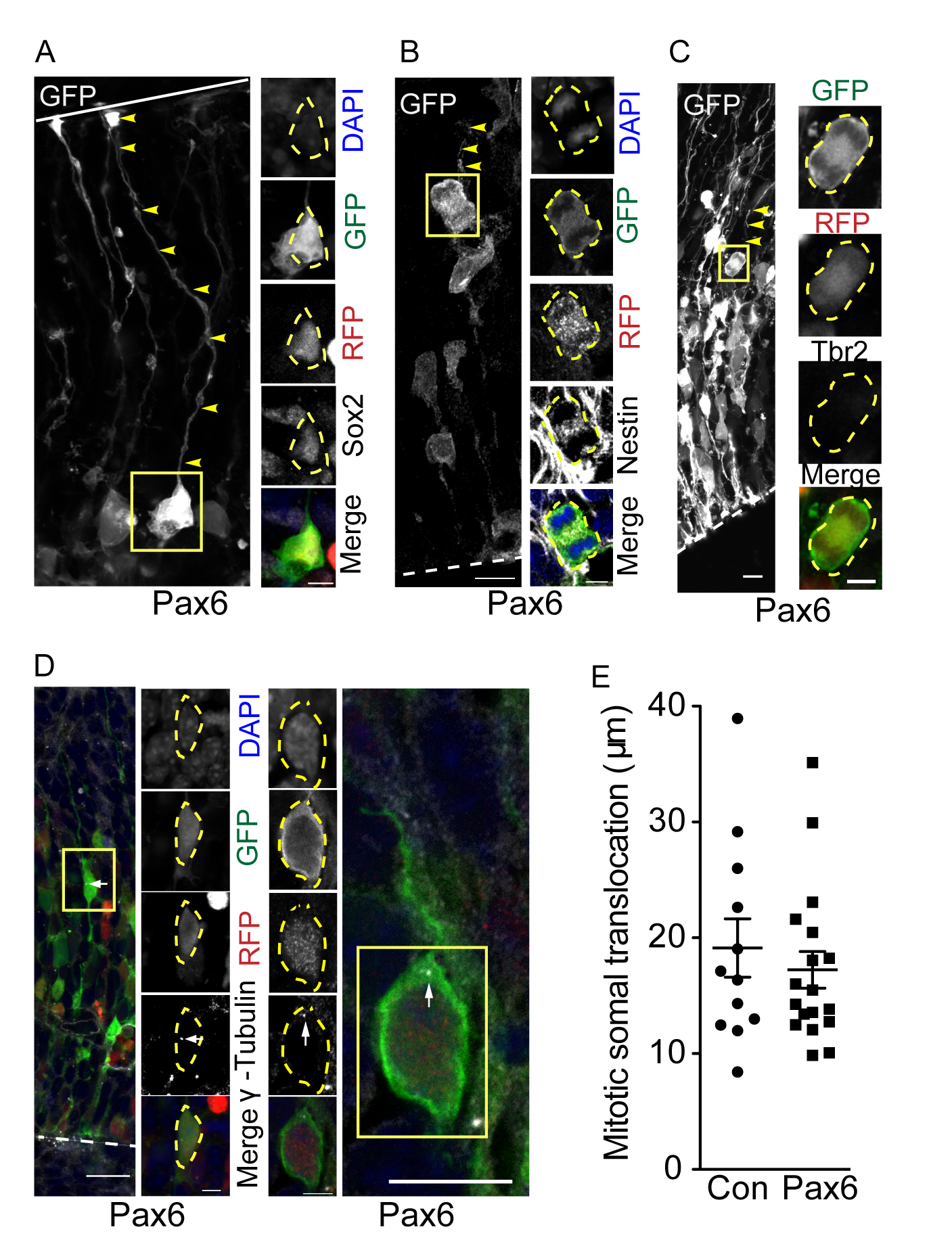

Supplement: S9 Fig — Dorsolateral telencephalon of tamoxifen-treated E14.5 Tis21–CreERT2 heterozygous mice electroporated at E13.5 with control (E) or Pax6-expressing (A–E) plasmid (see Fig 2B). (A–D) bRG identified by residual membrane-GFP fluorescence (maximum intensity projections of stacks of 5 (A), 9 (B), and 11 (C) images, single optical sections (D)). Yellow arrowheads, basal process; white arrow, centrosome location; yellow boxes indicate the cell body (yellow dashed lines) that is shown as single optical sections at higher magnification in the small panels; Sox2 (white, A), nestin (white, B), Tbr2 (white, C), and γ-tubulin (white, D) immunofluorescence, together with GFP (green) and RFP (red) fluorescence, combined with DAPI staining (A,B,D), on coronal 50-μm vibratome sections. Scale bars, 20 μm and 5 μm (small panels). (E) Quantification of basal mitotic somal translocation of bRG in control (Con, circles) and conditional Pax6 expression (Pax6, squares). Mean ± SEM. Control, 12 cells; Pax6, 20 cells. (TIF) [file pbio.1002217.s010.tif]

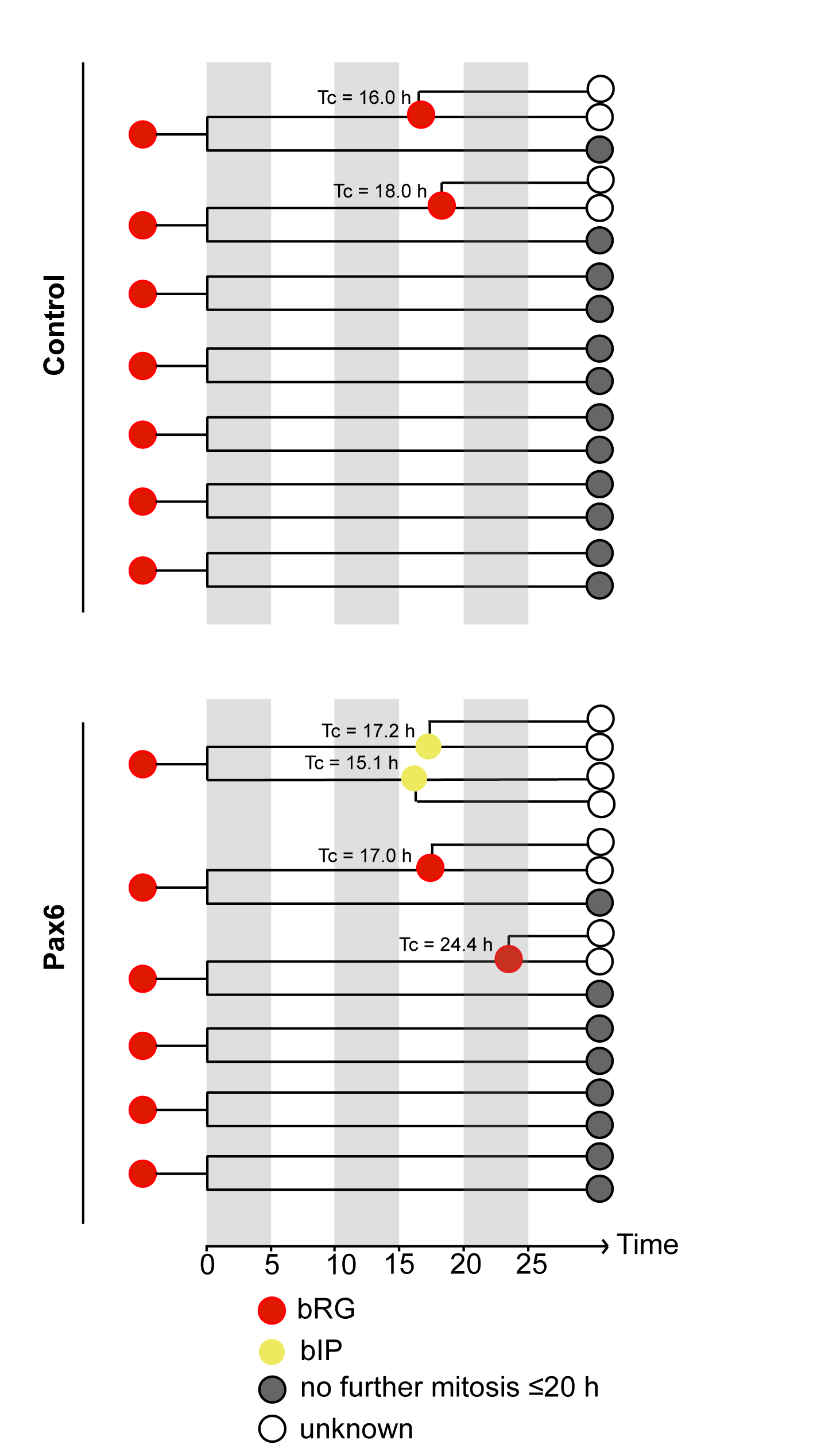

Supplement: S10 Fig — Summary of the 13 bRG-derived lineage trees observed upon live time-lapse imaging of E14.5 organotypic slices prepared from dorsolateral telencephalon of tamoxifen-treated Tis21–CreERT2 heterozygous mice electroporated with control or Pax6-expressing plasmid (see Fig 7). Control, 7 bRG divisions; Pax6, 6 bRG divisions. Tc, total cell cycle length; red circles, bRG; yellow circles, bIPs; grey circles, no further mitosis detected for progeny until 20 h; white circles, unknown cell type. (TIF) [file pbio.1002217.s011.tif]

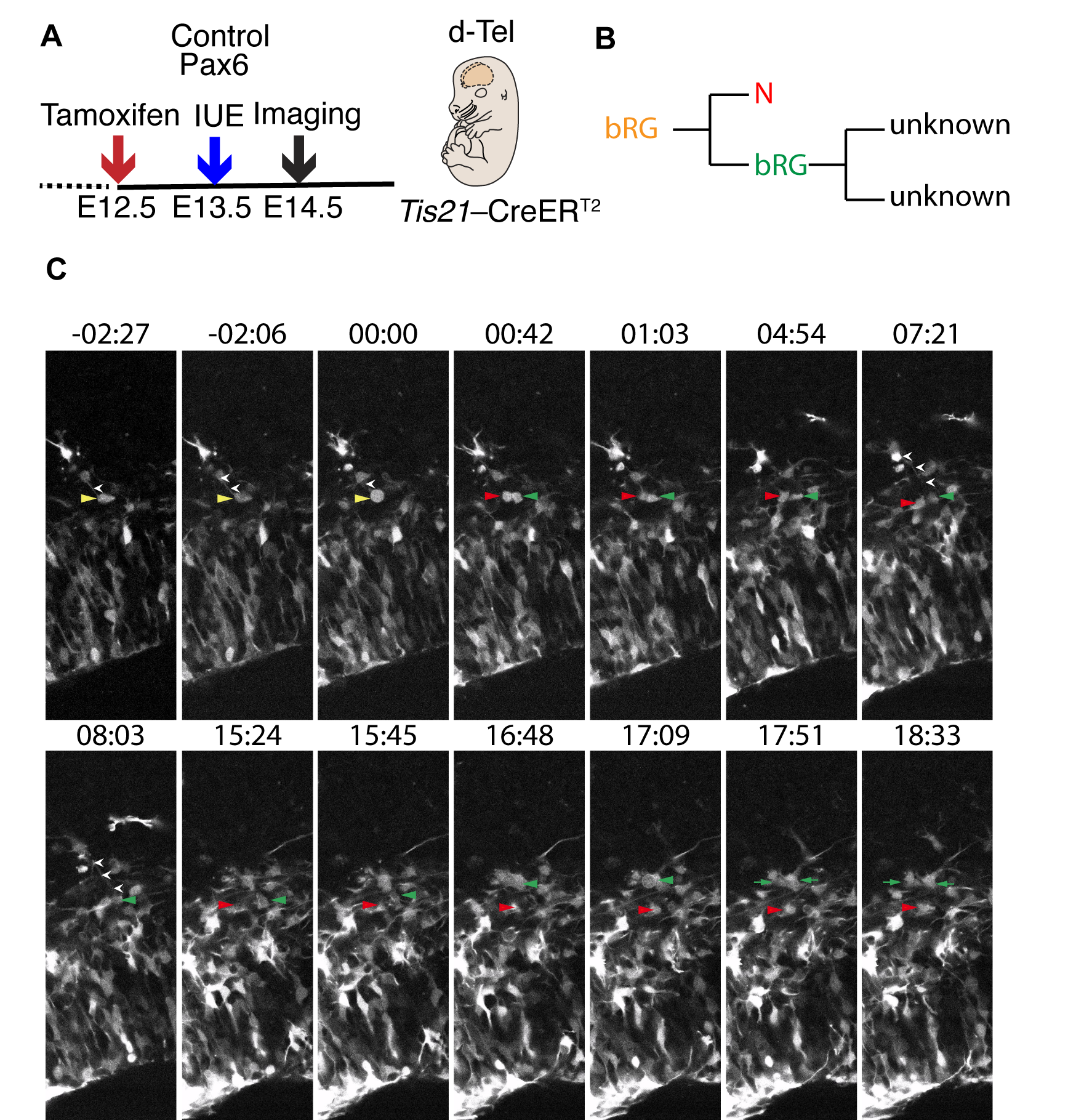

Supplement: S11 Fig — (A) Flow scheme of experiment. (B) Lineage tree reconstruction of bRG division. (C) Live time-lapse imaging of organotypic slice of dorsolateral telencephalon of tamoxifen-treated E14.5 Tis21–CreERT2 heterozygous mice electroporated at E13.5 with Pax6-expressing plasmid. Membrane-GFP fluorescence, single optical sections. 00:00 (hh:mm) denotes the start of mitosis. Yellow arrowheads, mother bRG; white arrowheads, basal process; green and red arrowheads, bRG daughter and neuron daughter, respectively, of mother bRG; green arrows, daughter cells of bRG daughter. (TIF) [file pbio.1002217.s012.tif]

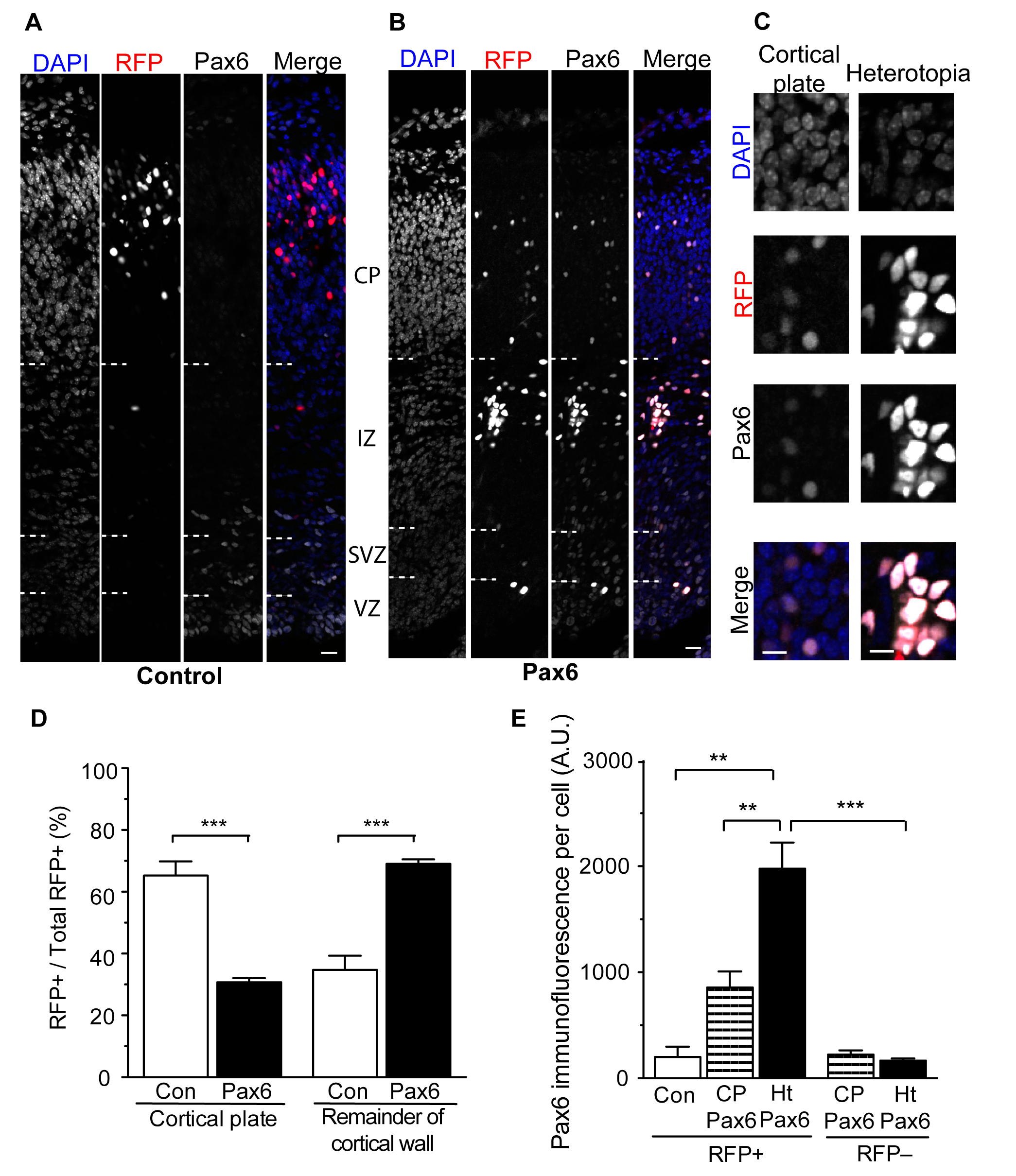

Supplement: S12 Fig — Dorsolateral telencephalon of tamoxifen-treated E17.5 Tis21–CreERT2 heterozygous embryos electroporated at E13.5 with control (A,D,E) or Pax6-expressing (B–E) plasmid subjected to a single EdU pulse (not illustrated) 10 h after electroporation (E14.0). (A–C) Pax6 immunofluorescence (white) and RFP fluorescence (red), combined with DAPI staining (blue), on coronal 50-μm vibratome sections. Images in (C) show representative examples at a higher magnification of RFP+ and Pax6+ double-positive progeny in the cortical plate (left) and exhibiting heterotopia in the intermediate zone (right); note the higher RFP and Pax6 (immuno)fluorescence level in the progeny exhibiting heterotopia. Scale bars, 20 μm. (D) Quantification of RFP+ nuclei in the cortical plate (left) and in the remainder of the cortical wall (right), expressed as percentage of all RFP-positive cells in the cortical wall (200-μm wide area), upon control (Con, white) and Pax6 (black) electroporation. Mean of eight embryos from at least two independent experiments. (E) Pax6 immunofluorescence intensity per cell (A.U., arbitrary units) in RFP-positive (RFP+) and-negative (RFP–) cells in cortical plate (CP, striped) and heterotopia (Ht, black) upon Pax6 electroporation, and in the cortical wall upon control (Con, white) electroporation. Mean of three independent experiments, each being the average of three embryos. (D,E) Error bars, SEM. ** p < 0.01, *** p < 0.001. (TIF) [file pbio.1002217.s013.tif]

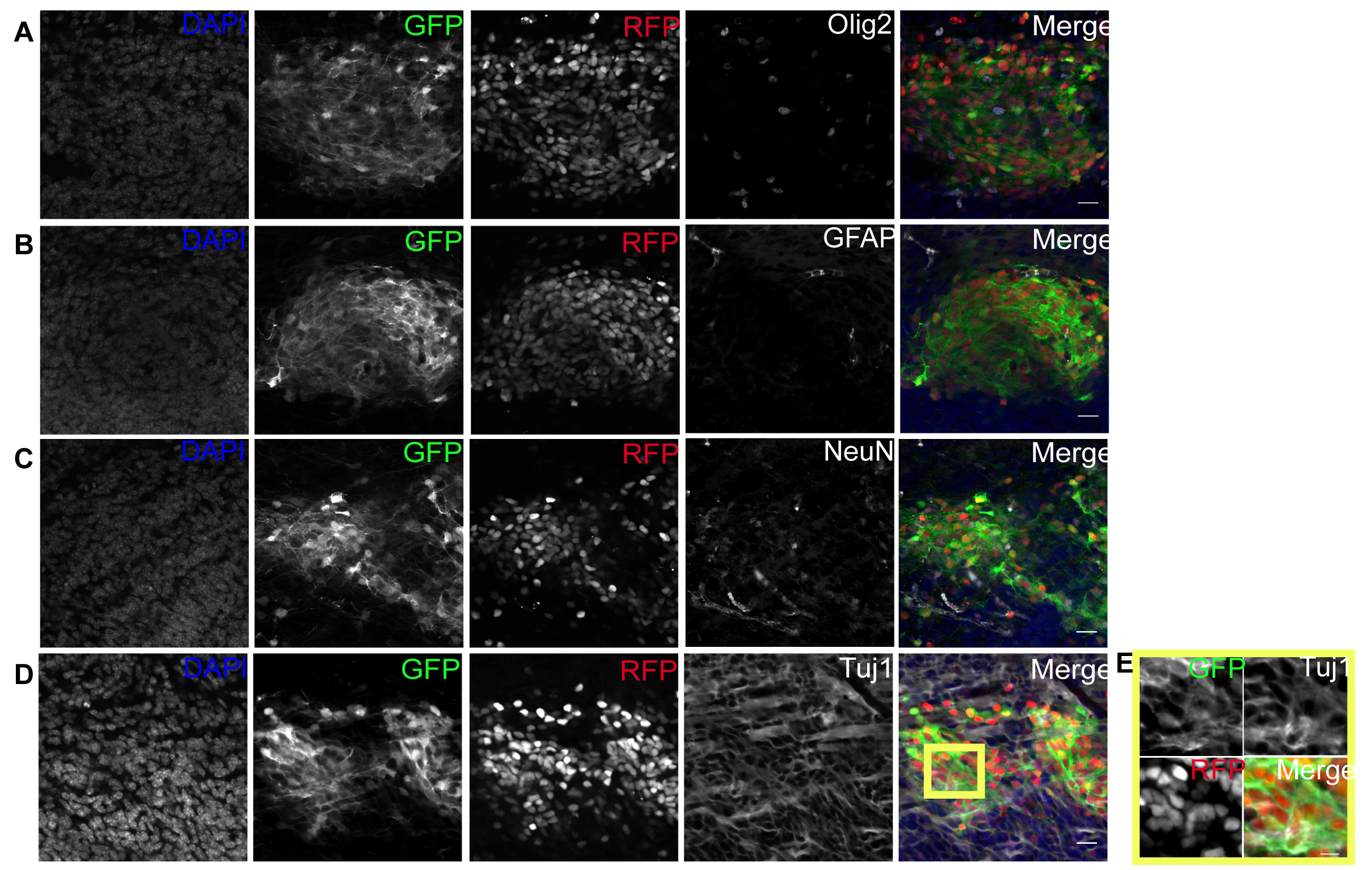

Supplement: S13 Fig — Dorsolateral telencephalon of tamoxifen-treated E17.5 Tis21–CreERT2 heterozygous embryos electroporated at E13.5 with Pax6 expressing plasmid and subjected to a single EdU pulse (not illustrated) 10 h after electroporation (E14.0), showing progeny exhibiting heterotopia. Olig2 (A), GFAP (B), NeuN (C), and Tuj1 (D) immunofluorescence (white), together with RFP (red) and GFP (green) fluorescence and DAPI staining (blue), on coronal 50-μm vibratome sections. The area indicated by the yellow box in (D) is shown at higher magnification in (E); note the colocalization of Tuj1 immunofluorescence and GFP fluorescence. Scale bars, 10 μm. (TIF) [file pbio.1002217.s014.tif]

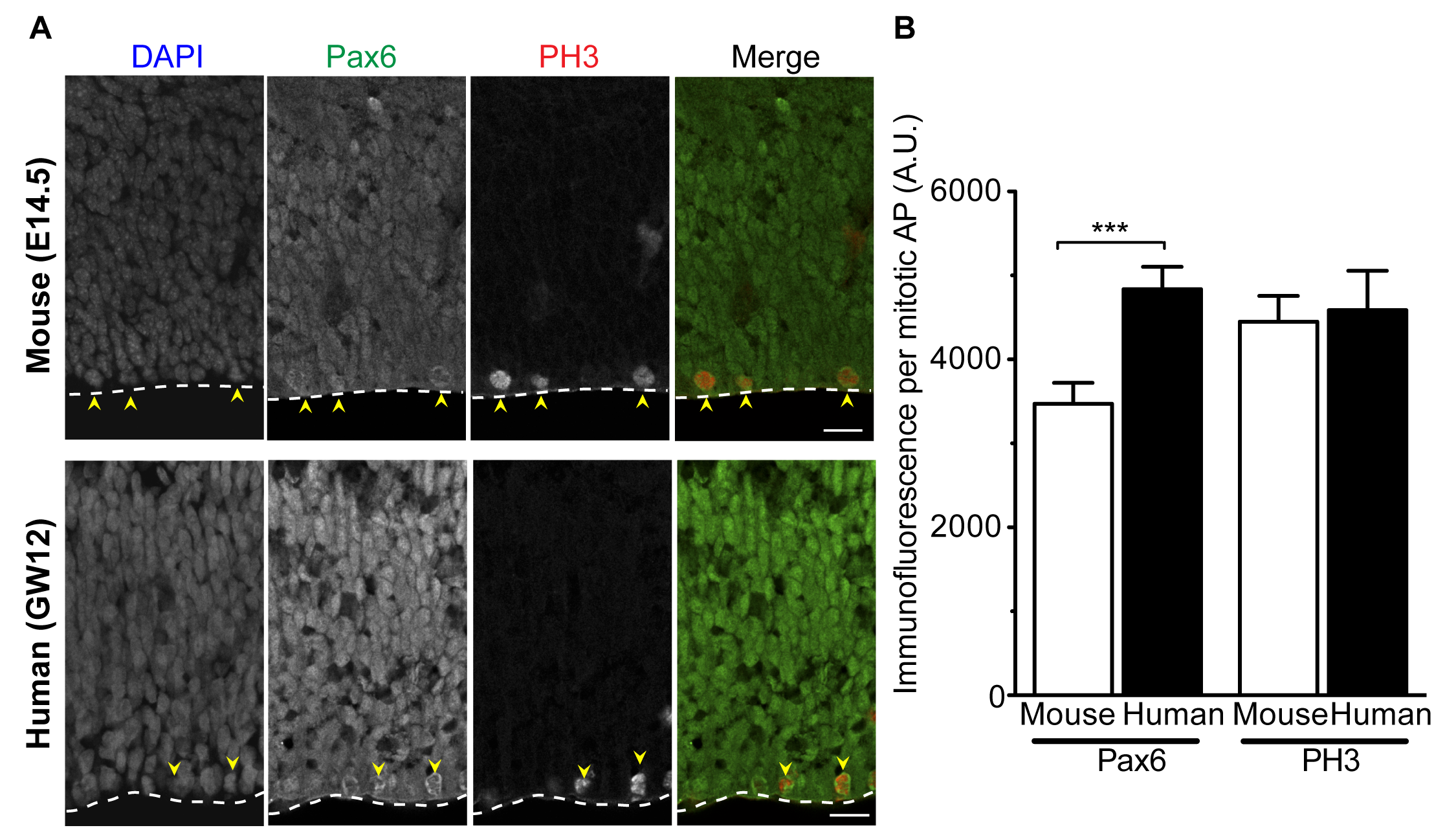

Supplement: S14 Fig — VZ of the rostral neocortex of wildtype E14.5 mouse (top) and gestational week (GW) 12 human was analyzed by double immunofluorescence for Pax6 and phosphohistone H3 (PH3). (A) Representative images showing Pax6 (green) and PH3 (red) immunofluorescence, combined with DAPI staining (blue), on coronal 12-μm cryosections. Dashed white lines, ventricular surface; yellow arrowheads, mitotic APs. Scale bars, 20 μm. (B) Quantification of Pax6 (left) and PH3 (right) immunofluorescence intensity per mitotic AP (A.U., arbitrary units). Note the higher Pax6 level in fetal human APs as compared to embryonic mouse APs, and the equal PH3 immunoreactivity level. Mean of 34 (mouse, white) and 32 (human, black) mitotic APs; error bars, SEM. *** p <0.001. (TIF) [file pbio.1002217.s015.tif]
