# Supplementary material for: Sustained Pax6 Expression Generates Primate-like Basal Radial Glia in Developing Mouse Neocortex
Source: PLoS Biol. 2015 Aug 7;13(8):e1002217. doi: 10.1371/journal.pbio.1002217 (PMC4529158; doi:10.1371/journal.pbio.1002217)
Supplement: S1 Table — (DOCX) [file pbio.1002217.s018.docx]

Cell cycle phases of Tis21+ aRG (h)

|  | Tc (Imaging)^a^ | Ts (PCNA) | T_M_ (Imaging)^a^ |
| --- | --- | --- | --- |
| Control | 21.0 | 2.4 | ≤1.0 |
| Pax6 | 18.5 | 3.0 | ≤1.0 |

Proportion of Tis21+ aRG in cell cycle phases (%)

|  | Total | G1 | S | G2 | M |
| --- | --- | --- | --- | --- | --- |
| Arai et al. (2011)^b^ | 100.0 | 74.1 | 11.4 | 10.1 | 4.4 |
| Control | 100.0 | 74.2 | 11.3^c^ | 10.1^d^ | 4.4^d^ |
| Pax6 | 100.0 | 69.2 | 16.4^c^ | 10.1^d^ | 4.4^d^ |

Estimated length of cell cycle phases of Tis21+ aRG (h)

|  | T_C_ | T_G1_ | T_S_ | T_G2_ | T_M_ |
| --- | --- | --- | --- | --- | --- |
| Arai et al. (2011) | 15.8 | 11.7 | 1.8 | 1.6 | 0.7 |
| Control | 21.0 | 15.6 | 2.4 | 2.1 | 0.9 |
| Pax6 | 18.5 | 12.8 | 3.0 | 1.9 | 0.8 |

^a^ Means of 8 (control) and 9 (Pax6) cells, respectively.

^b^ Percentages were calculated from published cell cycle phase length data.

^c^ Data obtained from punctate PCNA staining (Figure 3F).

^d^ Percentage of cells in G2- and M-phase was taken from [52].
