# Supplementary material for: Sustained Pax6 Expression Generates Primate-like Basal Radial Glia in Developing Mouse Neocortex
Source: PLoS Biol. 2015 Aug 7;13(8):e1002217. doi: 10.1371/journal.pbio.1002217 (PMC4529158; doi:10.1371/journal.pbio.1002217)
Supplement: S2 Table — (DOCX) [file pbio.1002217.s019.docx]

Cell cycle length of self-renewing Tis21+ BPs (h)

|  | T_C_ (Imaging)^a^ |
| --- | --- |
| Control | 17.0 |
| Pax6 | 18.4 |

^a^ Mean of 2 (Control) and 4 (Pax6) cells, respectively (See Figure S9).
